# A genome sequence from a modern human skull over 45,000 years old from Zlatý kůň in Czechia

**DOI:** 10.1038/s41559-021-01443-x

**Published:** 2021-04-07

**Authors:** Kay Prüfer, Cosimo Posth, He Yu, Alexander Stoessel, Maria A. Spyrou, Thibaut Deviese, Marco Mattonai, Erika Ribechini, Thomas Higham, Petr Velemínský, Jaroslav Brůžek, Johannes Krause

**Affiliations:** 1 Max Planck Institute for the Science of Human History, Jena, Germany; 2 Max Planck Institute for Evolutionary Anthropology, Leipzig, Germany; 3 Institute for Archaeological Sciences, Archaeo- and Palaeogenetics, University of Tübingen, Tübingen, Germany; 4 Institute of Zoology and Evolutionary Research, Friedrich Schiller University Jena, Jena, Germany; 5 Oxford Radiocarbon Accelerator Unit, Research Laboratory for Archaeology and the History of Art, School of Archaeology, University of Oxford, Oxford, UK; 6 Centre Européen de Recherche et d’Enseignement des Géosciences de l’Environnement (CEREGE), Aix-Marseille University, CNRS, IRD, INRAE, Collège de France, Aix-en-Provence, France; 7 Department of Chemistry and Industrial Chemistry, University of Pisa, Pisa, Italy; 8 Department of Anthropology, National Museum, Prague, Czech Republic; 9 Department of Anthropology and Human Genetics, Faculty of Science, Charles University, Prague, Czech Republic

**Keywords:** Evolutionary genetics, Evolutionary biology

## Abstract

Modern humans expanded into Eurasia more than 40,000 years ago following their dispersal out of Africa. These Eurasians carried ~2–3% Neanderthal ancestry in their genomes, originating from admixture with Neanderthals that took place sometime between 50,000 and 60,000 years ago, probably in the Middle East. In Europe, the modern human expansion preceded the disappearance of Neanderthals from the fossil record by 3,000–5,000 years. The genetic makeup of the first Europeans who colonized the continent more than 40,000 years ago remains poorly understood since few specimens have been studied. Here, we analyse a genome generated from the skull of a female individual from Zlatý kůň, Czechia. We found that she belonged to a population that appears to have contributed genetically neither to later Europeans nor to Asians. Her genome carries ~3% Neanderthal ancestry, similar to those of other Upper Palaeolithic hunter-gatherers. However, the lengths of the Neanderthal segments are longer than those observed in the currently oldest modern human genome of the ~45,000-year-old Ust’-Ishim individual from Siberia, suggesting that this individual from Zlatý kůň is one of the earliest Eurasian inhabitants following the expansion out of Africa.

## Abstract (web summary)

The authors present the genome sequence of a >45,000-year-old female *Homo sapiens* individual from the site of Zlatý kůň, Czechia. Although radiometric dating of the human remains was inconclusive, the authors were able to use molecular methods to demonstrate that she was probably among the earliest Eurasian inhabitants following expansion out of Africa.

## Main

Only three genomes have been recovered from individuals that fall close in time to the settlement of Europe and Asia more than 40 thousand years ago (ka)^1,2^. A complete genome has been produced from the ~45,000-year-old remains of Ust’-Ishim, a Siberian individual who showed no genetic continuity to later Eurasians^3^. This contrasts with the ~40,000-year-old East Asian individual from Tianyuan whose genome is more closely related to many present-day Asians and Native Americans than to Europeans^4^. From Europe, only the partial genome of an individual called Oase 1 and dated to ~40 ka has been recovered, and this showed no evidence of shared ancestry with later Europeans^5^. However, Oase 1 carried more Neanderthal ancestry (6–9%) than other modern human genomes sequenced to date, owing to admixture with Neanderthals that occurred within the six generations before the individual lived.

**Fig. 1:**
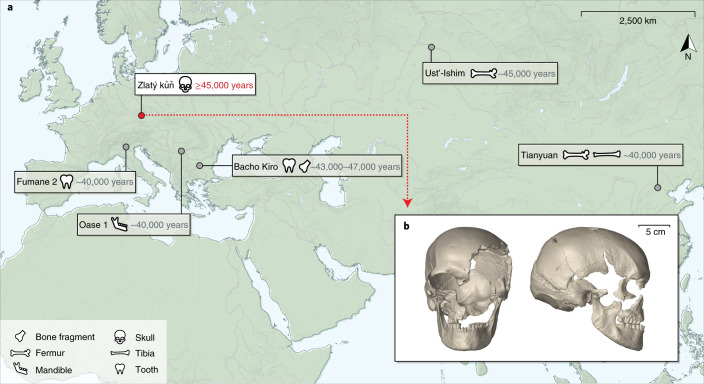
The Zlatý kůň fossil. **a**, Locations of the Koněprusy cave, where the Zlatý kůň human remains were found, and of other fossils with an age of at least ~40,000 years that yielded genome-wide data (Ust’-Ishim, Oase 1 and Tianyuan) or mtDNA (Fumane 2 and Bacho Kiro). **b**, Micro-computed tomography-based virtual reconstruction of the Zlatý kůň skull in frontal and lateral view. The map in **a** was created with QGIS^47^ using Natural Earth^48^ vector data.

**Extended Data Fig. 1:**
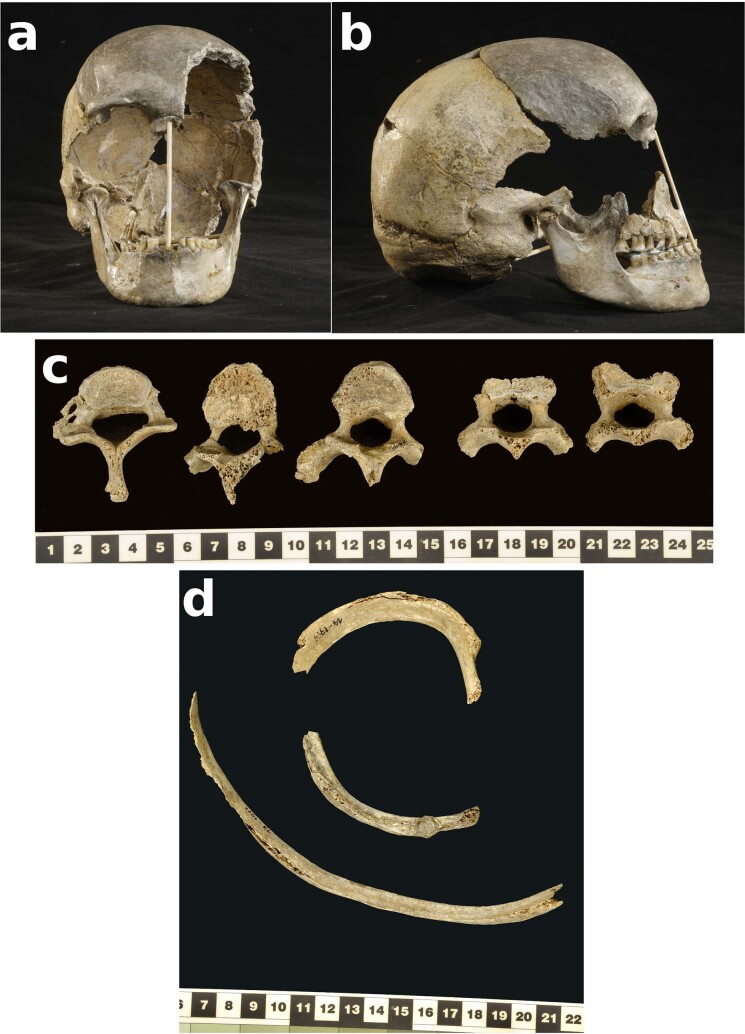
Skeletal remains from Zlatý kůň. **a**, and **b**, frontal and dorsal view, respectively, of the skull, **c**, vertebrae and **d**, costae. Credit: Martin Frouz (**a**,**b**); Marek Jantač (**c**,**d**).

Here, we study genome sequences generated from a largely complete ancient skull that was discovered alongside other skeletal elements in 1950 inside the Koněprusy cave system in present-day Czechia^6,7^ (Fig. 1, Extended Data Fig. 1 and Supplementary Section 1). All skeletal elements were found to originate from one adult female individual called Zlatý kůň (Golden Horse) after the hill on top of the cave system. Archaeological investigations ascribed the stone and bone tools retrieved from the cave to the early Upper Palaeolithic. However, the artefacts in association with this individual could not be confidently assigned to any specific cultural technocomplex^6,8^. The remains were first thought to be at least 30,000 years old in accordance with morphological and stratigraphic information and the type of associated faunal remains^8,9^. Moreover, damage on the left side of the frontal human bone was interpreted as biting and gnawing by hyenas, which went extinct from central Europe around 24 ka^10,11^. Whereas direct radiocarbon dating resulted in a much younger date of ~15 ka (12,870 ± 70 years bp; GrA-13696)^12^, a recent craniometric analysis that included a virtual reconstruction of the Zlatý kůň skull supports that the individual lived before the last glacial maximum^13^.

**Extended Data Fig. 2:**
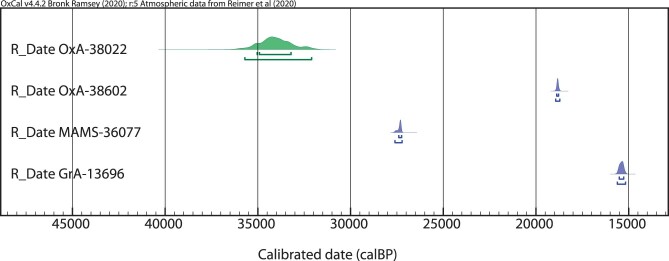
Four direct radiocarbon dates calibrated in years before present (calBP) with 68.3% and 95.4% probability. OxA-38022 refers to the HYP dating performed in Oxford. The dates were calibrated using OxCal 4.4^46^ and the IntCal20 calibration curve^49,50^.

In an attempt to clarify the age of Zlatý kůň, we radiocarbon dated a cranial bone fragment, resulting in a significantly older date of ~27 ka (23,080 ± 80 years bp; MAMS-36077) compared with the first direct date. A third date, comprising a solvent pre-wash treatment followed by ultrafiltration on the same bone fragment, produced a younger date of ~19 ka (15,537 ± 65 years bp; OxA-38602)^14^. The large discrepancies between the three direct dates suggest that the Zlatý kůň specimen is highly contaminated and that radiocarbon dating on bulk collagen may be unreliable (Supplementary Section 2 and Extended Data Fig. 2). We therefore extracted the amino acid hydroxyproline from leftover collagen to attempt to date a compound-specific fraction from the bone^15^. This yielded the oldest determination of ~34 ka (29,650 ± 650 years bp; OxA-38022). However, we suspect this is also artificially young due to the presence of trace exogenous contaminants derived from animal glue, as supported by genetic analysis (discussed below and in Supplementary Section 2). We therefore conclude that the hydroxyproline determination reflects a minimum age, with the true age likely to be much older.

**Fig. 2:**
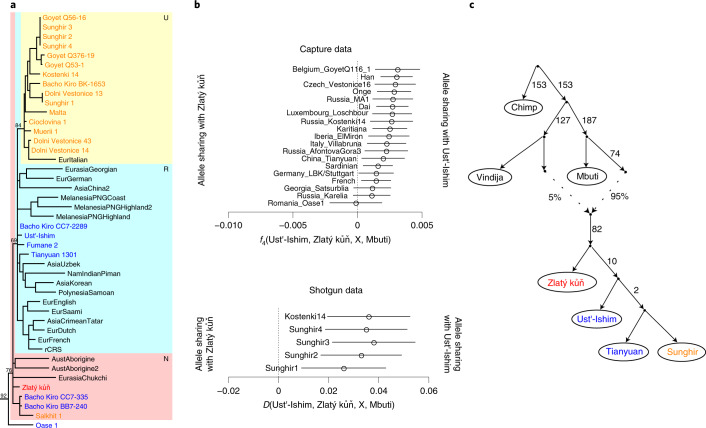
Genetic relationship with present-day and ancient humans. **a**, mtDNA haplogroup N of a maximum-parsimony phylogenetic tree of mtDNA from Zlatý kůň (red font), Upper Palaeolithic individuals ~40 ka or older (blue) or between ~37 and ~24 ka (orange), and present-day individuals (black) (the entire tree is presented in Extended Data Fig. 3). **b**, Analysis of nuclear sequences showing that Ust’-Ishim shares more alleles with European and Asian hunter-gatherers and later Eurasians than does Zlatý kůň. The error bars represent two standard errors. **c**, Admixture graph of the relationship inferred from the nuclear capture dataset. Zlatý kůň diverges earlier than Ust’-Ishim and the ancestors of later Eurasian populations represented here by the Upper Palaeolithic Tianyuan and Sunghir genomes (highest outlier |*Z*| = 3). A single gene flow event from Neanderthals into the ancestor of all tested modern humans fits the data. Colours for individuals follow the same scheme as in panel **a**.

**Extended Data Fig. 3:**
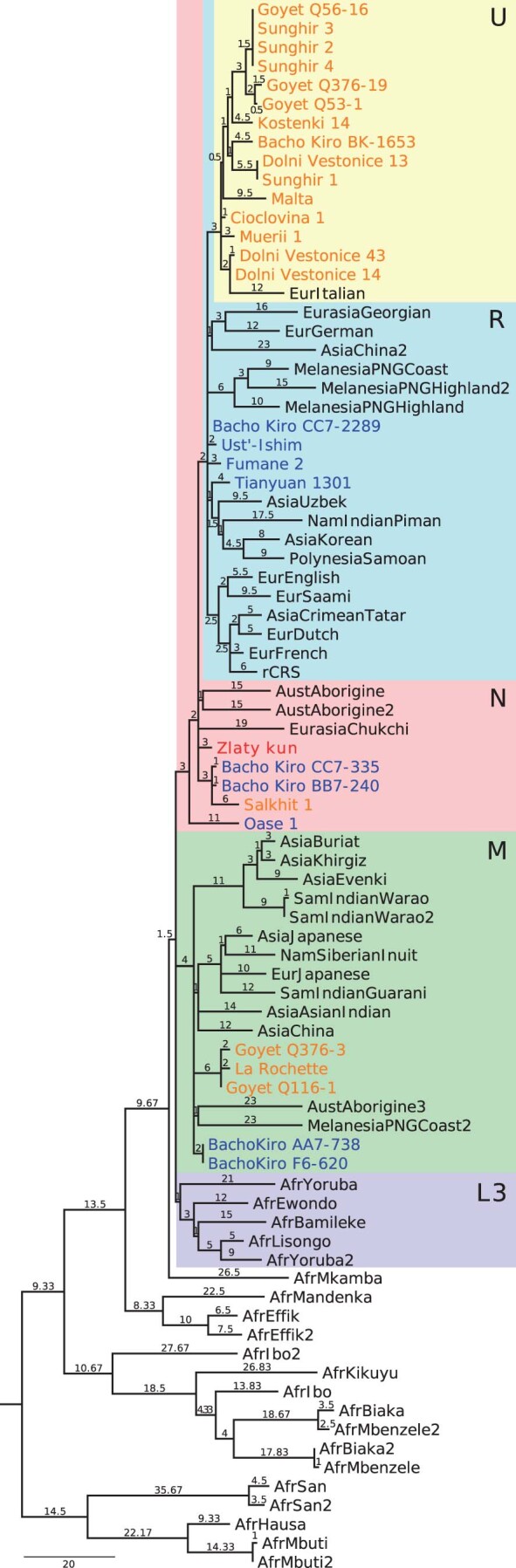
Maximum Parsimony tree of 54 modern-day worldwide mtDNA sequences, 28 Palaeolithic Eurasian mtDNAs, Zlatý kůň mtDNA and a Neanderthal mtDNA as outgroup. Text color relates to radiocarbon date, that is red (undetermined), blue (45–40 kya) and yellow (40–24 kya). Numbers show the number of substitutions along lineages; capital letters on the right side of the tree indicate mtDNA haplogroups.

We extracted DNA from ~15 mg bone powder from the Zlatý kůň petrous portion of the temporal bone and first enriched for and sequenced the mitochondrial genome (mtDNA) to ~150-fold coverage (Methods). Around 4% of the mtDNA sequences were estimated to stem from human contamination (Supplementary Section 3). The reconstructed mtDNA belongs to haplogroup N and its branch length, measured as the number of accumulated substitutions, is similar to those of the currently oldest sequenced modern human mtDNA genomes (Fig. 2a and Extended Data Fig. 3), including the recently published mtDNAs from Bacho Kiro, a cave in Bulgaria with remains dating to 43–47 ka^1^. Bayesian tip dating suggests that Zlatý kůň lived ~43 ka (95% highest posterior density = 31.5–52.6 ka).

**Extended Data Fig. 4:**
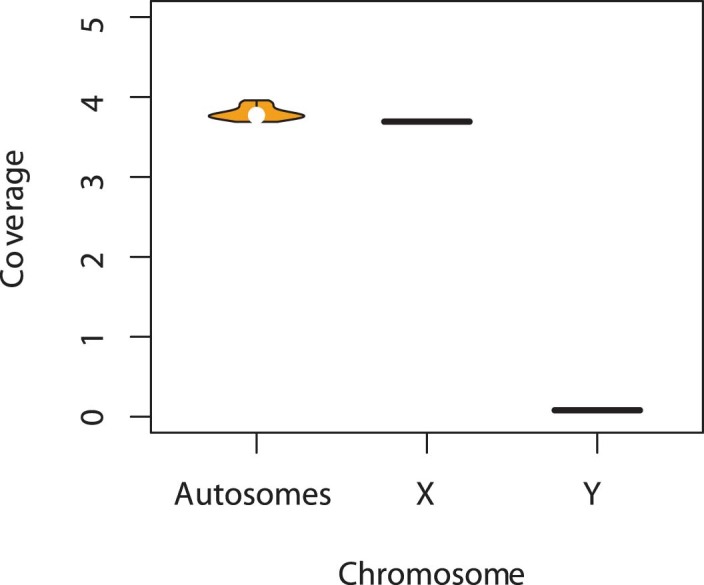
Coverage on sex chromosomes and autosomes. Average coverage by shotgun sequences for chromosome X, Y (horizontal dashes) and autosomes (violin plot).

To study the nuclear genome, we sequenced ~20 million DNA fragments after targeted enrichment with oligonucleotide probes for 1.24 million single nucleotide polymorphisms (SNPs)^16^. A total of ~678,000 targeted SNPs (54%) were covered at least once after genome-wide enrichment (capture dataset). In addition, we sequenced ~4 billion random DNA fragments from the same DNA library of Zlatý kůň, resulting in ~3.8-fold genomic coverage (shotgun dataset). In line with the sex assignment based on morphology^13^, the X chromosome and autosomes showed similar coverage, indicating that Zlatý kůň is female (Extended Data Fig. 4). The presence of Y chromosomal sequences suggested that up to 4% of the nuclear DNA sequences in the shotgun dataset originate from male contamination. Estimates based on linkage disequilibrium^17^ suggest that nuclear contamination is <1% in the capture dataset and ~2% in the shotgun dataset (Supplementary Section 4). The majority of the Zlatý kůň shotgun sequences (~3.2-fold out of ~3.8-fold total) have been generated from a single-stranded DNA library that allows for the quantification of contamination with an explicit model of DNA damage in the DNA molecules^18^. This model yielded an estimate of contamination of 0.1% (s.e. = ±2.0%) (Supplementary Section 4).

**Extended Data Fig. 5:**
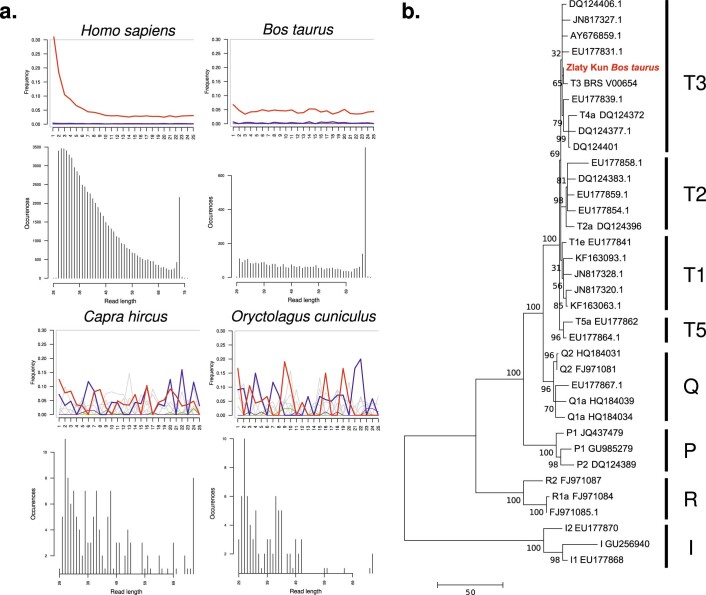
Characteristics of *Bos taurus* mtDNA sequences and genome. **a**, C-to-T substitutions at the 5′ end and read length distributions of mtDNA sequences mapping specifically to the mtDNA references of *Homo sapiens*, *Bos taurus*, *Capra hircus* and *Oryctolagus cuniculus*. Note that a single-end 75-cycle chemistry kit was used for the retrieval of the reported sequencing reads and, therefore, DNA fragments of length 76 bp or above have not been sequenced at their full length and are binned here together. **b**, Maximum parsimony tree of 35 present-day and ancient cattle mtDNAs and the *Bos taurus* mtDNA reconstructed from the deep shotgun sequencing of the DNA extracted from the Zlatý kůň petrous bone. Cattle haplogroups are listed on the right side of the tree.

We used the non-human fraction of the shotgun data to further investigate whether the use of animal glue could have influenced our attempts at radiocarbon dating of the Zlatý kůň skull. Searching a metagenomic database, we found that the highest proportion of non-human mammalian shotgun sequences aligned to bovids (Supplementary Section 2). We were able to reconstruct ~95% of the bovid mtDNA from the shotgun sequences of the single-stranded library and found that it falls within the most common modern European cattle haplogroup^19^ in a phylogenetic analysis (Extended Data Fig. 5). Low levels of substitutions that are indicative of ancient DNA damage suggest that the cattle sequences do not derive from present-day laboratory contaminants (Extended Data Fig. 5). Taken together, these results suggest that the Zlatý kůň skull has been preserved with glue from cattle that penetrated into the sequenced petrous bone.

**Extended Data Fig. 6:**
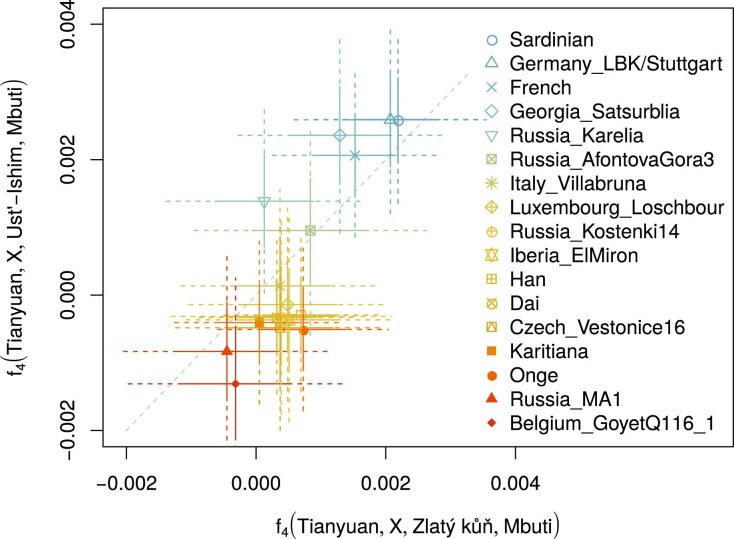
Comparable signal of Basal Eurasian ancestry in Zlatý kůň and Ust’-Ishim. Zlatý kůň’s asymmetric sharing of alleles with some Europeans compared to Asians (x-scale) is also observed to a comparable degree in Ust’-Ishim (y-scale). The ancient genome from Tianyuan is used as a proxy for Asians. The solid and dash error bars correspond to one and two standard errors, respectively.

**Extended Data Fig. 7:**
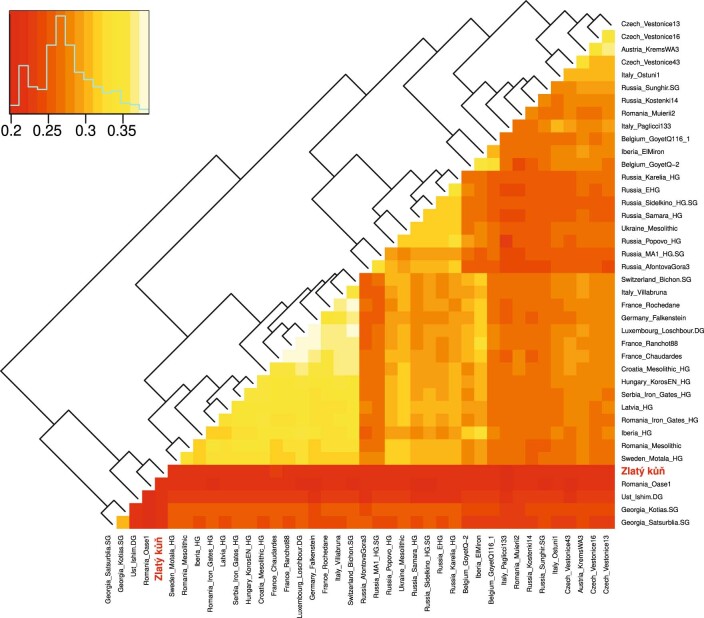
Heat-map matrix of pairwise f3-outgroup statistics with Mbuti as outgroup. Zlatý kůň shows no closer affinity to Ust’-Ishim and Oase 1 or to any later hunter-gatherer individuals.

To gain insight into the genetic relationship of Zlatý kůň to present-day and ancient individuals, we calculated summary statistics based on the sharing of alleles (*f*_3_, *f*_4_ and *D* statistics^20^) with our capture and shotgun datasets. We first compared Zlatý kůň with present-day European and Asian individuals using an African population (Mbuti) as an outgroup and found that Zlatý kůň shares more alleles with Asians than with Europeans (Extended Data Fig. 6). A closer relationship to Asians has also been observed for other Upper Palaeolithic and Mesolithic European hunter-gatherers compared with present-day Europeans and can be explained by ancestry in present-day Europeans from a deeply divergent out-of-Africa lineage referred to as basal Eurasian^21^. European hunter-gatherers generally do not carry basal Eurasian ancestry, whereas such ancestry is widespread among ancient hunter-gatherers from the Caucasus, Levant and Anatolia^22–24^. When we tested European hunter-gatherers without basal Eurasian ancestry against ancient and present-day Asians, we found that none of these comparisons indicate a closer relationship of Zlatý kůň with either group (Supplementary Sections 5 and 9 and Extended Data Fig. 7). This suggests that Zlatý kůň falls basal to the split of the European and Asian populations.

To date, only two ancient Eurasian genomes have been produced from individuals who, like Zlatý kůň, appear to fall basal to the split of Europeans and Asians: Ust’-Ishim and Oase 1. To test whether Zlatý kůň derives from the same population as Ust’-Ishim, we tested for a closer relationship to it compared with other ancient Eurasian hunter-gatherers^24–26^. Interestingly, we found that Ust’-Ishim shares more ancestry with later Eurasian individuals (Fig. 2b). This suggests that Zlatý kůň was part of a population that split earlier from the population that later gave rise to Ust’-Ishim and other Eurasian populations (Fig. 2c). Due to the limited data for Oase 1, we are unable to clarify whether Zlatý kůň and Oase 1 derive from the same or separate populations.

**Fig. 3:**
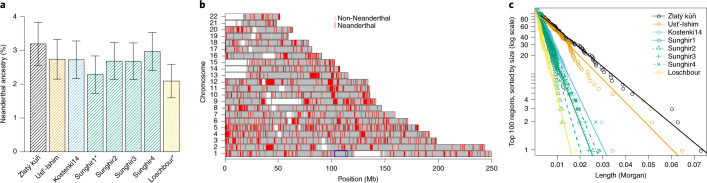
Neanderthal ancestry in Zlatý kůň and ancient Eurasian genomes. **a**, Estimate of Neanderthal ancestry in ancient Eurasian hunter-gatherer genomes. The error bars indicate two standard errors. Individuals whose names are marked with an asterisk fall outside of the error bars for Zlatý kůň. **b**, Segments of Neanderthal ancestry in Zlatý kůň. The blue box shows the location of a desert of Neanderthal ancestry in present-day non-Africans. **c**, Length of the 100 largest Neanderthal segments in the genomes of Zlatý kůň and other Upper Palaeolithic and Mesolithic Eurasian hunter-gatherers. The *y* axis is logarithmic and the lines indicate a linear fit. The colours are as in **a**.

Around 6–9% of the genome of Oase 1 is derived from Neanderthals, compared with 2–3% in present-day and ancient Eurasians^5,27,28^. To test whether a higher contribution is also present in Zlatý kůň, we calculated Neanderthal ancestry on the shotgun dataset as the excess of shared alleles with a Neanderthal as opposed to an African and normalized this quantity by the expected sharing between two Neanderthals as opposed to an African (*f*_4_ ratios^20^; Supplementary Section 6). Zlatý kůň is estimated to carry 3.2% (s.e. = ±0.32%) Neanderthal ancestry, which is the highest value among six Upper Palaeolithic and one Mesolithic Eurasian hunter-gatherers with genome-wide data (range = 3.0–2.1%). However, this difference was not significant at a level of two standard errors for five out of seven comparisons (Fig. 3a).

**Extended Data Fig. 8:**
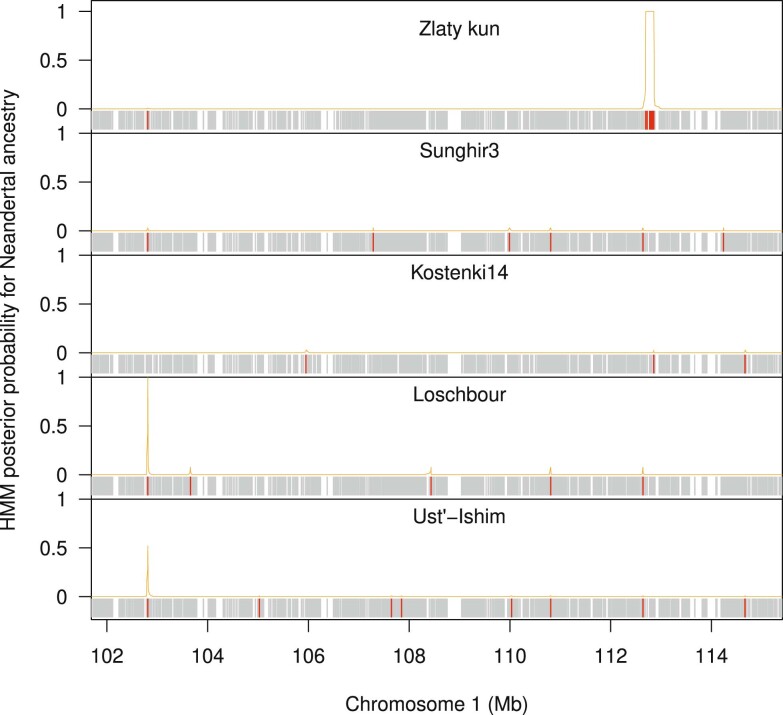
Neanderthal desert region on chromosome 1 in Zlatý kůň and 5 high-coverage ancient genomes. Each panel shows Neanderthal-shared variants in red and non-shared variants in gray. The orange line indicates the posterior decoding probabilities for Neanderthal ancestry from the hidden Markov model approach. The Neanderthal ancestry segment in Zlatý kůň (chr1:112696231-112855758) is supported by 17 Neanderthal shared variants. A short region, spanning only 6 bp (chr1:102812075-102812081) is called in Loschbour and contains four sites that are Neanderthal shared. The short length and high density of sites do not support a true Neanderthal origin.

To study the distribution of Neanderthal ancestry along the genome, we first determined 430,075 sites on autosomes where the genome of a high-coverage European Neanderthal carries on both chromosomes a variant that is not observed in more than 99.9% of present-day Africans and great ape outgroups (Supplementary Section 7). Of the 166,721 sites that are covered by Zlatý kůň shotgun data, 4,480 (2.7%) carried the Neanderthal allele. Neanderthal sites in the Zlatý kůň genome cluster into segments where they occur at high frequency (~50%; Fig. 3b) and we used this clustering to label segments of likely Neanderthal ancestry with a hidden Markov model (Supplementary Section 7). One of the Neanderthal segments falls within a large region on chromosome 1 that shows little to no evidence of Neanderthal ancestry in present-day humans^29^ (Extended Data Fig. 8). This suggests that this desert of Neanderthal ancestry had not been fully formed at the time Zlatý kůň lived.

**Extended Data Fig. 9:**
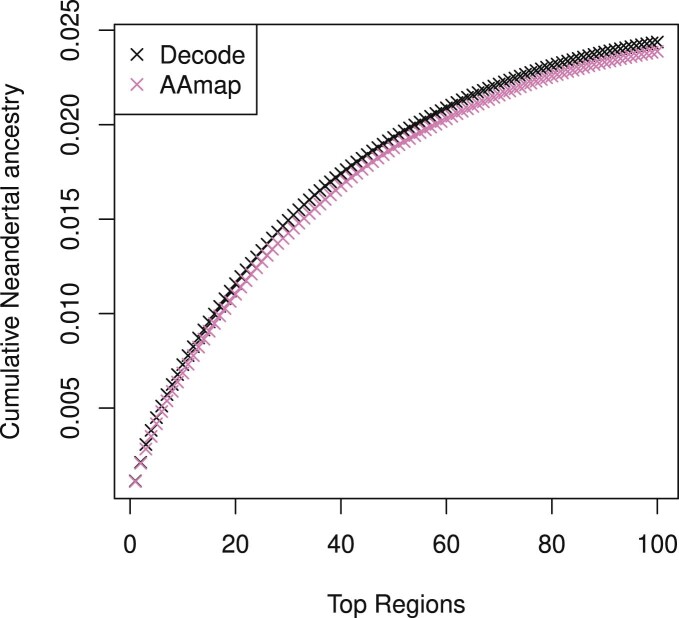
Fraction of the Zlatý kůň genome covered by the top 100 called introgressed regions sorted by length. The fraction of the genome covered by Neanderthal ancestry (y-scale) was calculated by dividing the cumulative length in Morgan of introgressed regions up to and including the n^th^ longest region shown on the x-scale by two times the total length of the autosomes in Morgan. Results are shown for the African American or Decode recombination maps.

Recombination will break long Neanderthal segments into shorter segments over time. To gain more insight into the timing of Neanderthal admixture in Zlatý kůň, we scaled the length of the Neanderthal segments using either an African American map (AA map)^30^ or the Decode recombination map (deCODE map)^31^ and compared the genetic length of the 100 longest segments in Zlatý kůň with those identified in other early Eurasian hunter-gatherers using the same method (Extended Data Fig. 9). We found that Zlatý kůň carries segments that are on average longer than those of all other Eurasian hunter-gatherers (Fig. 3c). Assuming that recombination breaks Neanderthal ancestry into shorter segments every generation (see ref. ^5^ and Supplementary Section 8), we estimated that the last admixture with Neanderthals occurred ~70–80 generations before Zlatý kůň lived (AA map: 74 generations (95% confidence interval (CI) = 61–89); deCODE map: 78 generations (95% CI = 64–94)). In contrast, the genome of the currently oldest sequenced modern human, Ust’-Ishim, yielded significantly higher estimates of 94 generations (95% CI = 77–113) and 99 generations (95% CI = 81–119) using the AA map and deCODE map, respectively. Estimating ages using segments called by admixfrog (a software for inferring local Neanderthal ancestry^32^) yielded comparable results (Supplementary Section 8).

To estimate the time of admixture independent of calling Neanderthal ancestry segments, we applied a previously published method that is based on the correlation of the state of Neanderthal informative sites over increasing distances^33^. Applying this method to the Zlatý kůň shotgun dataset yielded an estimate of 63 generations since admixture (s.e. = ±0.6), whereas Ust’-Ishim was estimated to have lived 84 generations after the admixture (s.e. = ±1.3).

**Extended Data Fig. 10:**
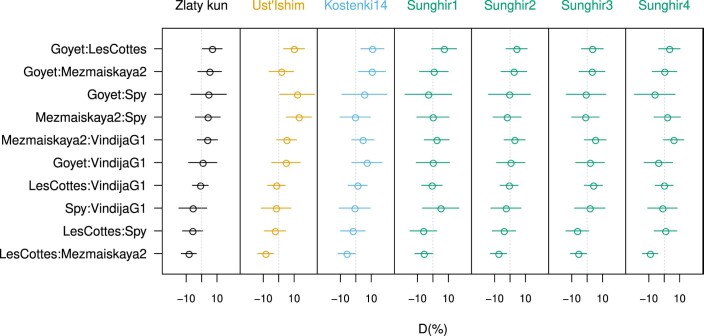
Pairwise comparison of low-coverage Neanderthals in their sharing of alleles with European and Asian hunter-gatherers. Shown are the D-statistics of the form D(Mbuti, Hunter-Gatherer, Neanderthal1, Neanderthal2). The Hunter-Gatherer corresponds to the columns; rows show the two compared Neanderthals in order Neanderthal1: Neanderthal2. Error bars correspond to two standard errors.

Most of the Neanderthal ancestry in present-day and ancient humans probably originates from a common admixture event with a group of Neanderthals who were more closely related to European Neanderthals than to a Neanderthal from the Altai Mountains^28^. To test whether the Neanderthal ancestry in Zlatý kůň shows the same relationship, we used *D* statistics to compare the sharing of alleles with the high-coverage genomes of a European and an Asian Neanderthal in addition to five published low-coverage genomes from late Neanderthals from Europe^34^. Zlatý kůň showed no significant difference compared with Ust’-Ishim in its sharing of alleles with late Neanderthals, in line with the similar proportion of Neanderthal ancestry in these two hunter-gatherers (Supplementary Section 6 and Extended Data Fig. 10).

Assuming a common Neanderthal admixture event, these results suggest that Zlatý kůň is of approximately the same age as the ~45,000-year-old Ust’-Ishim individual or up to a few hundred years older. However, if a second Neanderthal admixture event affected Ust’-Ishim after the initial common Neanderthal admixture, as was previously suggested^33^, Zlatý kůň could be even several thousands of years older than Ust’-Ishim. We have not found support for a second Neanderthal admixture event in the Zlatý kůň data (Supplementary Section 8).

The genetic identity of the modern humans who colonized Eurasia before ~40 ka remains largely unknown. Here, we sequenced and analysed the genome of an early European and determined that she was part of a population that formed before the populations that gave rise to present-day Europeans and Asians split from one another. Our estimated age of ~45,000 years or even older could make Zlatý kůň the oldest European individual with a largely preserved skull^13^. As for Ust’-Ishim and Oase 1, Zlatý kůň shows no genetic continuity with modern humans who lived after ~40 ka. It is possible that this discontinuity is linked to the Campanian Ignimbrite eruption ~39 ka that severely affected the climate in the Northern Hemisphere and that may have reduced the viability of Neanderthals and early modern humans in large parts of western Eurasia^35,36^. Whether the modern humans who lived before the turnover event, such as the Oase 1 and Bacho Kiro individuals^3,5^, belonged to the same early European population can only be resolved with further genome-wide data from those individuals^37^. Future genetic studies of these and other early European individuals will thus help to reconstruct the history of these first modern humans who expanded into Eurasia after the out-of-Africa event and before the major dispersal that gave rise to modern-day non-African populations.

*Note added in proof:* We refer readers to related work by M. Hajdinjak et al.^37^ who analysed nuclear sequences from Bacho Kiro individuals that dated to around 45,000 years ago.

## Methods

### Laboratory procedures and shotgun sequencing

All laboratory procedures were conducted in the dedicated ancient DNA facilities of the Max Planck Institute for the Science of Human History in Jena, Germany. The Zlatý kůň cranium was sampled from the base of the cranium with a dentist drill after removal of a thin layer of bone powder. Two aliquots of ~15 mg bone powder were sampled and one was used to extract DNA using an established protocol^38^. A double-stranded DNA library with partial uracil-DNA glycosylase treatment was generated from 25% of DNA extract^39^. After quantification, the library was double indexed^40^ and quantified again to establish the number of PCR cycles necessary to reach the amplification plateau^41^. The resulting library was diluted and shotgun sequenced on two lanes of an Illumina HiSeq 4000 platform for 2 × 50 cycles. An additional 30% of the same extract was used to generate a single-stranded library^42^ on an automated liquid handling system (Bravo; Agilent Technologies). After indexing, the library was amplified for 30 cycles followed by a reconditioning PCR to remove heteroduplexes. The resulting library was diluted and shotgun sequenced on an entire flow cell (eight lanes) of an Illumina HiSeq 4000 platform for 1 × 75 cycles.

### Basic processing and sequence alignment of shotgun data

A total of ~580 million paired-end reads from the double-stranded library matched the correct indices, allowing for up to one mismatch per index, and were further processed with EAGER^43^. Adapter sequences were trimmed, filtered for a minimum length of 30 base pairs (bp) and mapped to the hg19 human reference genome using BWA^44^ with the following parameters: -n 0.01 -l 16500. Approximately 10% of sequences mapped with an average fragment length of 49 bp. These sequences were filtered for a minimum mapping quality of 30 and duplicates were removed using Dedup^43^. Sequences showed C to T exchanges to the human reference, indicative of ancient DNA damage, with a frequency of ~13% at both terminal positions^45,46^. The first and last two bases of shotgun sequences were masked (set to N) to reduce the effect of damage-associated substitutions on subsequent analyses.

An additional ~3.355 billion single-end reads were produced from the single-stranded library on a dedicated run. All sequences were processed using EAGER with the same parameters as above, except the mapping quality filter was set to 25. Around 15% of reads mapped to the human reference, with an average fragment length of 46 bp and 30% of C to T substitutions at the 5′ molecule termini.

### Target enrichment, sequencing and processing

The indexed library was further amplified to perform targeted enrichment of both the complete mtDNA (mtDNA capture)^5^ and ~1.24 million nuclear SNPs (1240K capture)^16^ followed by HiSeq paired-end sequencing and index filtering, resulting in 600,000 and 20 million reads, respectively. MtDNA capture and 1240K capture data were mapped against the mtDNA reference sequence (the Revised Cambridge Reference Sequence (rCRS)) using CircularMapper^43^ and the human reference genome (GRCh37/hg19), respectively, with the same parameters as above and the mapping quality filter set to 30.

A random allele was drawn from the 1240K capture data using PileupCaller in pseudohaploid mode (https://github.com/stschiff/sequenceTools). The calling of transversions among the 1.24 million target SNPs considered full sequences, whereas 2 bp at both termini of sequences were trimmed before calling transition SNPs. Finally, the resulting calls were merged with a large genotype dataset of ancient and present-day individuals for the same set of ~1.24 million SNPs (https://reich.hms.harvard.edu/allen-ancient-dna-resource-aadr-downloadable-genotypes-present-day-and-ancient-dna-data; version 37.2).

### Reporting Summary

Further information on research design is available in the Nature Research Reporting Summary linked to this article.

## Supplementary information

Supplementary Information Supplementary Sections 1–10, Figs. 1–15 and Tables 1–32.

Reporting Summary

## Data Availability

All of the sequence data generated and analysed during the current study are available in the European Nucleotide Archive under study accession number PRJEB39040.
