# Supplementary material for: A genome sequence from a modern human skull over 45,000 years old from Zlatý kůň in Czechia
Source: Nat Ecol Evol. 2021 Apr 7;5(6):820–5. doi: 10.1038/s41559-021-01443-x (PMC8175239; doi:10.1038/s41559-021-01443-x)
Supplement: Supplementary file 1 — Supplementary Sections 1–10, Figs. 1–15 and Tables 1–32. [file 41559_2021_1443_MOESM1_ESM.pdf]

nature portfolio

https://doi.org/10.1038/s41559-021-01443-x

Supplementary information

A genome sequence from a modern human skull over 45,000 years old from Zlatý kůň in Czechia

In the format provided by the authors and unedited

Supplementary Information

A genome sequence from a modern human skull over 45,000 years old from Zlatý kůň in Czechia

Kay Prüfer, Cosimo Posth, He Yu, Maria A. Spyrou, Alexander Stoessel, Thibaut Deviese, Marco Mattonai, Erika Ribechini, Thomas Higham, Petr Velemínský, Jaroslav Brůžek, Johannes Krause

Correspondence to: pruefer@eva.mpg.de, cosimo.posth@uni-tuebingen.de, krause@eva.mpg.de

Table of Contents

Archeological description....3

Radiocarbon Dating and Animal Contamination....12

Mitochondrial Analysis....18

Phenotype extrapolation, Genetic Sexing and Nuclear Contamination Rates....22

F3-, F4-statistics and qpGraph analyses of capture data and relationship to modern human populations....25

Neandertal ancestry estimates and relationship to late Neandertals from shotgun data....43

Neandertal ancestry along the genome....50

Dating Neandertal introgression....58

Relationship to Early Upper Palaeolithic hunter gatherers inferred from shotgun data....61

Computer tomography scan and virtual reconstruction....67

References....70

Supplementary Information S1

Archeological description

History of discovery

The Koněprusy cave system in Zlatý kůň ("Golden Horse") hill is the most extensive in Bohemia, comprising three levels and a total of 1.5 km of corridors. It was discovered, after blasting at a mine for limestone to be used in making cement, on September 14th 1950. In the Middle Ages there was a counterfeiting workshop (mint) on the upper level[1]. The axis of the middle floor is the so-called 'Old Passage', which runs north-south and in its central part is 20 m wide and 5 m high. West of the Old Passage lie the largest domes. Furthest north is the Prošek (Main) Dome, a space 50 m long and 20 m wide, in the middle of which is a massive stalagmite. This stands at the foot of a talus cone falling from three chimneys on the west side. The most interesting palaeontological and archaeological finds come from the talus cone[2,3].

The first human fossil record, of the occipital part of a skull, on November 17th 1950, was made at the foot of the talus cone, at the interface of the yellow-grey scree and the red clayey earth[4,5]. Shortly afterwards, on December 18th 1950, an 'expert commission' was convened to assess the significance of the site; it included the anthropologist E. Vlček, the archaeologist F. Prošek, the geologist J. Kukla, and the Quaternary biologist and malacologist V. Ložek. Given the presence of the fossil human calvaria on the surface of the talus cone, a well-documented archaeological excavation began in 1951, led by František Prošek[2,6].

The find was complemented by the discovery of vertebrae in days on April 6th-9th 1951, a rib fragment also being found on the same day. A mandible fell out of the excavated profile on April 10th 1951. A left zygomatic bone was discovered on April 14th 1951[7]. The second excavation season in 1952 yielded the following finds, in this chronological order and with precise stratigraphic localisation: April 28th, 1952 - a thoracic vertebra; May 2nd - part of a frontal bone (Supplementary Figure 1), a calvaria left in situ and removed after a meeting of the commission on May 13th; June 19th - a rib fragment; June 21st - another rib fragment[8]. A fragment of a right maxilla and isolated teeth were recovered on May 21st 1953[8].

The original conclusion, that the human remains recovered came from 2-3 individuals with a range of archaic traits that could be dated to the basal layers of Würm 2[8], was later corrected, and the skeletal remains are now assigned to a single individual which we refer to as Zlatý kůň in this paper[9,10].

Archaeological/geological context

A 2 m wide trench was excavated at the longest point of the talus cone, divided into square quadrats, with a 1m wide control stretch left intact. Two longitudinal profiles 29 m long were obtained from each side of the trench, along with 12 cross profiles, which were photographed, measured and drawn at a scale of 1:20[6].

The stratigraphic trench from research in 1950-1952 was filled and concreted in connection with work to make the area accessible to the public. The results were however supplemented by trenches dug in 1971-72 and 1974-75. The former fully worked out the sediments of the western part of the cave. The latter was sunk in the upper part of the talus cone.

Morphological reconstruction of the talus cone at Prošek's Dome in the Zlatý kůň caves is based on the original field documentation, now deposited at the Palaeolithic and Palaeoethnology Department of the Institute of Archaeology of the Czech Academy of Sciences at Dolní Věstonice[11].

Stone and bone artifacts

The rare associated stone and bone artifacts were concentrated in the mantle of a talus cone, in a restricted area of about 1 square meter[6,11,12]. However, the association of these artifacts with the human skeleton is likely, but remains uncertain. The preserved tools include four artifacts of metamorphic schist, a quartz flake with traces of retouching, a flake from brown cornea. In addition, several bone artifacts, tools were also found - two bone flakes with notch, one bone flake with grooves, a bone flake with a point tip, bone point tip with rap - and a tertiary clipboard of bivalve mollusc (*Glycimeris glycimeris pilosa L*) with a natural perforation[11].

Most tools are unretouched, so chronological and cultural classification is not possible. The only piece that might be diagnostic for chronology and cultural attribution, a bone point tip (a fragment of the rib of a large mammal), is dubious as an artifact[11]. The archaeologists ascribed above mentioned tools to the Early Upper Paleolithic, mostly pre-30,000 years B.P., but this attribution is not on the basis of any typological data[6,12,13].

Morphology

A brief anatomical description of the human skeletal remains from Zlatý kůň was provided in Czech by Emanuel Vlček in a series of publications[5,7,8,14]. He subsequently brought all this information together in a more detailed contribution in 1957[4], where he noted that from a morphological point of view, the human fossil from Zlatý kůň show a range of traits similar to a number of Upper Palaeolithic human remains from elsewhere in the Czech Republic, particularly the anatomically modern human fossils from Předmostí and Brno II. More exactly, these analogies are in the construction of the cranial vault - in the shape and inclination of the frontal bone, the vaulting (arched) of the frontal bone, the development of the superciliary arches, the character of the nasal root, the occipital ridge, the nuchal area, the posterior elongation of the occipital area, the configuration of the lower edge of the nasal aperture with the formation of equivalent *fossae praenasales*, and in the alveolar prognathism of the maxilla (e.g. [4]). By contrast, morphologically the Zlatý kůň material differs reasonably clearly from the Aurignacian human remains from Mladeč and the Gravettian skulls from Dolní Věstonice and Brno III[4].

According to Vlček, in comparison with Upper Palaeolithic skulls, the remains from Zlatý kůň display several more archaic morphological traits, e.g. the greater thickness of the cranial vault, the character of the protruding supraorbital arches and the size of the temporal bones in the context of the weak expression of the mastoid process (e.g. [4]). He therefore assumed that the Zlatý kůň finds "represent a more archaic stage of the modern sapient form, and thus anatomically modern human".

The structure and size of the preserved post-cranial bone fragments from Zlatý kůň – just seven cervical vertebrae, four thoracic vertebrae and three ribs – also match those of anatomically modern humans[15]. No more precise conclusions can be drawn, given the preservation of the fragments.

Similar conclusions were arrived at up until the end of the 20th century. For example, Churchill & Smith (2000)[16] write:

> "The Zlatý kůň cranium is similar to other early modern Europeans in having well-developed occipital bunning, a robust supraorbital region divided into a superciliary arch and supraorbital trigone, an "en maison" vault contour in coronal profile, zygoma without columnar frontal processes, and a maxilla with a narrow nasal aperture and a clear although weakly expressed canine fossa. The specimen also preserves a robust mandible with a distinct mental eminence and mental

> trigone, a moderately retreating symphysis. Although the specimen does exhibit a weakly developed suprainiac fossa on the occipital, the modern morphology of Zlatý kůň is indisputable. The geological age and cultural associations of the Zlatý kůň hominid are not entirely certain, the morphological affinities of this specimen are quite clear."

A study by Rmoutilová et al. (2018)[17] virtually reconstructed the missing parts of the skull of the Zlatý kůň individual, allowing collection of the main cranial measurements and verification of their sex attribution and morphological affinity. Using a population-specific approach developed on cranial measurements collected from the literature on reliably sexed European Upper Palaeolithic specimens, linear discriminant analysis confirmed with high probability (0.98) the previous assignment to the female sex. The small size of the reference sample should however be borne in mind. The Zlatý kůň specimen clearly falls within the range of Upper Palaeolithic cranial variation. Despite its first radiocarbon dating[18], Zlatý kůň exhibits morphological affinity with the pre-LGM humans.

Taphonomic history of the specimen after excavation

There is relatively little information on the conservation treatments applied to the bones. Vlček and Zázvorka mention that the bones were first mechanically cleaned under water with small brushes. They were then dried and preserved using different chemicals[4,19].

Human and animal bones lying on the surface of the debris cone were of a light ochre colour whereas those from inside it were very dark gray to gray-brown, due to the higher concentration of manganese in the soil. The bone tissue shows marked fossilisation[4].

Damage on the left side of the frontal bone has the character of biting and gnawing by a hyena or other predator[8]. Dietrich & Žák (2006) assumed, given the then ^{14}C dating of Zlaty kůň to the Magdalenian period (AMS ^{14}C age of 12,870 ± 70 years BP)[18], that wolves were probably responsible, since hyena became extinct in Central Europe around 24,000 BP[20].

Dating using stratigraphy and faunal remains

Following the discovery, the fossil was often assigned to the Würm II[6,21] (~70-38kya (see pp.102f. in ref.[22]) and broadly matching Marine Oxygen Isotope Stage 3, i.e. 59-24kya[23,24]), in accordance with the layers containing the human remains, stone artifacts and faunal remains. Mollusk shells found at the base of the debris matched the W1-2 interstadial[6], i.e. ~70kya[22].

In speleological circles the opinion spread that experts had dated the human remains to 30-70,000 years old[25].

A series of palaeontological studies at the end of the 20th century also considered the Zlatý kůň fossil to be from the Early Upper Palaeolithic. Publications from the 1950s also state: "In the further part of the talus cone, at the base of the rubble, abundant mollusc shells were found (Mollusc fauna = MF_3). All of the earlier layers were covered in sediment stemming from the northern chimney, as shown by the scattered human and vertebrate bones. Later, debris filled most of the chimney, with the remainder filled by loess. These observations indicate that the human remains, variously scattered through the debris layers, are contemporary with the beginning of the sedimentation of this debris, and were transported during this sedimentation. In the final period of debris sedimentation, the chimney was fully closed so that the human remains were already in their locations by this time. These observations indicate that the human remains are from the Pleistocene, although a more precise dating based on stratigraphy is impossible. "The subsequent introduction of human remains into the Main Dome of the Koněprusy Caves can be ruled out, as all the chimneys were already blocked, and the crawlway into the Main Dome penetrated by its discoverers had long since been closed" [12].

The faunal assemblage further supported an older age of the human remains in the Prošek Dome[12,26]. According to analysis of the vertebral fauna (VF_3: *Canis lupus, Crocuta crocuta spelaea, Equus caballus, Coelodonta antiquitatis, Rangifer tarandus, Marmota sp.*), the age of the debris layers that also contained human remains was established as W1 or W2 (W = Würm interstadial)[12,26].

"The richest MF_3 molluscan fauna lie at the base of the debris stratigraphy. Here too there is a predominance of the forest elements *Monacha incarnata, Eulota fruticum, Arianta arbustorum, Isognomostoma personatum, Helicodonta obvoluta, Helix pomatia. Helicodonta obvoluta,* and *Helix pomatia* – warmth-loving woodland elements of more southerly origin - that shows that the climate must at the very least have been as warm as that of the present [...]. […] The dominant formation was forest, locally interrupted by enclaves of a steppe-like character." [12] “The debris layers with human remains and archaeological finds can, according to the mammalian fauna with hyena and marmot remains, be attributed to the W 2[, i.e. ~70-38kya[22]]. The molluscan fauna at the base of these strata evidently represent the W1-2 interstadial [, i.e. ~70kya[22]]"[6].

Churchill et al. (1999) also considered the Zlatý kůň fossils to be older than 30,000 years (Table 1, p. 33 in ref. [27]). In 2000 Churchill & Smith[16] said: "The cave produced a partial skeleton of a robust yet morphologically modern individual, as well as artifacts of an Upper Palaeolithic character, distributed within a large talus cone in the main chamber. The deposits in Koněprusy accumulated as debris fell through a chimney in the cave during early Upper Pleniglacial times (early Würm IIIb) (Prošek et al., 1952; Vlček, 1957; Svoboda, 2000)." Note that the reference to Würm IIIb does not match the data in the original Czech-language publications[4,11] and possibly originates from a translation error. The age of the human remains was assigned to the first half of the W II stadial[12].

Repository of the Zlatý kůň fossil material

The human fossil material is stored in the Department of Anthropology of the National Museum, Prague[10,21].

The palaeontological material – faunal bones - from Zlatý kůň is stored in a) the Department of Palaeontology of the National Museum in Prague[6], and b) The Museum of the Bohemian Karst (originally the District Museum) in Beroun[28].

Supplementary Figure 1.

The part of frontal bone in situ which was discovered on May 2nd 1952 (photo Emanuel Vlček, the Archives of the Anthropological Department, National Museum, Prague).

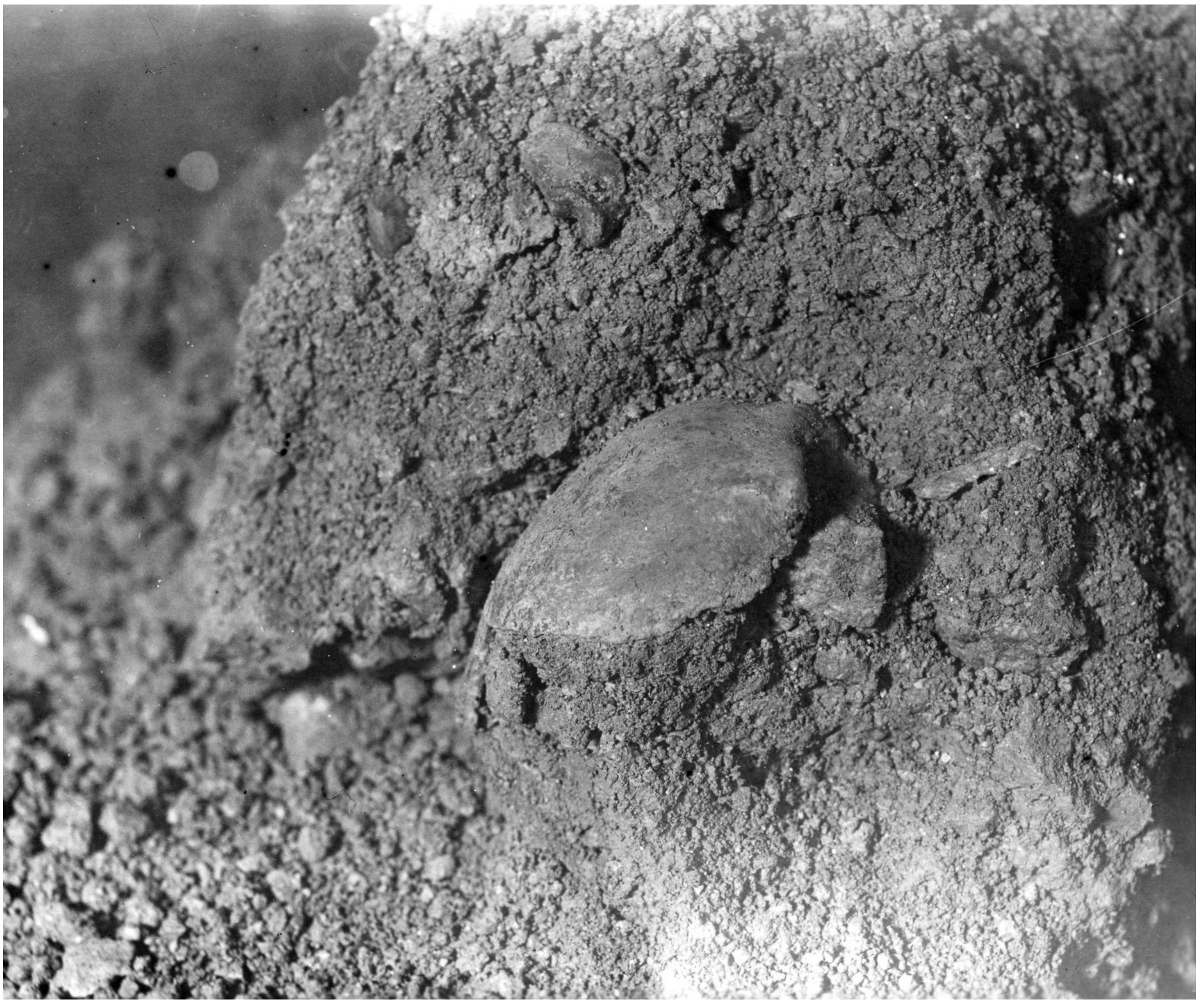

Supplementary Figure 2.

Skeletal remains from Zlatý kůň: skull (Photos: Martin Frouz), vertebrae, costae (Photos: Marek Jantač).

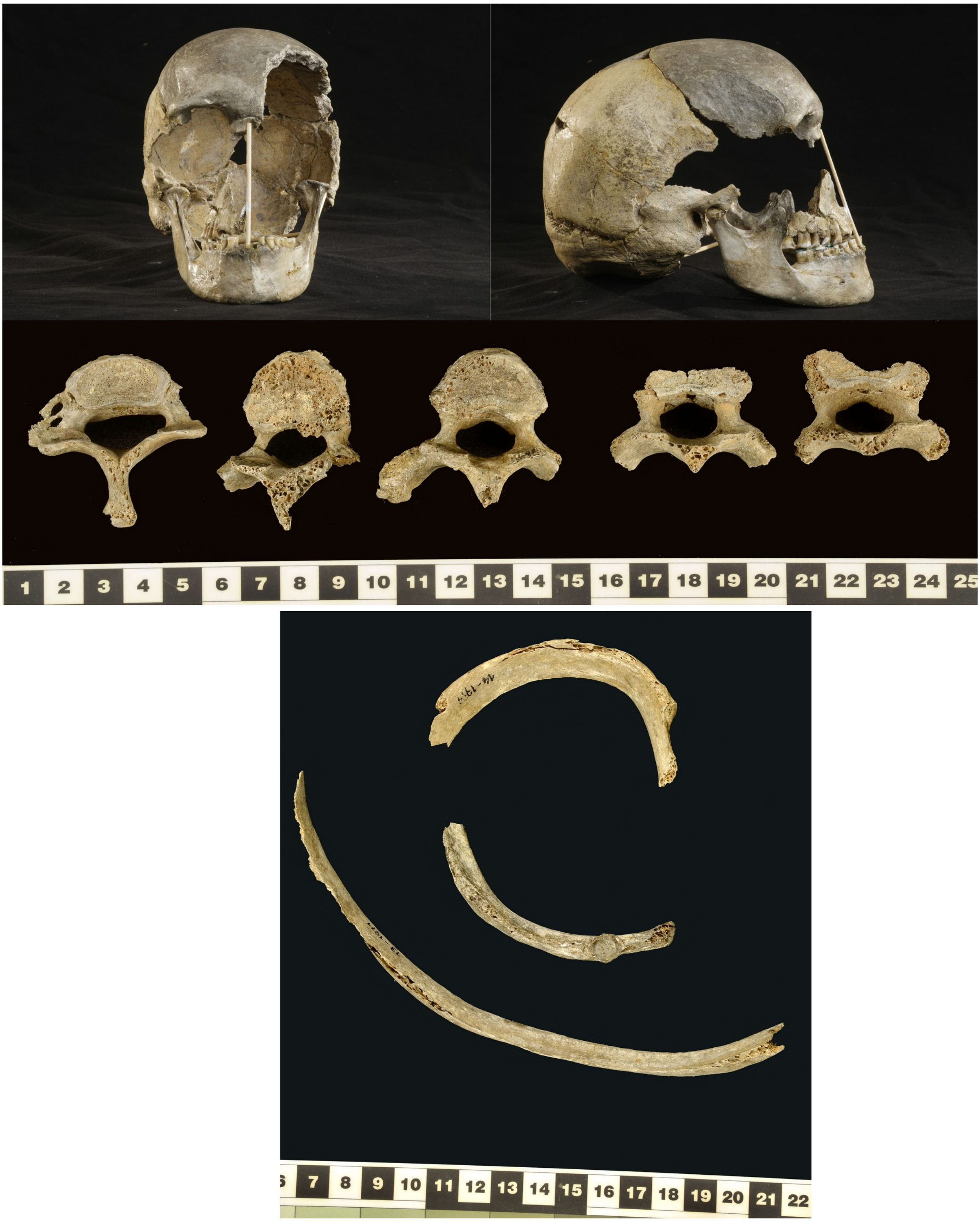

Supplementary Information S2
Radiocarbon Dating and Animal Contamination

Previous Direct Dating
A bone fragment of 4×2 mm in size most likely from the base of the cranium was directly dated in 2002 [18]. This attempt resulted in a radiocarbon date of 12,870±70 BP (GrA-13696), which corresponds in time to the Magdalenian archeological techno-complex described in other sites across Bohemia. A later attempt to date a human rib fragment yielded an estimate of 4,900±70 BP (GrA-23102) and was deemed unreliable because of low carbon content[29]. Dating of a rhinoceros bone from a similar context was unsuccessful because no collagen was preserved[29].

Initial ^{14}C dates of sinter from the Koněprusy Caves, yielded dates of over 21,000 years[30], whereas a later study of calcite coverages on the walls of the Prošek's Dome produced dates of at least 39,800 BP.

New Dating
We performed an additional dating at the Klaus Tschira Archaeometry Center in Mannheim on the detached right zygomatic bone (~1.56 g in total) that showed a perfect refit with the cranium. This date resulted in an age of 23,080±80 BP (MAMS-36077), more than ten thousand radiocarbon years older than the previous date. We therefore suspected the presence of contamination on the specimen and sent the remaining collagen and zygomatic bone fragment for an additional dating at the Oxford Radiocarbon Accelerator Unit (ORAU). The radiocarbon dating was performed using a compound specific approach that consists of isolating and dating the amino acid hydroxyproline (HYP) present in the collagen[31,32]. The new radiocarbon date resulted in an age of 29,650±750 BP (OxA-38022), corresponding to a range of 35,700-32,100 calibrated years before present (cal BP) with 95.4% probability (Supplementary Figure 3).

To further investigate the possible source(s) of contamination, a few micrograms of bone powder and extracted collagen were analyzed by analytical pyrolysis-gas chromatography/mass spectrometry (Py-GC/MS) at the University of Pisa. The analysis of the bone powder revealed the presence of long-chain linear hydrocarbons that can be attributed to a paraffin-based wax as well as 2-ethyl-1-hexanol and its oxidation and

dehydration products. This molecule is widely used as a plasticizer, especially as a phthalate ester[33,34]. These results, which are not consistent with the recorded history of the bone treatment during the post-excavation (see Supplementary section 1), show the difficulty of studying old collections without or with partial conservation records. We also analysed, with the same technique, a fraction of the collagen extracted using the ORAU sample pretreatment comprising a solvent wash, a decalcification in acid, a base wash, a re-acidification and gelatinization (coded 'AG*'), as described in [35]. The Py-GC/MS analysis showed that all the contamination identified in the bone powder had been removed.

Due to the large discrepancy in the three dates (GrA-13696, MAMS-36077 and OxA-38022), and based on the Py-GC/MS results obtained on the bone powder and on the extracted collagen, we used the last 500 mg of bone powder left from the same bone fragment to date again the specimen using the ORAU pretreatment AG* as described above but followed by a step of ultrafiltration (coded AF*)[35,36]. We obtained a fourth date of 15,537±65 BP (OxA-38602). This date is older than the one obtained in Groningen (GrA-13696) but younger than the dates obtained on collagen in Mannheim (MAMS-36077) and on the amino acid hydroxyproline in Oxford (OxA-38022). Here again, the Py-GC/MS analysis of the extracted collagen did not show peaks that could be attributed to the contaminant found in the untreated bone.

It is possible that the bone specimen has been preserved with animal glue. This is a known conservation material for bone[37,38]. A contamination by animal glue is difficult to assess because its main component is collagen (or gelatine) which produces the same prolysis products as the collagen of the bone itself. The glue would have a modern age and its amount may vary across the bone because of the way it was applied and/or the porosity of the bone. This may explain the variability of the ages obtained while the technical data (e.g. C/N ratio) for dates on collagen and on hydroxyproline are all within the acceptable ranges. We further investigate this hypothesis using sequence data in the section "Presence of animal contamination in the extracted DNA", below.

The presence of another contaminant that cannot be characterized by Py-GC/MS cannot be totally ruled out. It is however less likely than the first hypothesis because the contaminant would have to co-elute with the hydroxyproline during the amino acid separation and be totally transparent to the UV-Vis detector of the HPLC system and have a chemical composition with a C/N ratio similar to the one of hydroxyproline.

Overall, the HYP age will be the closest to the real age, because this method is the most efficient in removing contamination, but it could be underestimated for the reasons outlined above. Given we do not have the full conservation record of the specimen (as demonstrated by the pyrolysis) and given the substantial influence of modern contamination on bone samples of Palaeolithic age, the oldest age in this instance, the HYP age, should be most likely considered as a minimum age for the specimen.

Supplementary Figure 3.

Four direct radiocarbon dates calibrated in years before present (calBP) with 68.3% and 95.4% probability. OxA-38022 refers to the HYP dating performed in Oxford. The dates were also calibrated using OxCal 4.4[39] and the IntCal20 calibration curve[40].

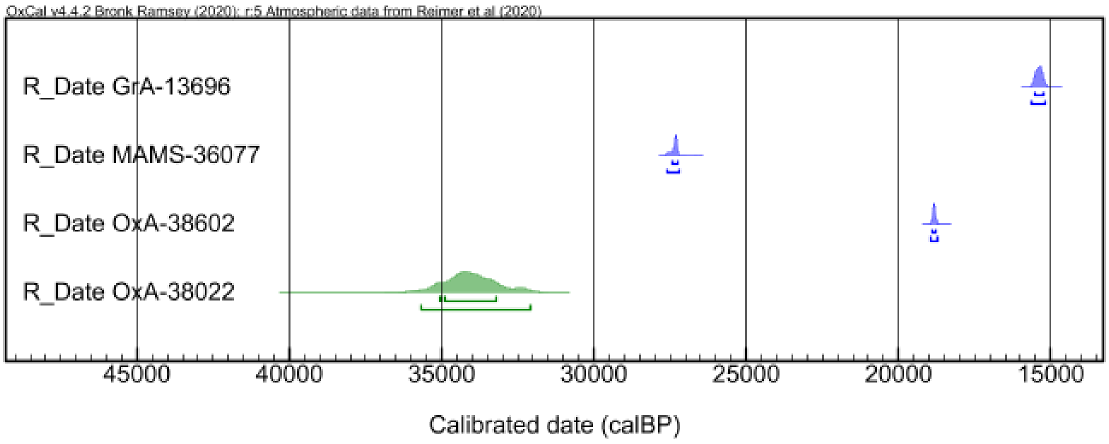

Presence of animal contamination in the extracted DNA

Given that four radiocarbon dating attempts produced inconsistent results, we suspected that a possible presence of recent collagen contamination could be affecting the radiocarbon signal. The putative collagen contamination could derive from treatment of the human remains with consolidants containing animal glue. During radiocarbon dating analysis, contaminants deriving from such material cannot be removed even with compound specific dating (see “Radiocarbon Dating” section, above). This material usually derives from mammalian or fish collagen and, due to its physical, mechanical and chemical properties, it has been regularly used for stabilization and conservation purposes of archeological specimens[37]. We have only sparse information about the post-excavation preservation history of the Zlatý kůň skull, with

no specific evidence of the skull being varnished with animal glue. Moreover, the three more recent radiocarbon dates were performed on a zygomatic bone while genomic results derived from sampling the innermost part of the petrous bone. Nevertheless, we inspected the presence of animal DNA in the retrieved genomic data as a proxy for potential animal collagen contamination that might have affected the entire skull specimen.

First, we conducted a metagenomic investigation of the shotgun data produced from the UDG-half-treated library on two HiSeq 4000 lanes (See Methods section), to investigate the presence of DNA from different animal sources within the specimen. After removing DNA fragments mapping against the human reference sequence (hg19), we used MALT[41] to perform a metagenomic binning against the entire NCBI nucleotide database (1,223,896 entries downloaded in October 2017 from ftp://ftp-trace.ncbi.nih.gov/blast/db/FASTA/nt.gz) using the following parameters: -v -t 112 -m BlastN -at SemiGlobal -top 1 -sup 1 -mq 100 --memoryMode load -ssc -sps. Of 56,621,050 reads assigned by MALT to different taxonomic nodes, we identified 101,178 reads to be specifically assigned to the Bos taurus node, which accounted for the highest proportion of reads assigned to any mammalian taxon (other than H. sapiens) within our database.

Moreover, to deeper explore the possibility of Bos taurus DNA contamination in our dataset we employed a direct mitochondrial genome mapping approach using BWA. For this, we used deep sequence data produced on an entire HiSeq 4000 flow-cell (See Methods section) from the non-UDG treated library. We focused our search not only on cow (Bos taurus), which was previously identified as a possible positive candidate, but also on other mammals which are known to be widely employed for the production of animal glues such as rabbit (Oryctolagus cuniculus) and goat (Capra hircus)[37]. Since mammalian species have a certain level of genetic similarity in conserved regions of the mtDNA, we performed a competitive mapping, for which we compiled a concatenated reference containing the Homo sapiens (NC_012920.1), Capra hircus (NC_005044.2), Bos taurus (NC_006853.1) and Oryctolagus cuniculus (NC_001913.1) mitochondrial genomes in a single fasta file. Subsequently, we used EAGER[42] for mapping DNA fragments longer than 30bp with BWA (parameters: -n 0.01, -l 16500) against the compiled reference, followed by duplicate removal and mapping quality filter set to 30, in order to remove DNA fragments that do not map specifically to one of the four references. The highest mtDNA mean coverage was observed for Homo sapiens (181X) followed by Bos taurus (13X), while Oryctolagus cuniculus and Capra hircus resulted in coverage below 1X (Supplementary Table 1).

Moreover, we extracted the reads mapping to each of the four references and measured the damage pattern with mapDamage2.0[43] and the average fragment length (Supplementary Table 1 and Extended Data Fig. 5a). The typical ancient DNA damage pattern, characterized by the presence of CtoT substitutions increasing towards the 5' end of the molecules, was observed for sequences mapped specifically to the human and cow reference mtDNAs. This excludes the possibility that the isolated DNA mapping specifically to Bos taurus resulted from present-day DNA contamination. Ancient DNA damage is below that observed for the human mtDNA and the retrieved fragment lengths were considerably higher in the reads matching B. taurus (Supplementary Table 1). As such, both characteristics can be regarded as consistent with an age of the isolated cow DNA postdating the unearthing of the Zlatý kůň human remains in the 1950's[44].

Finally, due to the relatively high mean coverage retrieved from the reads mapping to Bos taurus, we were able to use these data from the reconstruction of an almost complete mitochondrial consensus genome. Specifically, we used Geneious to perform a majority call with a minimum coverage of 5X at each position (15,553 positions covered out of 16,338). The resulting sequence was included in a multiple alignment together with a set of 35 present-day Bos taurus and Bos primigenius mtDNA sequences previously compiled by Pereira Verdugo et al. (2019)[45]. Phylogenetic reconstruction was performed in MEGA6[46] using the maximum parsimony algorithm with 97% partial deletion (16,335 considered positions) and 500 bootstrap iterations (Extended Data Fig. 5b). The reconstructed Bos taurus mtDNA branches with haplogroup T3, the most common modern European cattle haplogroup[45].

Taken together, our analysis shows the presence of cow DNA among the DNA retrieved from the petrous portion of the Zlatý kůň skull. This result is consistent with the use of animal glue for the consolidation of this archaeological remains. Such animal glue likely caused contamination of the human collagen used for radiocarbon dating, and could account for the fluctuating and consistently younger dates that hampered an estimation of the specimen's true age.

Supplementary Table 1.

Competitive mapping of Zlatý kůň shotgun sequences to mitochondrial genomes.

Species ID	Reference sequence accession	Mapped reads	Mean fold coverage	Standard deviation coverage	Average fragment length	CtoT damage 5' end
Homo sapiens	NC_012920.1	68377	181.3	36.3	43.94	0.335
Bos taurus	NC_006853.1	3846	13.0	6.9	55.18	0.068
Capra hircus	NC_005044.2	136	0.4	1.8	45.21	n/a
Oryctolagus cuniculus	NC_001913.1	85	0.2	0.9	40.17	n/a

Supplementary Information S3
Mitochondrial Analysis

mtDNA assembly, contamination estimate and haplogroup assignment
The mtDNA capture library was sequenced for ~600,000 paired-end reads and merged sequences were mapped against the mtDNA reference sequence (rCRS) using BWA[47] integrated in EAGER[42]. A total of 42% of merged reads aligned to the rCRS resulting in average mtDNA coverage of 150 fold with a duplication factor of 5X, indicating that we reached the saturation level of this mtDNA enriched library. Damage pattern at both molecule termini was around 14% with an average fragment size of 49bp. The tool schmutzi[48] was used to jointly reconstruct the individual mtDNA consensus sequence and estimate present-day DNA contamination proportions. The latter was estimated to be 4% (3-5%), which can be effectively used as an indirect evidence of low nuclear DNA contamination for libraries with a limited mtDNA/nDNA ratio in shotgun sequencing[49] like the one analyzed here (44X). Three consensus sequences were built increasing the base calling likelihood (form zero to 10) but all resulted in identical sequences with no unassigned positions. The consensus sequence was assigned to haplogoup N using Haplofind[50].

BEAST dating
In order to infer the absolute age of Zlatý kůň we also made use of BEASTv1.8[51] to perform mtDNA tip dating. We used MUSCLE[52] to perform mtDNA multiple genome alignment between 54 present-day mtDNAs, 26 ancient mtDNAs dated between 45,000 and 3,900BP and the reconstructed Zlatý kůň consensus sequence. From the aligned 81 mtDNA sequences we removed all positions represented with a gap or unassigned using MEGA6[46], resulting in a total of 16,433 covered positions. We then identified Tamura-Nei 93 with gamma distributed rate and a fixed fraction of invariable sites as the best-fitting model, using the software ModelGenerator v.85[53]. We selected this model in a program of the BEAST package called *beauti*[54]. Additionally, we provided calibrated dates in years before present for all ancient individuals (Supplementary Table 2) with a uniform prior distribution around the mean date, whereas modern-day mtDNAs were left to zero. The only exception is the Zlatý kůň sequence for which we provided a range between 100,000 and 0 BP, starting from the midpoint 50,000 BP. We selected a strict clock with a fixed mutation rate estimated previously for the entire mtDNA to $2.74 \times 10^{-8}\ site^{-1}\ year^{-1}$ (ref. [55]). A Bayesian skyline coalescent tree

prior was tested with a piecewise linear model and 10 as group number. For this BEAST setting we implemented for two MCMC runs of 50 M states each with sampling every 10K iterations and a 10% burn-in, resulting in 90 M combined states. We obtained a median coalescence age for the macro-haplogroup N, after the divergence of the Oase 1 genome, of 52,000 BP (95% high posterior density [HPD], 48,057-56,350), similar to what previously reported[55]. Moreover, our analysis provides an absolute median age of Zlatý kůň mitogenome of 43,000 BP (95% HPD, 31,505- 52,567). Despite the large high posterior density interval, it only marginally overlaps with the oldest of the four generated radiocarbon dates, providing a much older date as expected from other analyses.

Supplementary Table 2.

Dates in calibrated years before present (BP) for the 26 ancient dated mtDNA used as time anchors in the BEAST analyses.

Sample ID	Date (cal BP)	Upper interval (cal BP)	Lower interval (cal BP)
Ust'Ishim	45045	46880	43210
Fumane2	39805	41110	38500
Oase1	39500	42000	37000
Tianyuan 1301	39475	40120	38830
Kostenki14	37985	38650	37320
GoyetQ116-1	34795	35160	34430
Salkhit1	34425	34950	33900
Mueri1	34035	34360	33710
Cioclovina1	33212	33905	32519
DolniVestonice13	31155	31240	31070
GoyetQ53-1	27975	28230	27720
LaRochette	27592	27784	27400
MA-1	24157	24423	23891
Rigney1	15465	15690	15240
HohleFels79	14670	15070	14270
Oberkassel998	14020	14170	13870
Iboussieres39	11820	12040	11600
LesCloseaux3	9905	10230	9580
HohlensteinStadel	8628	8809	8446
CuiryLesChaudardes1	8205	8360	8050
Loschbour	8054	8181	7927
LaBrana1	7815	7940	7690
Stuttgart	6975	7150	6800
Iceman	5225	5350	5100
Ajvide52	4750	4900	4600
Saqqaq	3885	4170	3600

Phylogenetic tree

To infer the phylogenetic placement of Zlatý kůň mtDNA among other Upper Palaeolithic mtDNA sequences as well as present-day mtDNAs, we built a Maximum Parsimony tree (Supplementary Figure 4). A set of 84 mtDNAs, consisting of 54 worldwide modern-day individuals, 28 published ancient individual older than 24kya, Zlatý kůň and the Vindija 33.25 Neandertal, which was used as outgroup, were aligned using MUSCLE[52]. A maximum parsimony phylogenetic tree was constructed using MEGA6[46] with the Partial-Deletion option and a Site Coverage cutoff of 98%, resulting in 16,528 analyzed alignment positions. The tree robustness was tested with the bootstrap method using 1,000 replicates.

The maximum parsimony tree recapitulates worldwide mtDNA diversity, with the deepest divergent lineages assigned to African individuals and mtDNAs of non-Africans belonging to macro-haplogroups M and N (Supplementary Figure 4). The Zlatý kůň mtDNA falls within the N clade and carries only three additional substitutions (14025C, 16093C, 16172C) and one insertion (16267insC) compared to the ancestral N haplotype. The Upper Palaeolithic Bacho Kiro CC7-335, Bacho Kiro BB7-240 and Slakhit1 mtDNA genomes that also belong to haplogroup N, carry a total of four, four and nine substitutions. As previously described, the mtDNA of the 40ky old Oase1 individual from Romania falls basal to haplogroup N sequences[56]. Other pre-24kya Eurasian mtDNA genomes belong to N-derived mtDNA haplogroups, such as haplogroups R and U, or to macro-haplogroup M (Supplementary Figure 4). Bacho Kiro F6-620 and Bacho Kiro AA7-738 mtDNAs (both haplogroup M) carry the smallest number of substitutions compared to the reconstructed sequence of the modern human ancestor (two substitutions less than Zlatý kůň). Bacho Kiro CC7-2289 mtDNA carries one substitution less than Zlatý kůň. Ust'-Ishim, Bacho Kiro CC7-335 and Bacho Kiro BB7-240 mtDNAs all carry one more substitution than the Zlatý kůň mtDNA. Bootstrap support is low (below 90%) for some of the major clades possibly due to polytomies (Figure 2). However, none of the phylogenetic placements of ancient mtDNA genomes were incongruent with previous results.

Supplementary Figure 4.

Maximum Parsimony tree of 54 modern-day worldwide mtDNA sequences, 28 Palaeolithic Eurasian mtDNAs, Zlatý kůň mtDNA and a Neandertal mtDNA as outgroup. Text color relates to radiocarbon date, i.e. red (undetermined), blue (45-40kya) and yellow (40-24kya). Numbers show the number of substitutions along lineages; letters indicate mtDNA haplogroups.

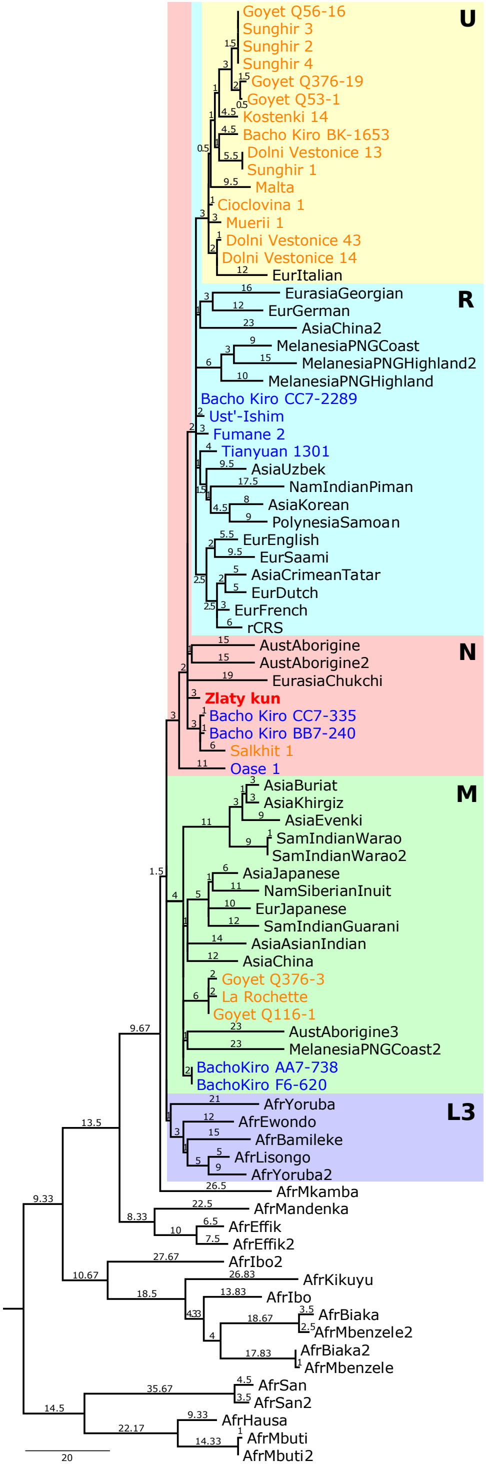

Supplementary Information S4

Phenotype extrapolation, Genetic Sexing and Nuclear Contamination Rates

Phenotypic SNPs

We investigated five SNPs that are associated with phenotypic variation in present-day European populations. Those point mutations are located on the following genes: EDAR, MCM6_LCT, HERC2, SLC45A2 and SLC24A5[57–60]. For all but the last SNP, at least three independent reads were overlapping in the 1240K capture data. Zlatý kůň carried the ancestral variant at all position suggesting that the individual was not able to digest lactose in adulthood, had likely dark skin and dark eyes. One exception is a single read out of four with the derived allele G at position 33951693 of the SLC45A2 gene. Since this variant is not observed in Western Europe before the Neolithic farming expansion we interpret that mutation as likely resulting from a sequencing error or low levels of present-day DNA contamination.

Supplementary Table 3.

Read count of ancestral and derived alleles at five phenotypically relevant SNPs.

Gene	Chromosome	Position	rs	Total Depth 1240K	Ancestral Allele	Count Ancestral	Derived Allele	Count Derived
EDAR	2	109513601	rs3827760	3	A	3	G	0
MCM6_LCT	2	136608646	rs4988235	6	G	6	A	0
SLC45A2	5	33951693	rs16891982	4	C	3	G	1
HERC2	15	28365618	rs12913832	3	A	3	G	0
SLC24A5	15	48426484	rs1426654	0	G	0	A	0

Sex determination

The relative coverage on the captured X- and Y-chromosome SNPs to the captured autosomal SNPs (678,006 total covered SNPs with an average coverage of 1X) indicate that the individual was female (X-rate: 0.778 and Y-rate: 0.045).

For the Zlatý kůň shotgun data, coverage within a track of high mapability ("map35_100": a position is included only if all overlapping 35mers for this position align uniquely back allowing for up to one mismatch; see ref.[61]) was measured separately for all autosomes, X and Y chromosomes based on sequences of a minimum length of 30, a minimum mapping quality of 30 for double-stranded libraries and 25 for single-stranded libraries. Supplementary Figure 5 shows that coverage on the X-chromosome (3.7x) falls within the

range observed over autosomes (3.7-4.0x) whereas the Y-chromosome shows lower coverage than autosomes (~0.08x), again indicating that Zlatý kůň was female. The ratio of Y-chromosome coverage to the range of coverages on X and autosomes divided by two suggests a male contamination rate of 4.1-4.4% in the shotgun data.

Supplementary Figure 5.

Average coverage by shotgun sequences for chromosome X, Y (horizontal dashes) and autosomes (violin plot).

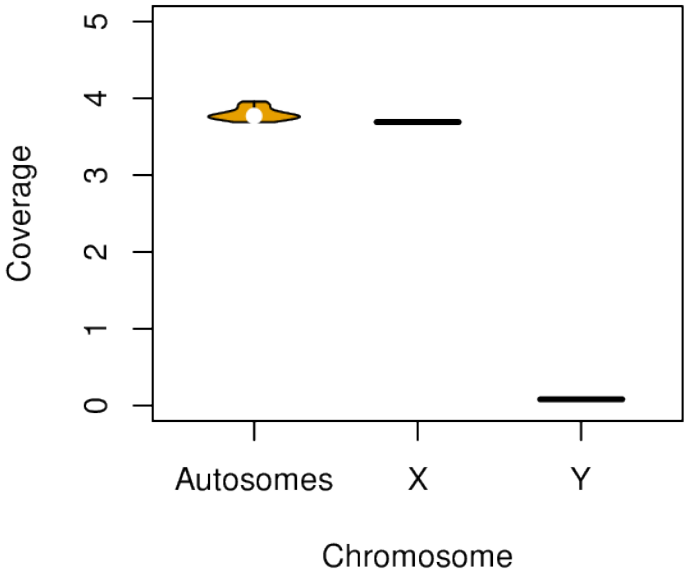

Nuclear contamination estimates based on linkage and damage patterns

We used contamLD, a method that estimates contamination by comparing the linkage between sites when considering only those sequences that contain damage-associated C-to-T changes and are likely uncontaminated to the linkage estimated from all sequences[62].

Double stranded library sequences (capture and shotgun) were considered deaminated if they contained a C-to-T exchange to the human reference at the 5'-most or 3'-most base. After masking two bases at the 5'-end and one base at the 3'-end, sequences with a mapping quality of at least 30 and bases with a quality of at least 30 were considered further for contamination estimates with contamLD.

Single-stranded library sequences were considered deaminated if they contained a C-to-T exchange to the human reference at the 5'-end of the sequence since the data was produced by single-read sequencing and not paired-end. Sequences with a mapping quality of 25 and base quality of 30 were considered further. Only sequences aligning in reverse orientation were considered for sites that were bi-allelic for C and T, and only sequences aligning in forward orientation for G and A sites to reduce the impact of cytosine deamination.

ContamLD was run on the panel of 1240K sites for all datasets. Linkage was determined for the 1000 genomes populations YRI, CEU, JPT and CHB with the panel-data downloaded from https://reichdata.hms.harvard.edu/pub/datasets/release/contamLD/. Supplementary Table 4 shows the estimated contamination.

The contamination level in the single-stranded, non-UDG-treated library was further evaluated using AuthentiCT[63] which uses patterns of ancient DNA damage to infer contamination rates. After filtering for mapping quality of at least 25 and read length of at least 30 bp, the MD field of input bam file was filled using "samtools calmd" command. 10,000 randomly selected reads overlapping with the 1240K sites were used for AuthentiCT estimation. The contamination rate was estimated to be 0.001000±0.020479, consistent with 0.

Supplementary Table 4.

Contamination estimates from contamLD for Zlatý kůň shotgun and 1240k capture data. The table list the damage corrected estimate (DamCorr est.) alongside the standard error (SE). Estimates for the CHB and JPT panels produced the warning "Very_High_Contamination" for all datasets.

	1240K capture data		Shotgun data (double stranded)		Shotgun data (single stranded)	
1000G Pop.	DamCorr est.	SE	DamCorr est.	SE	DamCorr est.	SE
CEU	0.1%	1.0%	1.8%	1.2%	2.2%	0.5%
CHB	-0.6%	0.9%	0.8%	1.3%	2.2%	0.7%
JPT	-0.2%	1.0%	1.0%	1.4%	2.1%	0.7%
YRI	0.4%	0.9%	2.0%	1.2%	3.0%	0.7%

Supplementary Information S5

F3-, F4-statistics and qpGraph analyses of capture data and relationship to modern human populations

F3-outgroup statistics

To investigate the genetic relationship of Zlatý kůň with other previously published hunter-gatherer genomes from Europe we started performing f3-outgroup statistics on the 1240K data in the form f3(Zlatý kůň, X; Mbuti) that effectively measure the genetic affinity between Zlatý kůň and any other European hunter-gatherer compared to a common outgroup represented by the African population Mbuti. We could not identify distinct affinity of the Zlatý kůň genome to any tested individual (Supplementary Figure 6). We therefore built a matrix of dissimilarities with the f3-outgroup statistics and plotted them in the form of a multidimensional scaling (MDS) plot. The MDS plot (Supplementary Figure 7) shows Zlatý kůň falling in close proximity to the two oldest European genomes published so far i.e. Ust'Ishim dated to ~45,000 BP and Oase1 dated to ~40,000 BP[56,64]. Finally, we generated a heat-map matrix of pairwise f3-outgroup statistics showing that Zlatý kůň shares a comparatively small and similar amount of drift with west Eurasian hunter-gatherers, including Ust'Ishim and Oase1 (Supplementary Figure 8).

F4 statistics

To investigate the genetic relationship of Zlatý kůň with other ancient and present-day individuals from Eurasia we performed the following f4 statistic (Mbuti, Zlatý kůň; ancient and present-day Eurasians, X) where X represents the oldest genomes from Eurasia (Ust'Ishim, Kostenki14 and Tianyuan). Almost all statistics are consistent with zero (Supplementary Tables 5, 6 and 7), indicating that Zlatý kůň is equally related to any tested Eurasian individual and suggesting that it represents an ancestry basal to both European and Asian populations.

Significant departures from zero are observed in comparisons of early European farmers or Sardinians to Tianyuan or Kostenki14. In these comparisons Zlaty kun shares significantly more alleles with Tianyuan or Kostenki14 than with the Sardinians or early European farmers. This can be explained by the presence of "Basal Eurasian" ancestry spreading into Eu-

rope with the Early European farmers-related ancestry. The “Basal Eurasian” lineage falls basal to the split between Asians and Europeans, so that Basal Eurasian carriers share more alleles with the Mbuti used as outgroup. The same effect is observed for the statistic f4 (Mbuti, Ust’Ishim; Kostenki14/Tianyuan, Basal Eurasian carriers) << 0 (Supplementary Tables 8, 9).

Moreover, we tested if Zlatý kůň and Ust’Ishim had a differential affinity to other ancient and present-day Eurasian groups via the statistic f4(Mbuti, X; Zlatý kůň, Ust’Ishim). This statistic is significantly positive for most comparisons indicating that Ust’Ishim is genetically closer to ancient and present-day Eurasians than Zlatý kůň is (Supplementary Table 10). The same signal is observed for the f4 statistic (Mbuti, Ust’Ishim; Zlatý kůň, ancient and present-day Eurasians) (Supplementary Table 11).

We note that the Oase1 individual shares less alleles with Zlatý kůň than Tianyuan does (|Z|>2 for f4(Mbuti,Zlatý kůň,Oase1,Tianyuan)). Although not significant, further f4 statistics show the same trend (f4(Mbuti,Zlatý kůň,Oase1,UstIshim), |Z|=1.86; f4(Mbuti, Zlatý kůň, Oase1, Kostenki14), |Z|=1.34; f4(Mbuti,UstIshim,Zlatý kůň,Oase1), |Z|=1.81). Such a signal may be generated from the high Neandertal ancestry of 6-8% in Oase1. To reduce the impact of the high Neandertal ancestry in Oase1, we removed sites that fall within the seven largest Neandertal ancestry regions in Oase1 that were generated most likely by admixture with Neandertals 4-6 generations before the individual lived. This reduced dataset give |Z|<1.1 for the aforementioned four statistics. Note that the statistics f4(Mbuti,AltaiNeandertal,Oase1, {UstIshim,Zlatykun,Tianyuan,Kostenki14}) show substantial sharing of alleles between Oase1 and the Neandertals (|Z|>2.7), suggesting that the filtering is not effective in removing all of the excess of Neandertal ancestry in the Oase1 individual.

We also attempted to infer the phylogenetic placement of Oase1 relative to Ust’-Ishim and Zlatý kůň using the Neandertal-ancestry filtered Oase1 dataset. To the levels of resolution, we are unable to place Oase1 with the available data as the following three statistics are all compatible with zero:

- f4(Mbuti,UstIshim,Zlatý kůň,Oase1_filtered)=-0.000343, Z=-0.326
- f4(Mbuti,Oase1_filtered,UstIshim,Zlatý kůň)=-0.000138, Z=-0.131
- f4(Mbuti,Zlatý kůň,Oase1_filtered,UstIshim)=0.000481, Z=0.466

qpGraph

We aimed to assess the phylogenetic position of Zlatý kůň genome within a previously established admixture graph that includes mainly European hunter-gatherer genomes representing major genomic ancestries[65]. We added Zlatý kůň capture data as a basal lineage diverging after the African population used as outgroup (Mbuti) but before Ust'Ishim. This model provided a good fit, with only two f4 statistics with $3.0<|Z|\leq3.2$, that reveal an un-modeled affinity between Loschbour and MA1 genomes (Supplementary Figure 9). Inverting the branching location of Ust'Ishim and Zlatý kůň does not provide a viable model (maximum $|Z|=5.6$) (Supplementary Figure 10), in line with the previously described f4 statistics. The f4 outliers highlight that some European HGs harbor differential affinity to Asians[65] but the branching of Zlatý kůň before Han and Ust'Ishim support its divergence before Asians and Europeans split (Supplementary Figure 10).

In addition, we built an admixture graph to assess the phylogenetic position of Zlatý kůň compared to both east and west Upper Palaeolithic Eurasian hunter-gatherers representing two main non-African ancestries. Starting from the scaffold (Chimp, (Vindija Neanderthal, Mbuti)), we iteratively added Ust'Ishim, the east Eurasian hunter-gatherer Tianyuan and the west Eurasian hunter-gatherers Sunghir (N=4) to build a skeleton graph. All tested models fit with a single gene flow from Neanderthal into the ancestor of Eurasian hunter-gatherers. Similar to previous publications[66,67], we find a near-trifurcation of Ust'-Ishim, east and west Eurasians; all three possible binary trees involving these three lineages resulted in the same worst Z-score (Supplementary Figure 11). We carried on with the graph where Ust'Ishim is outgroup to Tianyuan and Sunghir, as this graph (Supplementary Figure 11A) gave a better likelihood score (26133.495) compared with the other two topologies (Supplementary Figure 11B and 11C with likelihood scores of 29984.853 and 28106.825, respectively). We finally fitted Zlatý kůň on this skeleton graph, considering it either as a sister lineage of one of the existing nodes, or as resulting from a two-way admixture of two existing lineages. We tested 66 models and the model with Zlatý kůň located basal to all Eurasian hunter-gathereres provided the best fit, with the worst Z-score = -3.042 (Figure 2). The only f4-statistics with $|Z| > 3$ suggested an unexplained affinity between Vindija Neanderthal and Tianyuan. This graph supports Zlatý kůň carrying Neandertal ancestry from a single, common gene flow event into non-Africans (estimated to 5% ancestry) and diverging before Asians and Europeans split from each other.

F4-ratio

We estimated the proportion of Neandertal ancestry on 1240K data using an f4-ratio test[68–71] and the 1240K data as $\frac{f_4(X, Dinka, AltaiNeandertal, Outgroup)}{f_4(VindijaNeandertal, Dinka, AltaiNeandertal, Outgroup)}$. Dinka is assumed to carry no Neandertal ancestry and the chimpanzee genome was used as outgroup. Oase1, which had a Neandertal ancestor within four to six generations before the individual lived [56], shows the highest proportion of Neandertal ancestry (7.3%, block jackknife standard error (SE)=0.9%) among tested individuals. This is followed by the Goyet Q116-1, which is estimated to carry 3.4% (SE=0.5%) Neandertal ancestry. Zlatý kůň falls with 1.8% (SE=0.5%) within the range observed in the other tested individuals (2.8-1.7%).

In an attempt to reduce the affect of ascertainment bias in the 1240K SNP set, we repeated the analyses on a subset of ~277,000 SNPs with known ascertainment on San and Yoruba populations. We note that estimates are higher on average than those of the full dataset. However, the results are broadly consistent with the previous results obtained on the entire 1240K SNP set (Supplementary Table 12). The next section gives Neandertal ancestry estimates based on shotgun data.

Supplementary Figure 6.

f3-statistics based on capture data showing no evidence for a distinct genetic affinity between Zlatý kůň and any European hunter-gatherer. Error bars show one standard error estimated with a weighted block jackknife.

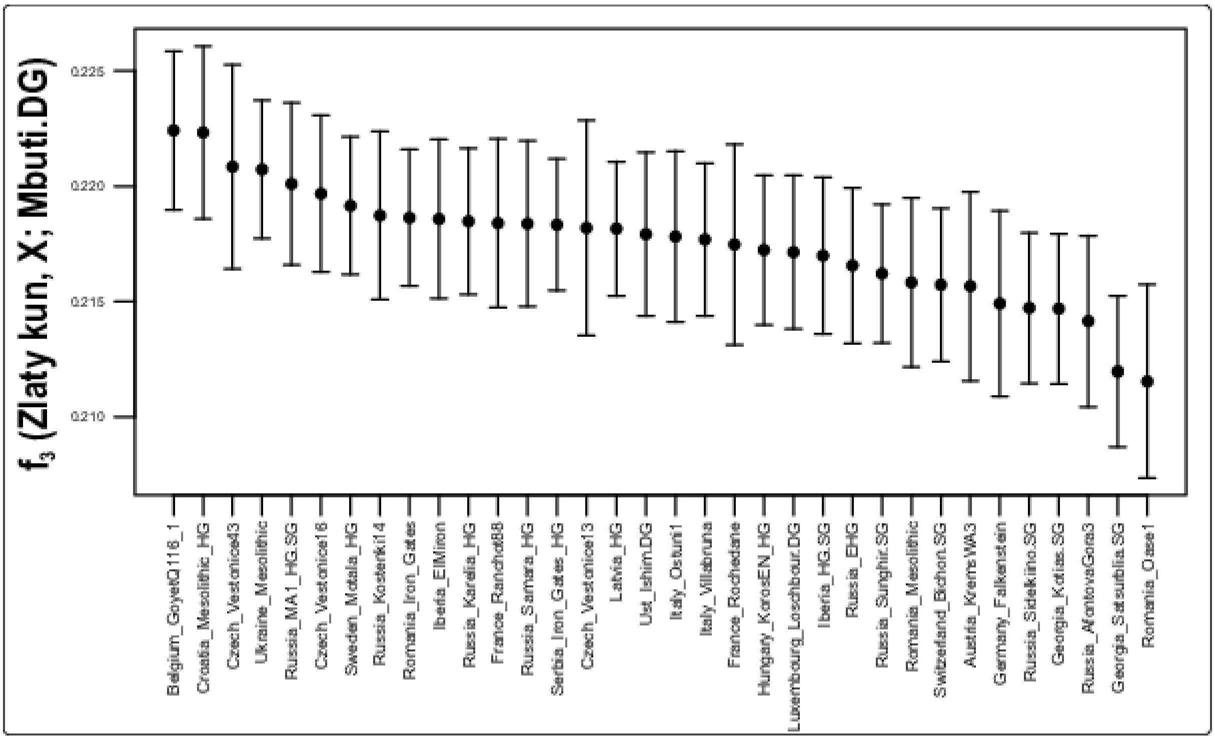

Supplementary Figure 7.

MDS plot of genetic dissimilarities measured as 1-f3outgroup statistics.

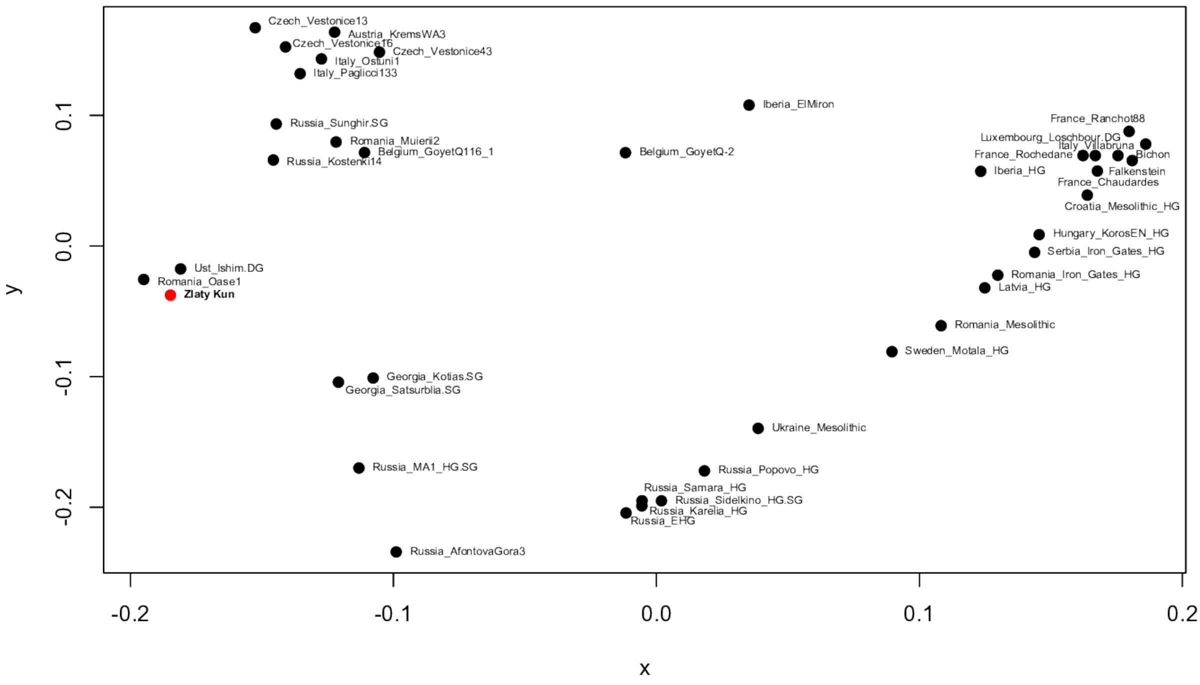

Supplementary Figure 8.

Heat-map matrix of pairwise f3-outgroup statistics with Mbuti as outgroup.

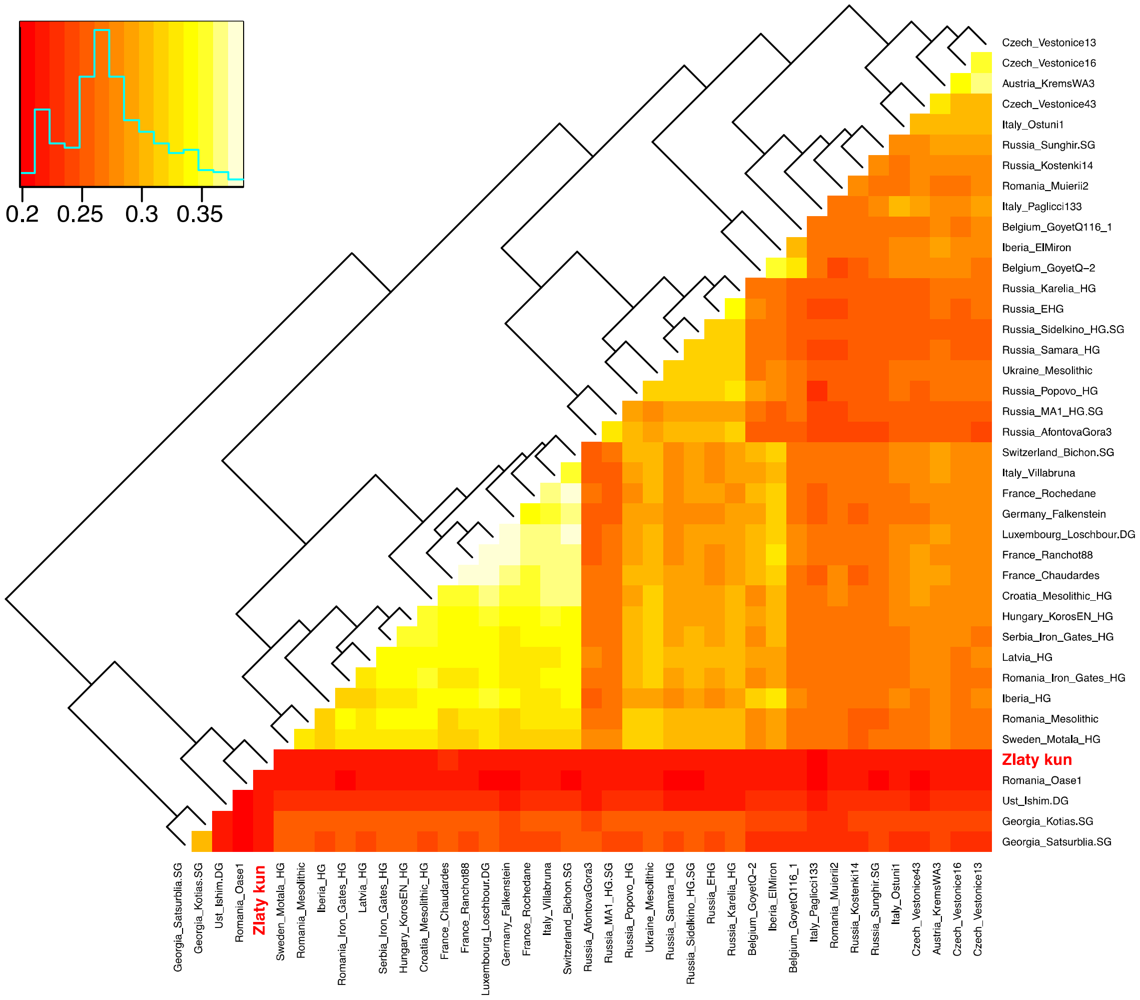

Supplementary Figure 9.
qpGraph with Zlatý kůň falling basal to all other Eurasian hunter-gatherers (worst Z score | 3.2|).

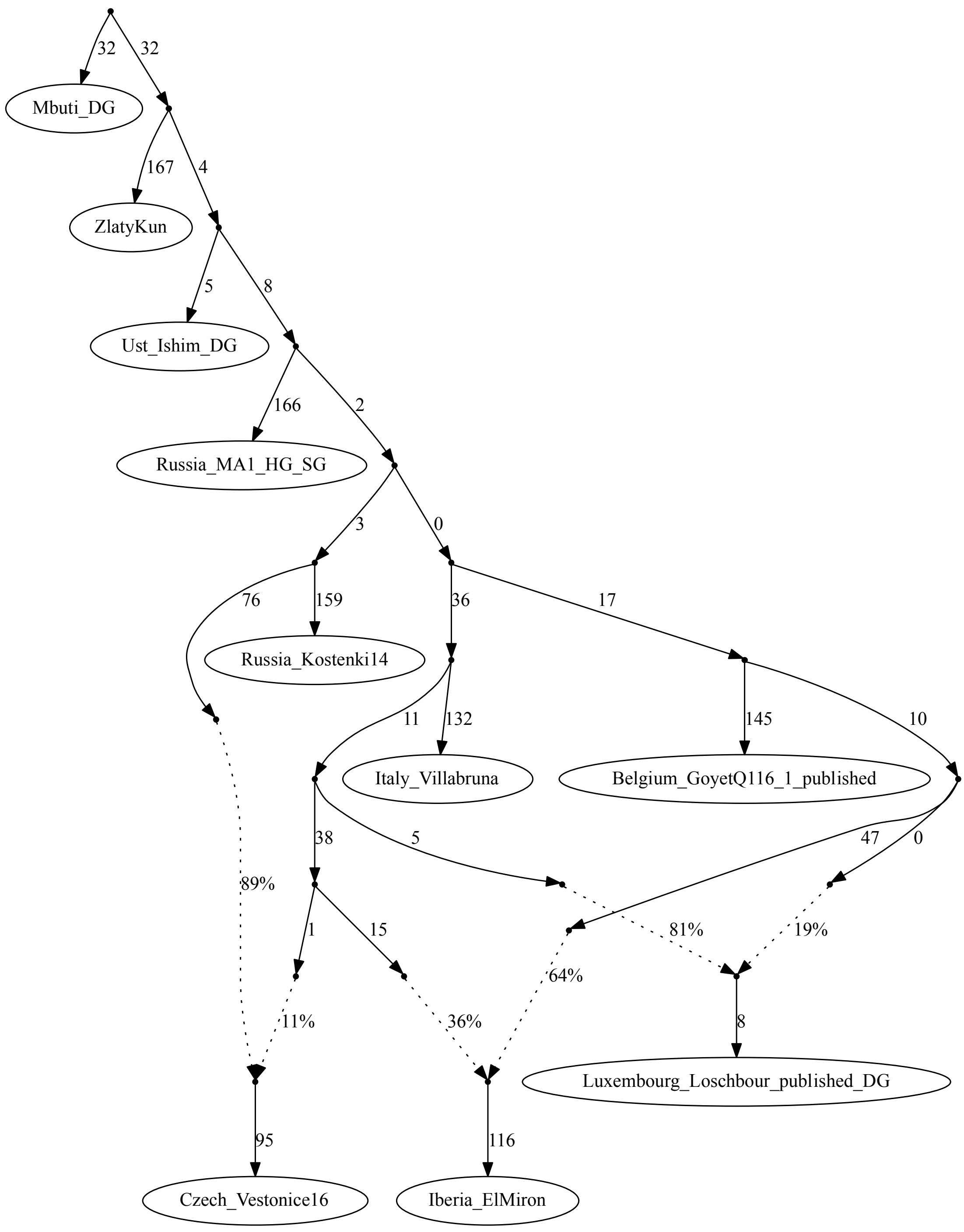

Supplementary Figure 10.
qpGraph with Ust'-Ishim falling basal to all other Eurasian hunter-gatherers (worst Z score=|5.6|).

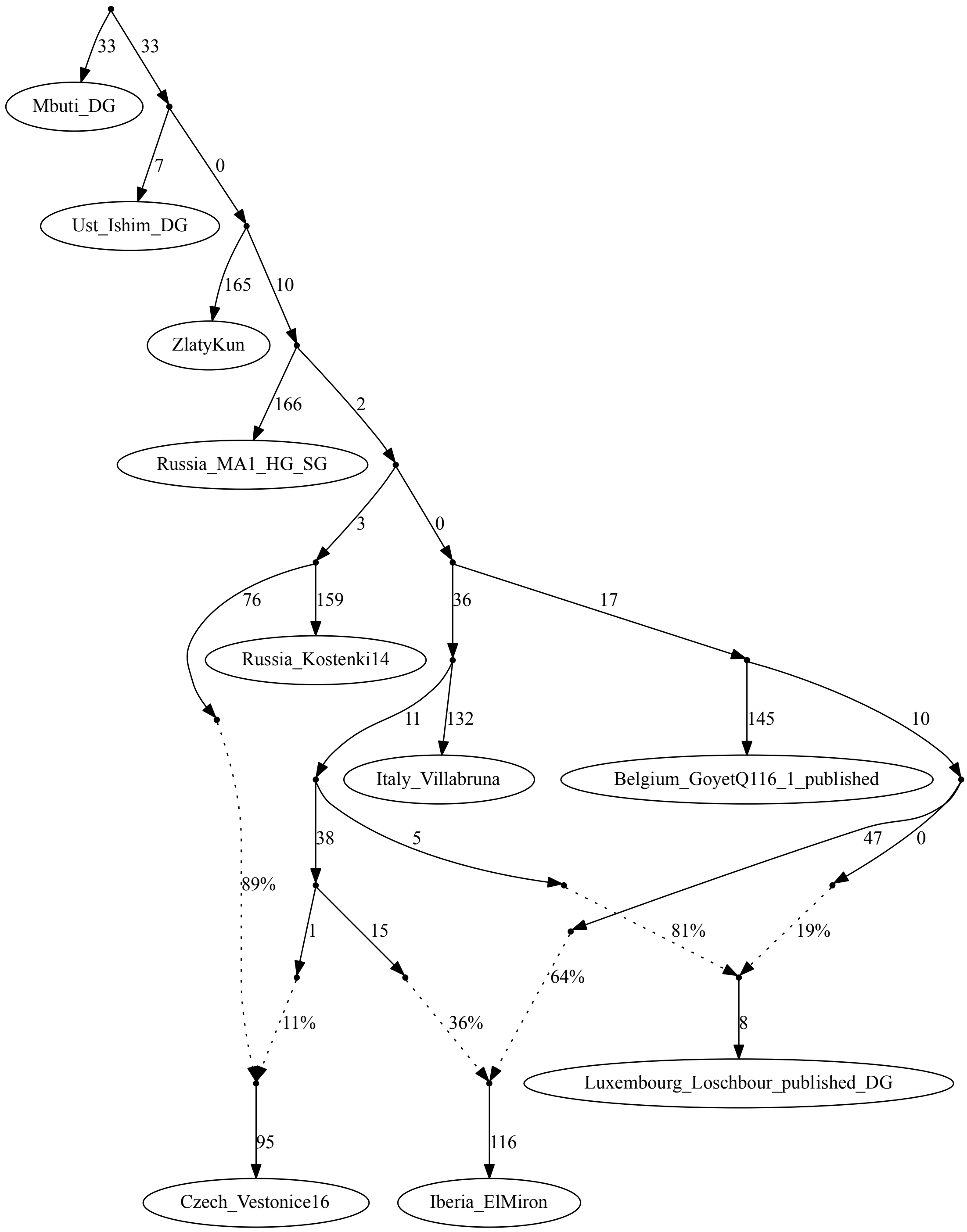

Supplementary Figure 11.

qpGraph model with A) Ust'-Ishim falling basal to all west and east Eurasian hunter-gatherers, B) Ust'-Ishim forming a clade with Sunghir and Tianyuan falling basal, C) Ust'-Ishim forming a clade with Tianyuan and Sunghir falling basal. For all three models the worst Z-score is identical (|Z|=3.0).

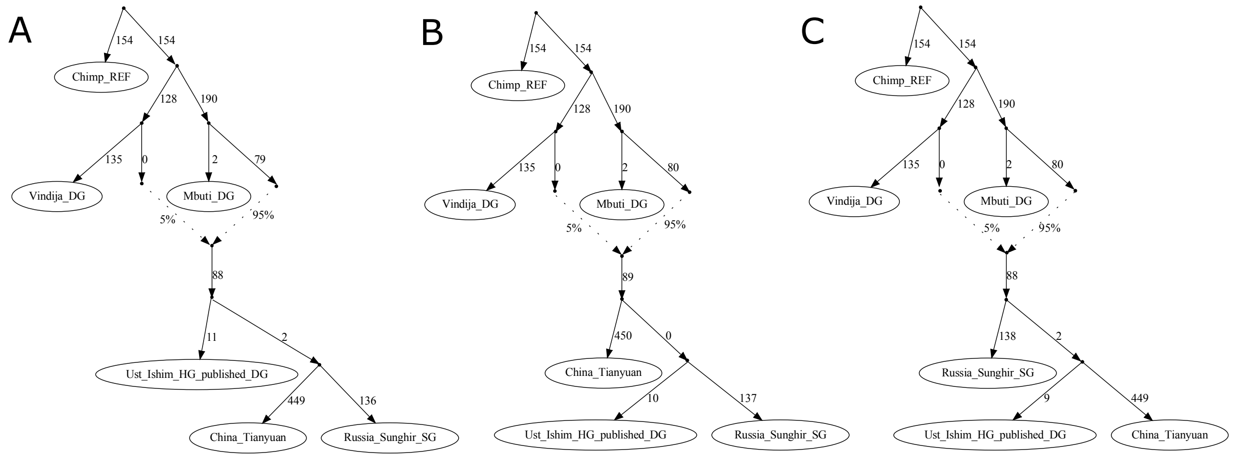

Supplementary Table 5.

f4-statistics of the form f4(Mbuti, ZlatyKun, ancient and present-day Eurasians, Tianyuan) based on capture data.

Outgroup	Pop1	Pop2	Pop3	F4	Zscore	BABA sites	ABBA sites	Tested SNPs
Mbuti.DG	ZlatyKun	Sardinian.DG	China_Tianyuan	0.002189	3.378	30201	29034	532931
Mbuti.DG	ZlatyKun	Germany_LBK_EN	China_Tianyuan	0.002071	2.795	29803	28718	523536
Mbuti.DG	ZlatyKun	Romania_Oase1	China_Tianyuan	0.002824	2.55	7052	6682	130942
Mbuti.DG	ZlatyKun	French.DG	China_Tianyuan	0.001527	2.396	30047	29233	532911
Mbuti.DG	ZlatyKun	Georgia_Satsurblia.SG	China_Tianyuan	0.001291	1.648	20436	19963	366191
Mbuti.DG	ZlatyKun	Han.DG	China_Tianyuan	0.000692	1.171	27829	27460	532929
Mbuti.DG	ZlatyKun	Onge.DG	China_Tianyuan	0.000727	1.106	27602	27221	523438
Mbuti.DG	ZlatyKun	Ust_Ishim_HG	China_Tianyuan	0.000808	0.961	29062	28633	531610
Mbuti.DG	ZlatyKun	Russia_AfontovaGora3	China_Tianyuan	0.000836	0.934	9292	9144	176663
Mbuti.DG	ZlatyKun	Russia_Kostenki14	China_Tianyuan	0.000614	0.737	28094	27781	509314
Mbuti.DG	ZlatyKun	Iberia_ElMiron	China_Tianyuan	0.000509	0.642	21640	21436	399517
Mbuti.DG	ZlatyKun	Luxembourg_Loschbour	China_Tianyuan	0.000485	0.639	28837	28581	526680
Mbuti.DG	ZlatyKun	Dai.DG	China_Tianyuan	0.000321	0.535	27664	27493	532930
Mbuti.DG	ZlatyKun	Italy_Villabruna	China_Tianyuan	0.000368	0.483	27572	27388	500635
Mbuti.DG	ZlatyKun	Czech_Vestonice16	China_Tianyuan	0.000371	0.461	24191	24027	443729
Mbuti.DG	ZlatyKun	Russia_Kostenki14.SG	China_Tianyuan	0.000389	0.459	27526	27331	501404
Mbuti.DG	ZlatyKun	Russia_Karelia_HG	China_Tianyuan	0.000127	0.168	27991	27926	515181
Mbuti.DG	ZlatyKun	Karitiana.DG	China_Tianyuan	0.000051	0.078	28114	28087	532915
Mbuti.DG	ZlatyKun	Belgium_GoyetQ116_1	China_Tianyuan	-0.000316	-0.382	24311	24454	452053
Mbuti.DG	ZlatyKun	Russia_MA1_HG.SG	China_Tianyuan	-0.000449	-0.563	20832	21007	388861

Supplementary Table 6.

f4-statistics of the form f4(Mbuti, ZlatyKun, ancient and present-day Eurasians, Ust'Ishim) based on capture data.

Outgroup	Pop1	Pop2	Pop3	F4	Zscore	BABA sites	ABBA sites	Tested SNPs
Mbuti.DG	ZlatyKun	Sardinian.DG	Ust_Ishim_HG	0.001416	2.124	33497	32658	592621
Mbuti.DG	ZlatyKun	Germany_LBK_EN_Stuttgart.DG	Ust_Ishim_HG	0.001443	1.984	32963	32123	582424
Mbuti.DG	ZlatyKun	Romania_Oase1	Ust_Ishim_HG	0.001948	1.857	7420	7156	135644
Mbuti.DG	ZlatyKun	Georgia_Satsurblia.SG	Ust_Ishim_HG	0.000938	1.221	22786	22403	408590
Mbuti.DG	ZlatyKun	French.DG	Ust_Ishim_HG	0.000752	1.14	33376	32930	592595
Mbuti.DG	ZlatyKun	Russia_AfontovaGora3	Ust_Ishim_HG	0.000198	0.226	9794	9758	183117
Mbuti.DG	ZlatyKun	Han.DG	Ust_Ishim_HG	-0.000045	-0.066	32515	32542	592617
Mbuti.DG	ZlatyKun	Onge.DG	Ust_Ishim_HG	-0.000089	-0.128	31523	31575	580768
Mbuti.DG	ZlatyKun	Luxembourg_Loschbour	Ust_Ishim_HG	-0.000173	-0.22	32164	32266	586022
Mbuti.DG	ZlatyKun	Russia_Kostenki14	Ust_Ishim_HG	-0.000241	-0.281	30915	31051	565536
Mbuti.DG	ZlatyKun	Iberia_ElMiron	Ust_Ishim_HG	-0.000265	-0.339	22472	22582	415691
Mbuti.DG	ZlatyKun	Russia_Kostenki14.SG	Ust_Ishim_HG	-0.000345	-0.407	30167	30358	554578
Mbuti.DG	ZlatyKun	Czech_Vestonice16	Ust_Ishim_HG	-0.000333	-0.42	25097	25252	466596
Mbuti.DG	ZlatyKun	Dai.DG	Ust_Ishim_HG	-0.000381	-0.57	32362	32587	592622
Mbuti.DG	ZlatyKun	Italy_Villabruna	Ust_Ishim_HG	-0.000449	-0.583	29241	29481	534843
Mbuti.DG	ZlatyKun	Russia_Karelia_HG	Ust_Ishim_HG	-0.00055	-0.736	30937	31246	562227
Mbuti.DG	ZlatyKun	Russia_MA1_HG.SG	Ust_Ishim_HG	-0.00062	-0.801	23308	23575	429234
Mbuti.DG	ZlatyKun	Karitiana.DG	Ust_Ishim_HG	-0.000604	-0.844	32374	32732	592606
Mbuti.DG	ZlatyKun	China_Tianyuan	Ust_Ishim_HG	-0.000808	-0.961	28633	29062	531610
Mbuti.DG	ZlatyKun	Belgium_GoyetQ116_1	Ust_Ishim_HG	-0.001186	-1.492	25876	26448	482131

Supplementary Table 7.

f4-statistics of the form f4(Mbuti, ZlatyKun, ancient and present-day Eurasians, Kostenki14) based on capture data.

Outgroup	Pop1	Pop2	Pop3	F4	Zscore	BABA sites	ABBA sites	Tested SNPs
Mbuti.DG	ZlatyKun	Sardinian.DG	Russia_Kostenki14	0.001692	2.743	31046	30087	566875
Mbuti.DG	ZlatyKun	Germany_LBK_EN_Stuttgart.DG	Russia_Kostenki14	0.001757	2.508	30686	29707	557064
Mbuti.DG	ZlatyKun	French.DG	Russia_Kostenki14	0.000998	1.628	30966	30401	566847
Mbuti.DG	ZlatyKun	Romania_Oase1	Russia_Kostenki14	0.001467	1.342	6829	6645	125602
Mbuti.DG	ZlatyKun	Georgia_Satsurblia.SG	Russia_Kostenki14	0.000826	1.07	21189	20867	390253
Mbuti.DG	ZlatyKun	Russia_AfontovaGora3	Russia_Kostenki14	0.000524	0.571	8838	8748	170403
Mbuti.DG	ZlatyKun	Iberia_ElMiron	Russia_Kostenki14	0.000324	0.438	20225	20097	393690
Mbuti.DG	ZlatyKun	Han.DG	Russia_Kostenki14	0.000247	0.358	31599	31459	566870
Mbuti.DG	ZlatyKun	Ust_Ishim_HG	Russia_Kostenki14	0.000241	0.281	31051	30915	565536
Mbuti.DG	ZlatyKun	Russia_Kostenki14.SG	Russia_Kostenki14	0.00006	0.239	13532	13500	530641
Mbuti.DG	ZlatyKun	Onge.DG	Russia_Kostenki14	0.000147	0.21	30673	30592	555654
Mbuti.DG	ZlatyKun	Luxembourg_Loschbour	Russia_Kostenki14	0.000044	0.061	29238	29213	560458
Mbuti.DG	ZlatyKun	Dai.DG	Russia_Kostenki14	-0.000101	-0.148	31478	31535	566875
Mbuti.DG	ZlatyKun	Italy_Villabruna	Russia_Kostenki14	-0.000168	-0.229	26707	26793	512522
Mbuti.DG	ZlatyKun	Czech_Vestonice16	Russia_Kostenki14	-0.000179	-0.236	22388	22468	444591
Mbuti.DG	ZlatyKun	Russia_Karelia_HG	Russia_Kostenki14	-0.000241	-0.347	28441	28571	538584
Mbuti.DG	ZlatyKun	Karitiana.DG	Russia_Kostenki14	-0.000286	-0.41	30865	31028	566861
Mbuti.DG	ZlatyKun	Russia_MA1_HG.SG	Russia_Kostenki14	-0.000428	-0.561	21447	21623	410676
Mbuti.DG	ZlatyKun	China_Tianyuan	Russia_Kostenki14	-0.000614	-0.737	27781	28094	509314
Mbuti.DG	ZlatyKun	Belgium_GoyetQ116_1	Russia_Kostenki14	-0.000833	-1.071	23684	24068	460480

Supplementary Table 8.

f4-statistics showing that present-day Europeans, Early European farmers and Caucasian hunter-gatherers carry Basal Eurasian ancestry.

Outgroup	Pop1	Pop2	Pop3	F4	Zscore	BABA sites	ABBA sites	Tested SNPs
Mbuti.DG	Ust_Ishim_HG	China_Tianyuan	Romania_Oase1	-0.004703	-5.058	9321	10177	182015
Mbuti.DG	Ust_Ishim_HG	China_Tianyuan	Sardinian.DG	-0.002593	-4.202	48401	50690	882979
Mbuti.DG	Ust_Ishim_HG	China_Tianyuan	Germany_LBK_EN_Stuttgart	-0.002589	-3.74	47829	50076	867718
Mbuti.DG	Ust_Ishim_HG	China_Tianyuan	French.DG	-0.002063	-3.42	48676	50497	882947
Mbuti.DG	Ust_Ishim_HG	China_Tianyuan	Georgia_Satsurblia.SG	-0.00236	-3.262	33211	34652	610299
Mbuti.DG	Ust_Ishim_HG	China_Tianyuan	Russia_Karelia_HG	-0.001386	-2.039	45120	46279	836823
Mbuti.DG	Ust_Ishim_HG	China_Tianyuan	Russia_AfontovaGora3	-0.000954	-1.234	13009	13245	247645
Mbuti.DG	Ust_Ishim_HG	China_Tianyuan	Italy_Villabruna	-0.000136	-0.192	43108	43214	781862
Mbuti.DG	Ust_Ishim_HG	China_Tianyuan	Luxembourg_Loschbour	0.000141	0.198	48369	48246	873611
Mbuti.DG	Ust_Ishim_HG	China_Tianyuan	Russia_Kostenki14	0.000315	0.434	47531	47262	851894
Mbuti.DG	Ust_Ishim_HG	China_Tianyuan	Iberia_ElMiron	0.000366	0.485	31855	31642	582196
Mbuti.DG	Ust_Ishim_HG	China_Tianyuan	Han.DG	0.000295	0.522	46645	46384	882975
Mbuti.DG	Ust_Ishim_HG	China_Tianyuan	Dai.DG	0.000328	0.58	46536	46247	882975
Mbuti.DG	Ust_Ishim_HG	China_Tianyuan	Czech_Vestonice16	0.000482	0.636	36758	36436	666521
Mbuti.DG	Ust_Ishim_HG	China_Tianyuan	Karitiana.DG	0.000406	0.674	47409	47050	882953
Mbuti.DG	Ust_Ishim_HG	China_Tianyuan	Onge.DG	0.000508	0.848	46347	45907	866765
Mbuti.DG	Ust_Ishim_HG	China_Tianyuan	Russia_MA1_HG.SG	0.000833	1.145	35005	34475	635585
Mbuti.DG	Ust_Ishim_HG	China_Tianyuan	Belgium_GoyetQ116_1	0.001311	1.676	37640	36750	679048

Supplementary Table 9.

f4-statistics showing that present-day Europeans, Early European farmers and Caucasian hunter-gatherers carry Basal Eurasian ancestry.

Outgroup	Pop1	Pop2	Pop3	F4	Zscore	BABA sites	ABBA sites	Tested SNPs
Mbuti.DG	Ust_Ishim_HG	Russia_Kostenki14	Sardinian.DG	-0.003039	-5.285	56818	60057	1065924
Mbuti.DG	Ust_Ishim_HG	Russia_Kostenki14	Germany_LBK_EN_Stuttgart	-0.00318	-4.902	56006	59339	1048117
Mbuti.DG	Ust_Ishim_HG	Russia_Kostenki14	French.DG	-0.002551	-4.541	57293	60012	1065872
Mbuti.DG	Ust_Ishim_HG	Russia_Kostenki14	Georgia_Satsurblia.SG	-0.003019	-4.351	39630	41868	741201
Mbuti.DG	Ust_Ishim_HG	Russia_Kostenki14	Romania_Oase1	-0.004275	-4.317	9589	10370	182588
Mbuti.DG	Ust_Ishim_HG	Russia_Kostenki14	Russia_Karelia_HG	-0.001748	-2.73	50683	52366	962399
Mbuti.DG	Ust_Ishim_HG	Russia_Kostenki14	Russia_AfontovaGora3	-0.001439	-1.908	12844	13203	249463
Mbuti.DG	Ust_Ishim_HG	Russia_Kostenki14	Italy_Villabruna	-0.000538	-0.83	44531	44988	849963
Mbuti.DG	Ust_Ishim_HG	Russia_Kostenki14	Luxembourg_Loschbour	-0.000389	-0.595	55684	56094	1055635
Mbuti.DG	Ust_Ishim_HG	Russia_Kostenki14	China_Tianyuan	-0.000315	-0.434	47262	47531	851894
Mbuti.DG	Ust_Ishim_HG	Russia_Kostenki14	Dai.DG	-0.000096	-0.153	60328	60430	1065923
Mbuti.DG	Ust_Ishim_HG	Russia_Kostenki14	Han.DG	-0.000088	-0.142	60469	60562	1065915
Mbuti.DG	Ust_Ishim_HG	Russia_Kostenki14	Karitiana.DG	-0.000008	-0.013	59498	59507	1065898
Mbuti.DG	Ust_Ishim_HG	Russia_Kostenki14	Iberia_ElMiron	0.000074	0.106	30864	30820	597922
Mbuti.DG	Ust_Ishim_HG	Russia_Kostenki14	Onge.DG	0.000206	0.307	58753	58538	1041938
Mbuti.DG	Ust_Ishim_HG	Russia_Kostenki14	Czech_Vestonice16	0.000229	0.329	35846	35685	701758
Mbuti.DG	Ust_Ishim_HG	Russia_Kostenki14	Russia_MA1_HG.SG	0.000264	0.393	40523	40324	753667
Mbuti.DG	Ust_Ishim_HG	Russia_Kostenki14	Belgium_GoyetQ116_1	0.001268	1.772	39153	38223	733860

Supplementary Table 10.

f4-statistics showing that Ust'Ishim is more closely related to ancient and present-day Eurasians populations than Zlatý kůň.

Outgroup	Pop1	Pop2	Pop3	F4	Zscore	BABA sites	ABBA sites	Tested SNPs
Mbuti.DG	Han.DG	ZlatyKun	Ust_Ishim_HG_	0.003029	4.931	37743	35768	652052
Mbuti.DG	Dai.DG	ZlatyKun	Ust_Ishim_HG_	0.002701	4.397	37612	35850	652056
Mbuti.DG	Onge.DG	ZlatyKun	Ust_Ishim_HG_	0.002834	4.351	36532	34721	638832
Mbuti.DG	Karitiana.DG	ZlatyKun	Ust_Ishim_HG_	0.002502	3.776	37626	35995	652039
Mbuti.DG	Czech_Vestonice16	ZlatyKun	Ust_Ishim_HG_	0.002919	3.701	28808	27336	504148
Mbuti.DG	Belgium_GoyetQ116_1	ZlatyKun	Ust_Ishim_HG_	0.003103	3.597	30289	28669	522295
Mbuti.DG	Luxembourg_Loschbour_	ZlatyKun	Ust_Ishim_HG_	0.002684	3.532	37182	35451	644920
Mbuti.DG	Russia_MA1_HG.SG	ZlatyKun	Ust_Ishim_HG_	0.002733	3.524	27223	25935	471090
Mbuti.DG	Russia_Kostenki14	ZlatyKun	Ust_Ishim_HG_	0.002637	3.206	35903	34260	623208
Mbuti.DG	Iberia_ElMiron	ZlatyKun	Ust_Ishim_HG_	0.002427	3.081	25385	24300	447064
Mbuti.DG	Italy_Villabruna	ZlatyKun	Ust_Ishim_HG_	0.002253	3.013	33317	32005	582100
Mbuti.DG	Russia_Kostenki14.SG	ZlatyKun	Ust_Ishim_HG_	0.0024	2.88	34856	33394	609197
Mbuti.DG	Sardinian.DG	ZlatyKun	Ust_Ishim_HG_	0.001579	2.77	36875	35845	652056
Mbuti.DG	Russia_AfontovaGora3	ZlatyKun	Ust_Ishim_HG_	0.002231	2.689	10824	10388	195694
Mbuti.DG	French.DG	ZlatyKun	Ust_Ishim_HG_	0.00144	2.532	37135	36196	652028
Mbuti.DG	China_Tianyuan	ZlatyKun	Ust_Ishim_HG_	0.001987	2.401	32803	31652	579104
Mbuti.DG	Germany_LBK_EN_Stuttgart	ZlatyKun	Ust_Ishim_HG_	0.001442	2.142	36254	35330	640919
Mbuti.DG	Georgia_Satsurblia.SG	ZlatyKun	Ust_Ishim_HG_	0.001142	1.585	25163	24649	450114
Mbuti.DG	Russia_Karelia_HG	ZlatyKun	Ust_Ishim_HG_	0.0011	1.538	34792	34115	615806
Mbuti.DG	Romania_Oase1	ZlatyKun	Ust_Ishim_HG_	-0.000127	-0.126	7657	7675	144939

Supplementary Table 11.

f4-statistics showing that all European HGs and present-day East Eurasians share higher affinities to Ust'Ishim than Zlatý kůň does.

Outgroup	Pop1	Pop2	Pop3	F4	Zscore	BABA sites	ABBA sites	Tested SNPs
Mbuti.DG	Ust_Ishim_HG	ZlatyKun	Romania_Oase1	-0.001878	-1.808	7657	7929	144939
Mbuti.DG	Ust_Ishim_HG	ZlatyKun	Sardinian.DG	-0.000211	-0.304	36875	37012	652056
Mbuti.DG	Ust_Ishim_HG	ZlatyKun	Georgia_Satsurblia.SG	-0.000194	-0.24	25163	25251	450114
Mbuti.DG	Ust_Ishim_HG	ZlatyKun	Germany_LBK_EN_Stuttgart	-0.000163	-0.213	36254	36359	640919
Mbuti.DG	Ust_Ishim_HG	ZlatyKun	French.DG	0.000377	0.536	37135	36889	652028
Mbuti.DG	Ust_Ishim_HG	ZlatyKun	Russia_AfontovaGora3	0.001412	1.617	10824	10548	195694
Mbuti.DG	Ust_Ishim_HG	ZlatyKun	Russia_Karelia_HG	0.001224	1.63	34792	34039	615806
Mbuti.DG	Ust_Ishim_HG	ZlatyKun	Italy_Villabruna	0.002322	3.061	33317	31965	582100
Mbuti.DG	Ust_Ishim_HG	ZlatyKun	Russia_Kostenki14.SG	0.002597	3.109	34856	33274	609197
Mbuti.DG	Ust_Ishim_HG	ZlatyKun	Luxembourg_Loschbour	0.002499	3.192	37182	35570	644920
Mbuti.DG	Ust_Ishim_HG	ZlatyKun	China_Tianyuan	0.002713	3.22	32803	31232	579104
Mbuti.DG	Ust_Ishim_HG	ZlatyKun	Russia_Kostenki14	0.002708	3.251	35903	34216	623208
Mbuti.DG	Ust_Ishim_HG	ZlatyKun	Iberia_ElMiron	0.002703	3.305	25385	24177	447064
Mbuti.DG	Ust_Ishim_HG	ZlatyKun	Onge.DG	0.002777	3.684	36532	34758	638832
Mbuti.DG	Ust_Ishim_HG	ZlatyKun	Han.DG	0.002789	3.79	37743	35924	652052
Mbuti.DG	Ust_Ishim_HG	ZlatyKun	Dai.DG	0.002877	3.847	37612	35735	652056
Mbuti.DG	Ust_Ishim_HG	ZlatyKun	Russia_MA1_HG.SG	0.0032	3.903	27223	25715	471090
Mbuti.DG	Ust_Ishim_HG	ZlatyKun	Czech_Vestonice16	0.00325	3.914	28808	27170	504148
Mbuti.DG	Ust_Ishim_HG	ZlatyKun	Karitiana.DG	0.002864	3.919	37626	35758	652039
Mbuti.DG	Ust_Ishim_HG	ZlatyKun	Belgium_GoyetQ116_1	0.004097	4.729	30289	28149	522295

Supplementary Table 12.

Neandertal ancestry estimates according to an f4 ratio test based on SNP capture data.

Individual	Nea % on 1240K SNPs	SE	Zscore	Nea % on 277K SNPs	SE	Zscore
Belgium_GoyetQ116_1_published	0.033548	0.004744	7,072	0.037877	0.00701	5.404
China_Tianyuan	0.020738	0.004593	4,515	0.023483	0.006857	3.425
Czech_Vestonice16	0.020616	0.004668	4,417	0.024357	0.006605	3.687
France_Ranchot88_published	0.024586	0.005238	4,694	0.027179	0.009422	2.885
Georgia_Kotias.SG	0.017662	0.003493	5,057	0.021217	0.006269	3.384
Germany_LBK_EN_Stuttgart.DG	0.02025	0.003378	5,994	0.023891	0.005339	4.475
Iberia_ElMiron	0.028014	0.004925	5,688	0.032654	0.007161	4.56
Italy_Villabruna	0.024983	0.004365	5,723	0.027957	0.006722	4.159
Luxembourg_Loschbour.DG	0.021482	0.003783	5,678	0.02663	0.005515	4.828
Romania_Oase1	0.073117	0.008750	8,357	0.077316	0.012656	6.109
Russia_AfontovaGora3	0.020994	0.006012	3,492	0.023433	0.010562	2.219
Russia_Karelia_HG	0.022673	0.003726	6,086	0.024554	0.006216	3.95
Russia_Kostenki14	0.018935	0.004183	4,526	0.022006	0.006406	3.435
Russia_MA1_HG.SG	0.023659	0.004507	5,249	0.024601	0.007299	3.371
Russia_Sunghir1.SG	0.02286	0.004570	5,003	0.024784	0.00705	3.515
Russia_Sunghir2.SG	0.017746	0.004280	4,139	0.019081	0.006409	2.977
Russia_Sunghir3.SG	0.022242	0.004390	5,066	0.032323	0.006167	5.241
Russia_Sunghir4.SG	0.025086	0.004459	5,626	0.035623	0.006182	5.763
Switzerland_Bichon.SG	0.020666	0.003934	5,253	0.01968	0.006203	3.173
Ust_Ishim.DG	0.022104	0.004097	5,395	0.035056	0.005738	6.109
ZlatyKun	0.018008	0.005300	3,398	0.026535	0.007831	3.388

Supplementary Information S6

Neandertal ancestry estimates and relationship to late Neandertals from shotgun data

Neandertal ancestry

We use the high-coverage Vindija and Altai Neandertal genomes and the genome of an Mbuti from Africa (HGDP00982[61,72]) to estimate the percentage of Zlatý kůň's genome that derives from Neandertals. We apply recommended filters to the Vindija and Altai Neandertal high-coverage genotypes[69] and to the Mbuti genotypes[61]. A random allele was sampled at heterozygous sites. The ape ancestor was inferred from the UCSC genome browser processed lastz[73] whole genome alignments of the chimpanzee, bonobo, gorilla, orangutan and rhesus macaque genomes (versions: pantro4, panpan2.1, gorgor3, ponabe2, rhemac2), requiring all 5 genomes to be covered and show an identical base. Alternatively, we used the Denisova genotypes with default filtering as an outgroup.

A random sequence of a minimum mapping quality of 30 containing a base of minimum base quality of 30 was sampled from the shotgun data of Zlatý kůň for each site. We separately sampled random sequences from Ust'Ishim, Loschbour, Kostenki14 and Sughir1-4 [64,74–76].

Following previous approaches, ancestry was estimated from a ratio of D-statistics numerators: $\frac{S(X,Mbuti,Altai,ApeAncestor)}{S(Vindija,Mbuti,Altai,ApeAncestor)}$. A 5Mb block jackknife standard error was estimated for each S-statistics individually. With S_1, S_2 the two S-statistics and S_1err, S_2err the corresponding standard error estimates, the standard error of the ratio was estimated as

$\frac{S_1}{S_2} \times \sqrt{\left(\frac{S_1err}{S_1}\right)^2 + \left(\frac{S_2err}{S_2}\right)^2}$. To eliminate an influence from cytosine deamination all statistics were calculated only for sites that showed a transversion between the inferred ape-ancestor and the Altai Neandertal genome. Supplementary Table 13 summarizes the results. Zlatý kůň shows the highest estimate (3.2% ±0.32%) among all tested genomes. However, Zlatý kůň's estimate is only significantly higher than that for Loschbour and that for Sunghir1 at a significance level of two standard errors.

To ensure that differences in Neandertal ancestry are not driven by a difference of sites that are covered in each of the tested hunter gatherer genomes, we repeated the analysis by estimating the Neandertal ancestry percentage only for those sites that are covered in Zlatý

kůň and a comparison genome. The results of all pairwise comparisons are shown in Supplementary Table 14.

The inferred ape ancestor's sequence is more distantly related to the remaining compared sequences in the S-statistics than the degree to which any of these sequences are related to each other. The large distance to the ape ancestor could bias results when error rates differ substantially among the compared sequences. To test this, we recalculated S-statistics for pairwise overlapping sites using the Denisovan genome as an additional outgroup, requiring that the state in the Denisovan genome matches that in the inferred ape ancestor and filtering again for transversions (Supplementary Table 15).

Relationship to late Neandertals

Sequenced early hunter-gatherers in Europe and Asia lived potentially close in time to late Neandertals. To test whether Zlatý kůň differs from other early hunter gatherers in her relationship to late Neandertals, we calculated D-statistics of the form D(Late Neandertal, Late Neandertal, Early Hunter Gatherer, Outgroup), where the Outgroup is either the ape ancestor, Denisova or Mbuti. The ape ancestor, Denisova. Mbuti and early hunter gatherer input was produced as described in the previous section. Following previous approaches[77], late Neandertals were added by restricting the analysis to sequences with evidence of deamination and randomly sampling a single sequence of at least 35bp length with a minimum mapping quality of 25 and containing a base of minimum base quality of 30 per site. Supplementary Tables 16-19 show the results. Zlatý kůň's relationship with late Neandertals is qualitatively similar to that of other early hunter gatherers.

Pairwise comparisons of Zlatý kůň to hunter-gatherer genomes in their sharing of alleles with archaic genomes showed differences of up to $|Z|\geq 3.8$ in the direction of more Neandertal sharing for Zlatý kůň. Note that in addition to quality differences, these comparisons are also influenced by differences in Neandertal ancestry. All comparisons with $|Z|>2$ compare Zlatý kůň to Kostenki14 or the Sunghir individuals; none of the comparisons between Ust'-Ishim and Zlatý kůň reach $|Z|>1.5$.

Supplementary Table 13.

Neandertal ancestry estimates (α) from the ratio S1/S2 with S1=S(Sample, Mbuti, Altai Neandertal, ApeAncestor) and S2=S(Sample, Vindija N., Altai N., ApeAncestor). S1err and S2err give 5Mb block jackknife on S1 and S2, respectively. Stderr is calculated from S1err and S2err using uncertainty propagation (see text).

Sample	S1	S2	S1err	S2err	α	stderr
Zlatý kůň	5442	170243	542.508	329.779	3.2%	0.32%
Ust'Ishim	4809	175489	517.143	338.306	2.7%	0.29%
Loschbour	3675	175476	434.176	338.111	2.1%	0.25%
Kostenki14	4098	150327	418.17	291.67	2.7%	0.28%
Sunghir1	2860	125207	347.264	253.849	2.3%	0.28%
Sunghir2	4664	173724	474.663	334.395	2.7%	0.27%
Sunghir3	4704	175452	478.161	338.135	2.7%	0.27%
Sunghir4	5147	173411	487.796	334.789	3.0%	0.28%

Supplementary Table 14.

Neandertal ancestry estimates (α) from the ratio S1/S2 with S1=S(Sample, Mbuti, Altai Neandertal, ApeAncestor) and S2=S(Sample, Vindija N., Altai N., ApeAncestor). S1err and S2err give 5Mb block jackknife on S1 and S2, respectively. Stderr is calculated from S1err and S2err using uncertainty propagation (see text). Each row gives the estimate for a comparison between a specific sample (left side of the table) and Zlatý kůň (right side). Only sites covered in this sample and Zlatý kůň were used in the calculation.

	Estimate in Sample						Estimate in Zlatý kůň					
**Sample**	**S1**	**S2**	**S1err**	**S2err**	**α**	**stderr**	**S1**	**S2**	**S1err**	**S2err**	**α**	**stderr**
Ust_Ishim	4692	170231	503.589	329.798	2.76%	0.30%	5438	170231	542.494	329.798	3.19%	0.32%
Loschbour	3599	170221	424.743	329.707	2.11%	0.25%	5435	170221	542.451	329.707	3.19%	0.32%
Kostenki14	3983	146129	409.115	284.89	2.73%	0.28%	4810	146129	465.896	284.89	3.29%	0.32%
Sunghir1	2790	121667	340.63	248.418	2.29%	0.28%	3965	121667	407.964	248.418	3.26%	0.34%
Sunghir2	4488	168549	462.043	326.132	2.66%	0.27%	5385	168549	537.562	326.132	3.19%	0.32%
Sunghir3	4552	170197	464.056	329.787	2.67%	0.27%	5435	170197	542.181	329.787	3.19%	0.32%
Sunghir4	4986	168264	474.863	326.195	2.96%	0.28%	5368	168264	536.32	326.195	3.19%	0.32%

Supplementary Table 15.

Neandertal ancestry estimates (α) from the ratio S1/S2 with S1=S(Sample, Mbuti, Altai Neandertal, Denisovan) and S2=S(Sample, Vindija N., Altai N., Denisovan). S1err and S2err give 5Mb block jackknife on S1 and S2, respectively. Stderr is calculated from S1err and S2err using uncertainty propagation (see text). Each row gives the estimate for a comparison between a specific sample (left side of the table) and Zlatý kůň (right side). Only sites covered in this sample and Zlatý kůň were used in the calculation.

	Estimate in Sample						Estimate in Zlatý kůň					
**Sample**	**S1**	**S2**	**S1err**	**S2err**	**α**	**stderr**	**S1**	**S2**	**S1err**	**S2err**	**α**	**stderr**
Ust_Ishim	2314	92146	246.967	182.396	2.51%	0.27%	3008	92146	265.507	182.396	3.26%	0.29%
Loschbour	1790	92147	228.763	182.408	1.94%	0.25%	3009	92147	265.603	182.408	3.27%	0.29%
Kostenki14	1970	79145	220.891	157.489	2.49%	0.28%	2676	79145	229.462	157.489	3.38%	0.29%
Sunghir1	1265	65949	178.698	137.89	1.92%	0.27%	2122	65949	206.003	137.89	3.22%	0.31%
Sunghir2	2018	91286	242.536	180.267	2.21%	0.27%	2989	91286	264.201	180.267	3.27%	0.29%
Sunghir3	2202	92140	243.662	182.49	2.39%	0.26%	3012	92140	265.462	182.49	3.27%	0.29%
Sunghir4	2242	91138	250.818	180.545	2.46%	0.28%	2976	91138	262.069	180.545	3.27%	0.29%

Supplementary Table 16.

Relationship to late Neandertals for early modern human hunter gatherers. D-statistics were calculated based on transversions. Z gives standard errors based on 5Mb weighted block jackknifing.

X=	Zlatý kůň		Ust'Ishim		Kostenki14		Sunghir1		Sunghir2		Sunghir3		Sunghir4	
**Comparison**	**D**	**Z**	**D**	**Z**	**D**	**Z**	**D**	**Z**	**D**	**Z**	**D**	**Z**	**D**	**Z**
D(Goyet,LesCottes,X,ApeAncestor)	5.7%	2.2	4.5%	1.7	8.2%	3.0	7.2%	2.5	11.8%	4.5	9.6%	3.7	10.3%	3.8
D(Goyet,Mezmaiskaya2,X,ApeAncestor)	-1.4%	-0.5	2.9%	1.0	0.0%	0.0	-1.2%	-0.3	4.2%	1.3	4.3%	1.3	2.0%	0.6
D(Goyet,Spy,X,ApeAncestor)	3.1%	0.7	2.2%	0.5	5.6%	1.0	15.1%	2.7	8.7%	1.7	10.2%	2.0	7.8%	1.5
D(Goyet,VindijaG1,X,ApeAncestor)	-3.0%	-0.9	-3.9%	-1.1	-1.0%	-0.3	-3.5%	-0.8	-1.4%	-0.4	0.8%	0.2	-3.4%	-0.9
D(LesCottes,Mezmaiskaya2,X,ApeAncestor)	-3.5%	-1.8	-5.0%	-2.6	-7.1%	-3.4	-4.9%	-2.2	-7.2%	-3.5	-5.5%	-2.7	-5.9%	-3.0
D(LesCottes,VindijaG1,X,ApeAncestor)	-6.4%	-3.1	-7.5%	-3.6	-11.4%	-4.9	-6.3%	-2.6	-9.0%	-4.1	-9.2%	-4.3	-11.6%	-5.4
D(LesCottes,Spy,X,ApeAncestor)	6.0%	2.3	0.6%	0.2	1.4%	0.5	7.9%	2.5	0.5%	0.2	2.9%	1.0	-1.1%	-0.4
D(Mezmaiskaya2,VindijaG1,X,ApeAncestor)	0.6%	0.2	1.2%	0.5	0.2%	0.1	1.2%	0.4	-0.4%	-0.1	-0.4%	-0.1	-1.1%	-0.4
D(Mezmaiskaya2,Spy,X,ApeAncestor)	6.8%	2.1	2.3%	0.7	9.7%	2.7	13.8%	3.6	10.4%	3.1	12.8%	4.0	10.3%	3.0
D(Spy,VindijaG1,X,ApeAncestor)	-3.4%	-1.0	-3.2%	-0.8	-5.0%	-1.3	-11.5%	-2.7	-6.4%	-1.7	-6.0%	-1.6	-6.7%	-1.7

Supplementary Table 17.

Relationship to late Neandertals for early modern human hunter gatherers. D-statistics were calculated based on transversions. Z gives standard errors based on 5Mb weighted block jack-knifing.

X=	Zlatý kůň		Ust'Ishim		Kostenki14		Sunghir1		Sunghir2		Sunghir3		Sunghir4	
**Comparison**	**D**	**Z**	**D**	**Z**	**D**	**Z**	**D**	**Z**	**D**	**Z**	**D**	**Z**	**D**	**Z**
D(Goyet,LesCottes,X,Denisova)	-5.9%	-2.2	-7.2%	-2.7	-5.8%	-2.0	-6.6%	-2.2	-1.4%	-0.5	-3.7%	-1.4	-3.0%	-1.1
D(Goyet,Mezmaiskaya2,X,Denisova)	-5.3%	-1.8	-2.2%	-0.7	-4.8%	-1.4	-5.9%	-1.7	-1.3%	-0.4	-1.2%	-0.4	-2.8%	-0.9
D(Goyet,Spy,X,Denisova)	-9.0%	-2.0	-11.0%	-2.5	-7.9%	-1.6	-4.9%	-0.9	-6.8%	-1.4	-5.9%	-1.2	-7.7%	-1.6
D(Goyet,VindijaG1,X,Denisova)	-6.2%	-1.8	-7.1%	-2.2	-6.8%	-1.9	-4.2%	-1.1	-4.5%	-1.3	-3.4%	-1.0	-6.2%	-1.8
D(LesCottes,Mezmaiskaya2,X,Denisova)	2.5%	1.2	1.4%	0.6	1.3%	0.5	1.5%	0.6	-0.5%	-0.2	1.3%	0.6	0.9%	0.4
D(LesCottes,VindijaG1,X,Denisova)	2.7%	1.1	1.9%	0.8	-0.8%	-0.3	4.9%	1.9	1.0%	0.4	1.3%	0.6	-1.5%	-0.6
D(LesCottes,Spy,X,Denisova)	5.5%	1.9	1.3%	0.5	1.4%	0.5	7.3%	2.1	0.8%	0.3	4.1%	1.4	0.2%	0.1
D(Mezmaiskaya2,VindijaG1,X,Denisova)	-0.9%	-0.3	-0.2%	-0.1	-2.0%	-0.7	-0.7%	-0.2	-1.5%	-0.5	-1.5%	-0.5	-2.2%	-0.8
D(Mezmaiskaya2,Spy,X,Denisova)	-0.8%	-0.3	-5.3%	-1.7	-0.6%	-0.2	1.3%	0.4	1.8%	0.5	3.9%	1.2	2.6%	0.8
D(Spy,VindijaG1,X,Denisova)	7.1%	2.0	7.8%	2.1	5.8%	1.4	2.7%	0.6	5.7%	1.5	5.6%	1.5	4.7%	1.2

Supplementary Table 18.

Relationship to late Neandertals for early modern human hunter gatherers. D-statistics were calculated based on transversions. Z gives standard errors based on 5Mb weighted block jack-knifing.

X=	Zlatý kůň		Ust'Ishim		Kostenki14		Sunghir1		Sunghir2		Sunghir3		Sunghir4	
**Comparison**	**D**	**Z**	**D**	**Z**	**D**	**Z**	**D**	**Z**	**D**	**Z**	**D**	**Z**	**D**	**Z**
D(Goyet,LesCottes,X,Mbuti)	-6.9%	-2.1	-10.0%	-3.0	-10.7%	-2.9	-7.2%	-1.8	-4.2%	-1.2	-3.3%	-1.0	-3.3%	-0.9
D(Goyet,Mezmaiskaya2,X,Mbuti)	-5.2%	-1.4	-1.8%	-0.4	-10.5%	-2.4	-0.6%	-0.1	-2.5%	-0.6	-3.1%	-0.7	-0.2%	0.0
D(Goyet,Spy,X,Mbuti)	-4.5%	-0.8	-12.0%	-2.1	-5.5%	-0.8	3.0%	0.4	0.4%	0.1	0.8%	0.1	6.2%	0.9
D(Goyet,VindijaG1,X,Mbuti)	-0.8%	-0.2	-4.7%	-1.0	-7.2%	-1.5	0.0%	0.0	-0.4%	-0.1	-1.9%	-0.4	3.8%	0.8
D(LesCottes,Mezmaiskaya2,X,Mbuti)	8.3%	3.4	8.5%	3.3	5.8%	2.0	5.8%	2.0	7.4%	2.8	5.6%	2.1	9.1%	3.6
D(LesCottes,VindijaG1,X,Mbuti)	0.9%	0.3	1.5%	0.5	-1.2%	-0.4	0.7%	0.2	0.6%	0.2	-4.0%	-1.4	0.1%	0.0
D(LesCottes,Spy,X,Mbuti)	5.9%	1.8	2.4%	0.7	1.9%	0.5	6.2%	1.4	4.0%	1.1	6.4%	1.8	-0.8%	-0.2
D(Mezmaiskaya2,VindijaG1,X,Mbuti)	-3.8%	-1.1	-5.2%	-1.6	-4.5%	-1.3	-2.4%	-0.6	-3.0%	-0.9	-5.3%	-1.5	-6.1%	-1.9
D(Mezmaiskaya2,Spy,X,Mbuti)	-4.0%	-1.0	-13.1%	-3.2	0.4%	0.1	0.0%	0.0	2.0%	0.4	1.1%	0.3	-1.9%	-0.5
D(Spy,VindijaG1,X,Mbuti)	5.7%	1.3	1.7%	0.4	0.8%	0.2	-5.1%	-0.9	2.7%	0.5	-1.7%	-0.4	1.1%	0.2

Supplementary Table 19.

Relationship to late Neandertals for early modern human hunter gatherers. D-statistics were calculated based on transversions. Column Z gives standard errors based on 5Mb weighted block jackknifing. Table is sorted by Z-scores.

Comparison	D	Z
D(ZlatyKun,UstIshim,Denisova,Mbuti)	-0.60%	-0.8
D(ZlatyKun,Sunghir3,Denisova,Mbuti)	0.00%	0.1
D(ZlatyKun,Sunghir4,Denisova,Mbuti)	0.10%	0.1
D(ZlatyKun,UstIshim,Goyet,Mbuti)	0.30%	0.2
D(ZlatyKun,Sunghir2,Denisova,Mbuti)	0.20%	0.2
D(ZlatyKun,Sunghir4,VindijaG1,Mbuti)	0.50%	0.5
D(ZlatyKun,UstIshim,VindijaG1,Mbuti)	0.70%	0.6
D(ZlatyKun,UstIshim,Altai,Mbuti)	0.70%	0.6
D(ZlatyKun,UstIshim,Vindija,Mbuti)	0.80%	0.6
D(ZlatyKun,Kostenki14,Denisova,Mbuti)	0.50%	0.6
D(ZlatyKun,UstIshim,LesCottes,Mbuti)	1.00%	0.8
D(ZlatyKun,Sunghir4,Goyet,Mbuti)	1.20%	0.9
D(ZlatyKun,UstIshim,Mezmaiskaya2,Mbuti)	1.20%	1.0
D(ZlatyKun,Sunghir3,VindijaG1,Mbuti)	1.30%	1.1
D(ZlatyKun,Sunghir4,Altai,Mbuti)	1.20%	1.1
D(ZlatyKun,Sunghir4,Vindija,Mbuti)	1.30%	1.2
D(ZlatyKun,Sunghir4,Spy,Mbuti)	1.60%	1.3
D(ZlatyKun,Sunghir1,Denisova,Mbuti)	1.00%	1.3
D(ZlatyKun,Kostenki14,VindijaG1,Mbuti)	1.60%	1.3
D(ZlatyKun,Sunghir3,Altai,Mbuti)	1.50%	1.4
D(ZlatyKun,Sunghir4,Mezmaiskaya2,Mbuti)	1.70%	1.5
D(ZlatyKun,UstIshim,Spy,Mbuti)	2.00%	1.5
D(ZlatyKun,Sunghir4,LesCottes,Mbuti)	1.70%	1.6
D(ZlatyKun,Sunghir3,Vindija,Mbuti)	1.70%	1.6
D(ZlatyKun,Kostenki14,Goyet,Mbuti)	2.20%	1.7
D(ZlatyKun,Sunghir3,Goyet,Mbuti)	2.10%	1.7
D(ZlatyKun,Kostenki14,Mezmaiskaya2,Mbuti)	2.10%	1.8
D(ZlatyKun,Sunghir1,VindijaG1,Mbuti)	2.30%	2.0
D(ZlatyKun,Sunghir1,Goyet,Mbuti)	2.60%	2.0
D(ZlatyKun,Sunghir2,VindijaG1,Mbuti)	2.30%	2.0
D(ZlatyKun,Sunghir2,Vindija,Mbuti)	2.20%	2.0
D(ZlatyKun,Sunghir3,Mezmaiskaya2,Mbuti)	2.40%	2.1
D(ZlatyKun,Sunghir2,Altai,Mbuti)	2.20%	2.1

D(ZlatyKun,Kostenki14,Altai,Mbuti)	2.30%	2.1
D(ZlatyKun,Kostenki14,Vindija,Mbuti)	2.50%	2.2
D(ZlatyKun,Sunghir3,LesCottes,Mbuti)	2.50%	2.2
D(ZlatyKun,Sunghir2,Goyet,Mbuti)	2.80%	2.3
D(ZlatyKun,Sunghir2,Mezmaiskaya2,Mbuti)	2.60%	2.3
D(ZlatyKun,Sunghir1,LesCottes,Mbuti)	2.70%	2.5
D(ZlatyKun,Kostenki14,LesCottes,Mbuti)	2.90%	2.6
D(ZlatyKun,Sunghir1,Mezmaiskaya2,Mbuti)	3.20%	2.7
D(ZlatyKun,Sunghir2,LesCottes,Mbuti)	2.90%	2.7
D(ZlatyKun,Sunghir1,Vindija,Mbuti)	3.20%	2.9
D(ZlatyKun,Sunghir1,Altai,Mbuti)	3.10%	3.0
D(ZlatyKun,Sunghir3,Spy,Mbuti)	3.80%	3.0
D(ZlatyKun,Kostenki14,Spy,Mbuti)	4.40%	3.4
D(ZlatyKun,Sunghir2,Spy,Mbuti)	4.50%	3.7
D(ZlatyKun,Sunghir1,Spy,Mbuti)	4.90%	3.8

Supplementary Information S7

Neandertal ancestry along the genome

Neandertal ancestry informative sites

We used the data of the 1000 Genomes project[78], the Vindija Neandertal[69] and the alignments of the genomes of ape and monkey outgroups[79–83] (chimpanzee (panTro4), bonobo (pan-pan2.1), gorilla (gorGor3), orangutan (ponAbe2), rhesus macaque (rheMac3)) to identify sites that are informative for Neandertal ancestry in modern humans. After applying the published recommended filters for the Vindija Neandertal, we determined the ancestral state by requiring all ape and monkey outgroups to carry the same base. If the Vindija Neandertal carried a different base and that base was present in no more than 1 out of 1004 African haplotypes from the 1000 Genomes project, then this site was considered a Neandertal ancestry informative site.

At each informative position, a random overlapping sequence was selected from the alignments of Zlatý kůň (single-stranded shotgun data), Ust'Ishim[64], Loschbour[76], Sunghir1-4[74] and Kostenki14[75] after filtering for a minimum mapping quality of 25 (for single-stranded library data) or 30 (double-stranded data) and a minimum base quality of 30. Supplementary Table 20 gives the counts of shared Neandertal derived and ancestral alleles for each ancient individual. Supplementary Figure 12 shows the distribution of shared Neandertal ancestry informative alleles in Ust'-Ishim and Kostenki 14 (see Fig.3b for distribution in Zlatý kůň).

Calling regions of Neandertal ancestry

We used a hidden Markov model to determine regions that are likely of Neandertal origin. The hidden states of the model correspond to Neandertal ancestry in heterozygous state and no Neandertal ancestry. Note that homozygous Neandertal ancestry is rarely observed due to the generally low Neandertal ancestry of <3% in modern humans.

We use the Baum-Welch algorithm (as implemented in Lin Himmelmann's HMM package for R) to fit the emission and transition probabilities to the data. Initial emission probabilities for the shared derived state was set to 50% within Neandertal regions and to 10% outside. Transitions between the hidden states had initial probabilities of 20%. Supplementary Table 21 gives the fitted parameters for all genomes.

Posterior decoding was used to determine the posterior probabilities of Neandertal ancestry at each informative position. Sites with a posterior probability between 0.2 and 0.8 were excluded. Runs of posterior probabilities >0.8 were called as introgressed. The genetic length in Morgan was determined for called regions using either the African American recombination map[84] or the Decode recombination map[85].

Time since introgression based on called Neandertal ancestry regions

Following the idea from Fu et al. 2015[56], we estimate the time since the last admixture from the longest introgressed regions. The lengths of regions are assumed to be exponentially distributed with mean length 1/(g-1) Morgan, where g is the number of generations since admixture. The denominator for the mean length is g-1 since the first recombination between introgressed and non-introgressed chromosomes occurs in the first generation offspring, i.e. one generation after admixture. Choosing the n=100 largest regions, denoted as $l_1, \ldots, l_{100}$ in order of descending length, we apply the property of memorylessness of the exponential distribution, $P(X > l_{100} + z | X > l_{100}) = P(X > z)$, to estimate the rate parameter λ, corresponding to g-1, as $\hat{\lambda} = 98 / \sum_{i=1}^{100} l_i - l_{100}$ using the mean square estimator[86,87]. 95% confidence intervals were calculated with the χ^2 distribution as $\left[\hat{\lambda} \times \frac{\chi^2_{df=200}(0.025)}{200}, \hat{\lambda} \times \frac{\chi^2_{df=200}(0.975)}{200}\right]$. Supplementary Table 22 gives the estimates for the number of generations since admixture based on the called Neandertal ancestry segments in the tested hunter-gatherer genomes.

Supplementary Figure 13 shows the 100 longest regions plotted against the logarithm of their rank, which provides an estimate of the position on the y-scale according to the inverse cumulative distribution function. The rank was calculated as k-1/2 where k denotes the 1-based position in the list of regions sorted in descending order by length. Approximate estimates of the generations since admixture that are based on the slope s of the linear fits were calculated as -1*s+1 (Supplementary Table 23). Extended Data Figure 9 shows the proportion of the diploid genome of Zlatý kůň that was covered by the 100 longest regions.

Overlap with Neandertal deserts

We test whether the called Neandertal ancestry in any of the ancient genomes overlaps known deserts of Neandertal ancestry in present-day human genomes. High-coverage genomes were not restricted to Zlatý kůň sites for this analysis. Excluding regions shorter than 10,000 basepairs, we find three instances of overlap with the six regions of depleted Neandertal ancestry from Table S8 in Vernot et al. (2016)[88]. These overlapping regions were called in Sunghir1, Loschbour and Zlatý kůň, respectively. The region in Sunghir1 is only supported by two Neandertal shared-derived SNPs and is plausibly caused by sequencing error. The region in Loschbour is supported by 6 Neandertal shared-derived among 8 SNPs, total, and overlap only the start of desert region chr7:106300000-124700000. Only two sites are retained if Loschbour is filtered for sites that are covered in Zlatý kůň and both are Neandertal shared-derived. The region in Zlatý kůň (chr1:112723662-112855462) falls within the desert region chr1:102200000-114900000 and is supported by 11 Neandertal shared-derived SNPs among 12 SNPs, total (see also Extended Data Figure 8).

The called Neandertal segment in Zlatý kůň does not overlap known protein-coding genes. The desert region contains, among many others, the Amy1A gene that codes for the salivary Alpha-amylase 1 enzyme responsible for the digestion of dietary starch.

Estimates with *admixfrog*

We used introgression caller admixfrog[89] to infer introgressed regions in Zlatý kůň, Ust'-Ishim, Loschbour, Kostenki14 and the four Sunghir genomes. The sources of ancestry were set to the diploid Vindija Neandertal genome and the genomes of the African 1000 genomes individuals. An ancestral state was inferred from ape and monkey outgroups as described above. Two versions of the reference files were generated, one with the African American recombination map and one with the Decode recombination map. Inputfiles for the ancient genomes were produced by admixfrog-bam and admixfrog was run with on these inputfiles with parameters "--c0 0 --dont-est-contamination --run-penalty 0.4". If overlapping introgressed regions were called for the heterozygous and homozgygous state, the longer region was chosen.

Time since introgression was calculated as described above and the results are shown in Supplementary Table 24.

Overlapping regions of Neandertal ancestry are called for the desert on chromosome 1 (African-American map: 1:112688737-112855127; Decode: 1:112690698-112850499).

Supplementary Table 20.

Number of shared derived and ancestral Neandertal informative sites.

Sample	Ancestral	Shared-derived	Fraction shared-derived
Zlatý kůň (~3.2x single-stranded data)	311103	8442	0.026
Zlatý kůň (~0.6x double-stranded data)	162241	4480	0.027
Ust'Ishim	376275	9404	0.024
Loschbour	378873	6531	0.017
Sunghir1	268299	4819	0.018
Sunghir2	373500	7363	0.019
Sunghir3	377191	7976	0.021
Sunghir4	371799	8236	0.022
Kostenki14	323693	6420	0.019

Supplementary Table 21.

Baum-Welch fitted emission and transition probabilities for the Neandertal ancestry hidden Markov model. "N" and "¬N" refer to the Neandertal state and non-Neandertal state, respectively. The columns for emission probabilities give the probability of encountering a shared Neandertal derived site.

Sample	Transition prob.				Emission Prob.	
	N→N	N→¬N	¬N→N	¬N→¬N	N	¬N
Zlatý kůň (~3.2x single-stranded data)	0.986	0.014	0.001	0.999	0.425	0.003
Zlatý kůň (~0.6x double-stranded data)	0.980	0.020	0.001	0.999	0.434	0.003
Ust'Ishim	0.981	0.019	0.001	0.999	0.450	0.004
Loschbour	0.919	0.081	0.002	0.998	0.533	0.003
Sunghir1	0.954	0.046	0.001	0.999	0.493	0.003
Sunghir2	0.964	0.036	0.001	0.999	0.498	0.003
Sunghir3	0.961	0.039	0.001	0.999	0.484	0.003
Sunghir4	0.966	0.034	0.001	0.999	0.484	0.003
Kostenki14	0.973	0.027	0.001	0.999	0.455	0.002

Supplementary Table 22.

Estimates of the number of generations since the last Neandertal admixture based on the lengths of the 100 longest regions calculated with the African American or Decode recombination maps. CI low and CI high give the 2.5% - 97.5% confidence interval.

Sample	Decode recombination map			African American map		
	estimate	CI low	CI high	estimate	CI low	CI high
Zlatý kůň (~3.2x single-stranded data)	78.4	63.9	94.2	74.3	60.7	89.4
Zlatý kůň (~0.6x double-stranded data)	79.8	65.1	95.9	74.5	60.8	89.6
Ust'Ishim	99.1	80.8	119.3	94.2	76.8	113.3
Loschbour	426.9	347.5	514.4	387.9	315.8	467.4
Sunghir1	267.1	217.5	321.7	256.3	208.7	308.7
Sunghir2	304.3	247.8	366.6	263.4	214.5	317.3
Sunghir3	254.5	207.2	306.5	212.8	173.3	256.3
Sunghir4	239.3	194.9	288.2	235.0	191.4	283.0
Kostenki14	195.7	159.5	235.7	175.0	142.6	210.7

Supplementary Table 23.

Estimates of the number of generations since the last Neandertal admixture based on the slope of the fitted line in Supplementary Figure 12.

Sample	Decode recombination map	African American map
Zlatý kůň (~3.2x single-stranded data)	74.4	71.9
Zlatý kůň (~0.6x double-stranded data)	72.8	70.3
Ust'Ishim	90.7	88.2
Loschbour	354.8	340.2
Sunghir1	230.2	237.0
Sunghir2	302.0	299.0
Sunghir3	229.2	185.6
Sunghir4	211.9	214.3
Kostenki14	187.1	185.5

Supplementary Table 24.

Estimates of the number of generations since the last Neandertal admixture for Neandertal ancestry called with admixfrog.

Sample	Admixfrog					
	Decode recombination map			African American map		
	estimate	CI low	CI high	esti-mate	CI low	CI high
Zlatý kůň (~3.2x single-stranded data)	81.5	66.5	98.0	72.1	58.9	86.7
Zlatý kůň (~0.6x double-stranded data)	76.6	62.5	92.1	70.2	57.3	84.4
Ust'Ishim	110.0	89.7	132.3	103.6	84.5	124.7
Loschbour	764.2	622.0	920.9	781.3	635.8	941.4
Sunghir1	316.5	257.7	381.3	340.1	276.9	409.7
Sunghir2	317.1	258.2	382.0	289.8	236.0	349.1
Sunghir3	275.1	224.0	331.4	258.5	210.5	311.3
Sunghir4	262.2	213.5	315.8	245.1	199.6	295.2
Kostenki14	226.4	184.4	272.7	210.7	171.6	253.8

Supplementary Figure 12.

Segments of Neandertal ancestry in Ust'-Ishim and Kostenki14. See Fig. 3b for comparison.

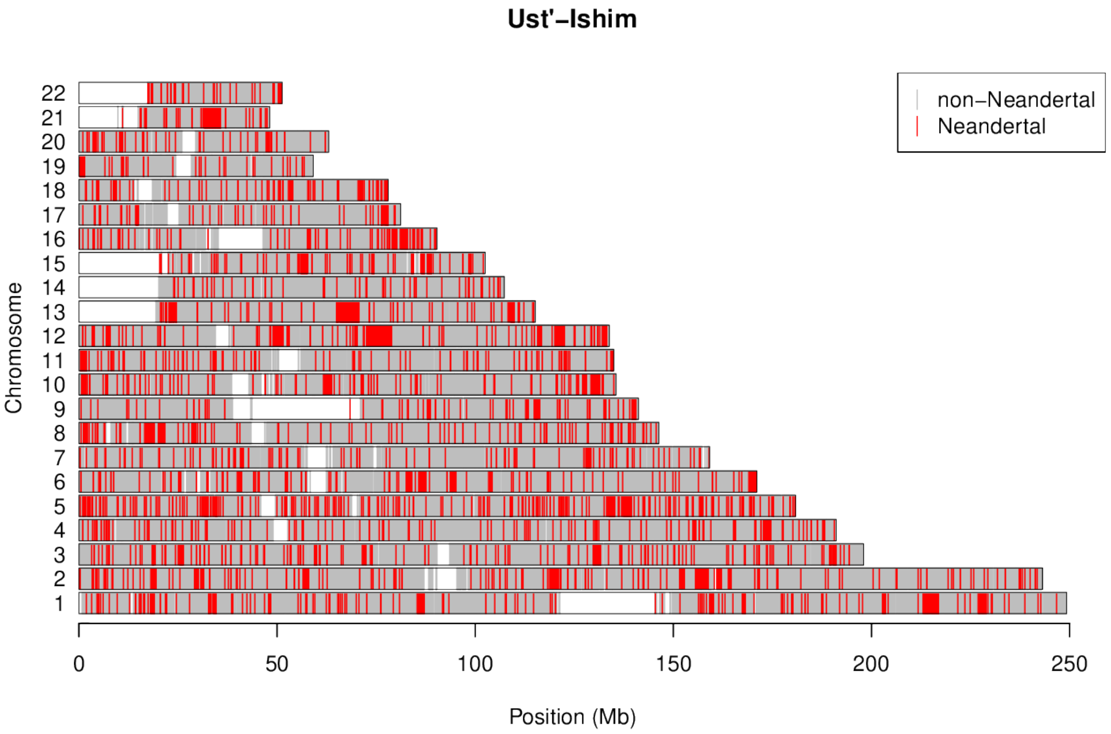

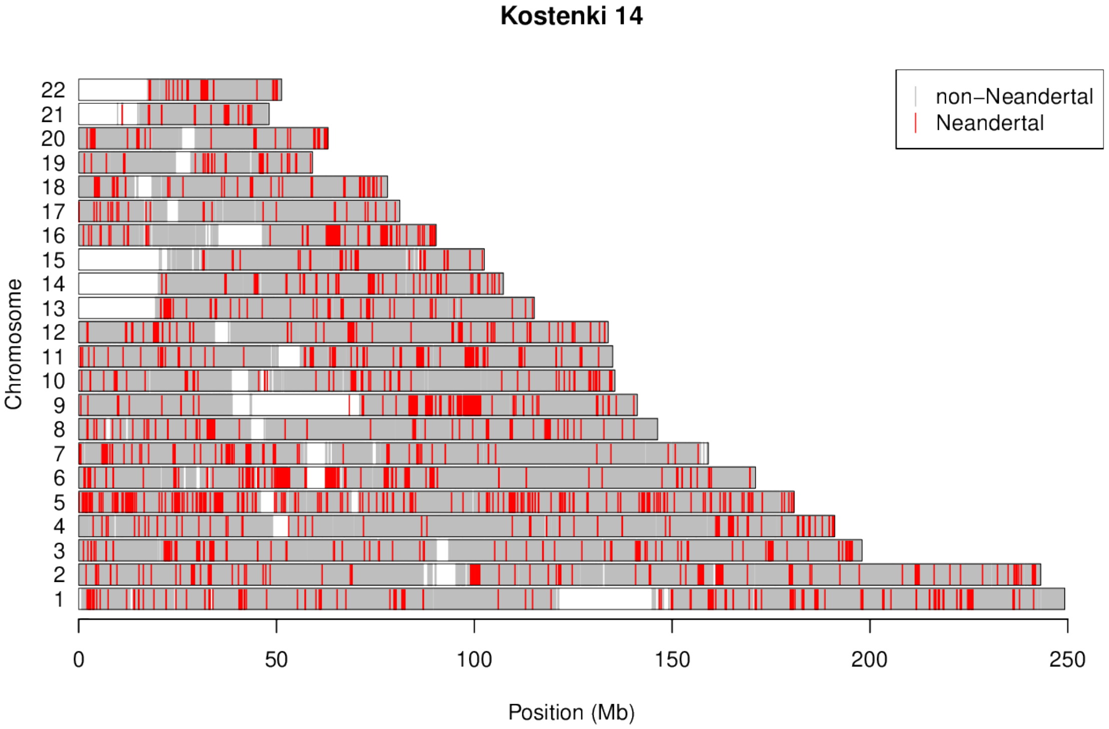

Supplementary Figure 13.

Top 100 introgressed regions for ancient samples and linear fit against rank and size of regions according to the Decode recombination map (top) or the African American recombination map (bottom).

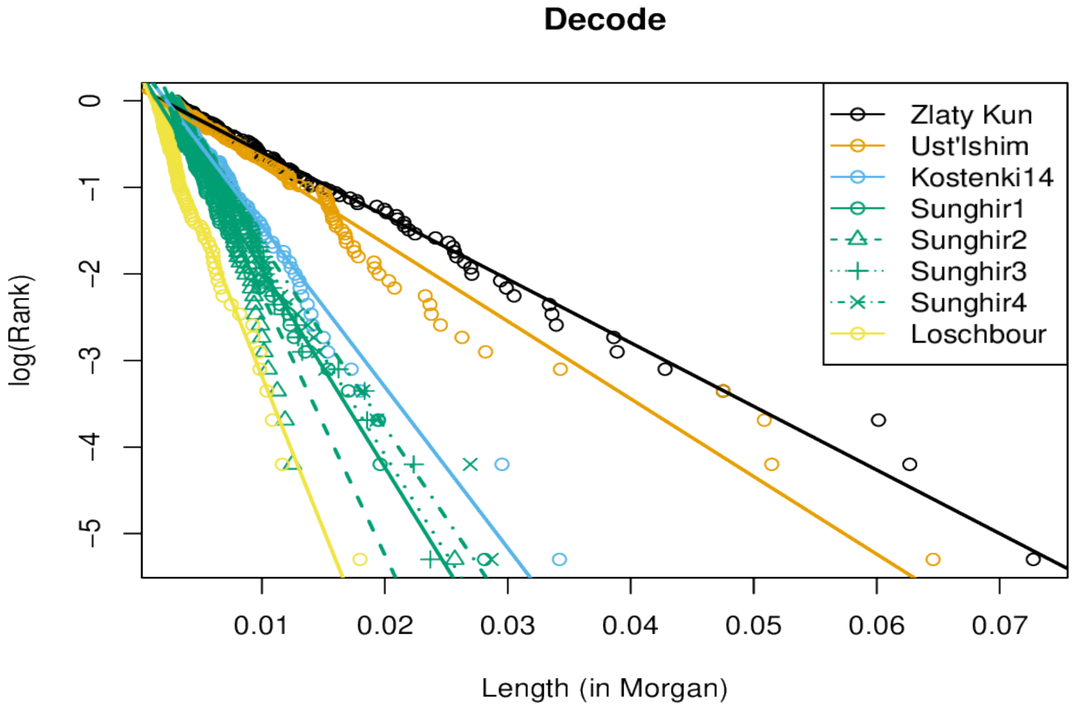

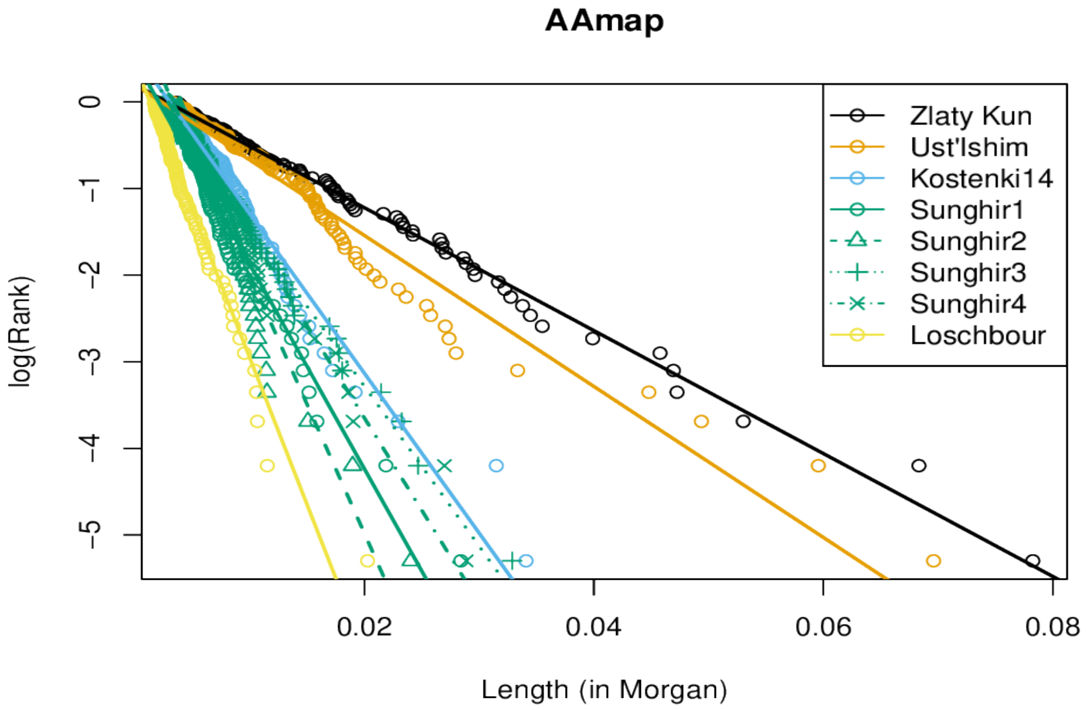

Supplementary Information S8

Dating Neandertal introgression

Estimates with Neandertal dating method

We used the Moorjani et al. Neandertal dating method[90] for Zlatý kůň and other ancient genomes. The method fits an exponential distribution to the slope of covariance of informative sites for Neandertal ancestry at different distances to estimate the date since admixture. For each ancient genome, a sequence with a minimum mapping quality of 25 (single-stranded library sequences) or 30 (double-stranded library sequences) and base quality of 30 was randomly chosen for the informative sites supplied with the Neandertal dating method ("ascertainment0"). Following previous approaches, covariance was calculated in bins of 0.001cM. The exponential distribution was fit with the rexpfit.r script with the affine term option to the range 0.02-1cM or 0.02-10cM. Supplementary Table 25 shows the estimated time since admixture. Zlatý kůň yields the smallest number of generations since Neandertal admixture. As noted before by Moorjani et al., the extended range of 0.02-10cM yields more plausible estimates for samples closer in time to the Neandertal admixture.

We additionally estimated the Neandertal admixture dates for the 0.02-10cM range for only those sites that are covered in the Zlatý kůň double stranded data and the ancient genome used for comparison. Supplementary Table 26 shows the results. Zlatý kůň was found again to be the sample with the shortest time since the Neandertal admixture.

Testing for evidence of two admixture episodes

We attempted to fit a model with two exponential curves to the 0.02-10cM range of bins for all ancient genomes. As before, we run an additional test for the Zlatý kůň double-stranded data and restrict the analysis to sites that overlap between one of the high-coverage ancient samples and this subset of Zlatý kůň's data. The optimization procedure was unable to identify a fit for Loschbour and yields results for both subsets of the Zlatý kůň data that give an overall worse fit than the single exponential. In contrast, Sunghir1-4, Kostenki14 and Ust'Ishim show significantly better fits to a model with two exponentials (Supplementary Table 27). However, some of the estimated parameters are implausibly low (Kostenki14 was estimated to have experienced admixture 1 generation before it lived), suggesting that the numerical optimization has not converged to the correct parameters.

Supplementary Table 25.

Estimated number of generations since Neandertal admixture for bins in the range of 0.02-1cM or 0.02-10cM from a fit of an exponential distribution with affine term [90].

Sample	Range 0.02-1cM		Range 0.02-10cM	
	Generations	stderr	Generations	stderr
Zlatý kůň (~3.2x single-stranded data)	170.5	25.23	62.7	0.58
Zlatý kůň (~0.6x double-stranded data)	164.2	36.50	60.9	0.79
Ust_Ishim	244.6	26.76	84.2	1.28
Loschbour	1952.0	69.60	1808.0	28.77
Sunghir1	425.5	29.19	166.5	3.16
Sunghir2	278.5	16.19	243.6	2.73
Sunghir3	278.1	16.60	220.5	3.55
Sunghir4	471.7	23.14	211.0	4.40
Kostenki14	205.1	13.87	277.3	3.67

Supplementary Table 26.

Estimated number of generations since Neandertal admixture for bins in the range of 0.02-10cM from a fit of an exponential distribution with affine term[90]. Each row shows the estimate for only those sites that are covered in the column's Sample and Zlatý kůň's ~0.6-fold double stranded data.

Sample estimate			Zlatý kůň double stranded data estimate	
Sample	Generations	stderr	Generations	stderr
Ust_Ishim	84.8	1.64	62.04	0.75
Loschbour	1465.0	29.03	62.04	0.75
Sunghir1	120.6	3.22	61.67	0.93
Sunghir2	240.3	3.36	61.96	0.75
Sunghir3	204.0	3.88	62.05	0.75
Sunghir4	197.9	4.32	62.23	0.76
Kostenki14	240.7	3.58	64.74	0.84

Supplementary Table 27.

Estimated parameters when fitting two exponential distributions with affine term[90], corresponding to the formula $Ae^{-dm} + Be^{-dn} + C$, for bins in the range of 0.02-10cM. LRT gives the likelihood ratio test using a chi-square test with 2 degrees of freedom against a fit with one exponential distribution (see Supplementary Table 26).

Sample	Generations 1	SE	Generations 2	SE	LRT
Zlatý kůň (single-stranded data)	107.0	147.5	79.0	29.0	1
Ust'Ishim	226.2	11.6	27.8	2.4	2.43E-166
Loschbour	no convergence				
Sunghir1	555.4	33.7	69.3	3.8	2.69E-107
Sunghir2	0.6	24.4	260.3	4.3	6.45E-10
Sunghir3	184.6	10.4	695.3	188.1	1.98E-11
Sunghir4	2214.0	314.8	174.4	5.6	1.75E-42
Kostenki14	2.0	9.7	319.3	5.9	5.19E-58
Zlatý kůň (double-stranded data)	100.6	59.7	83.9	38.1	1
Ust'Ishim (restricted to Zlatý kůň double-stranded data sites)	204.1	13.4	24.3	3.4	2.82E-94
Loschbour (restricted to Zlatý kůň double-stranded data sites)	no convergence				
Sunghir1 (restricted to Zlatý kůň double-stranded data sites)	75.9	4.4	771.7	85.0	1.30E-49
Sunghir2 (restricted to Zlatý kůň double-stranded data sites)	288.6	6.7	10.1	5.1	1.37E-68
Sunghir3 (restricted to Zlatý kůň double-stranded data sites)	547.2	174.7	172.9	16.0	9.00E-08
Sunghir4 (restricted to Zlatý kůň double-stranded data sites)	2569.0	499.0	178.0	5.8	6.31E-24
Kostenki14 (restricted to Zlatý kůň double-stranded data sites)	1.5	6.4	282.7	6.4	1.53E-64

Supplementary Information S9

Relationship to Early Upper Palaeolithic hunter gatherers inferred from shotgun data

D-statistics

To calculate D-statistics of the form (HG,HG,HG,outgroup), where HG denotes Upper Palaeolithic hunter-gatherers, we use the inferred ancestor of apes, a high-coverage genome from a present-day Mbuti and a single sampled base from the Upper Palaeolithic hunter-gatherers Ust'Ishim, Kostenki14, Sunghir1,2,3,4 and Zlatý kůň (see previous section on Neandertal ancestry for details on processing). Statistics were calculated only on sites that were bi-allelic and transversions.

We first tested for a difference in relationship to Zlatý kůň. Only some of the comparisons of Sunghir4 and one involving Sunghir3 to other hunter-gatherers yield $|Z|>2$ in the direction of a more distant relationship of Sunghir3/4 to Zlatý kůň. No significant differences were found in other pairwise comparisons of hunter-gatherers to Zlatý kůň (Supplementary Table 28).

To further test the relationship among hunter-gatherers, we calculated D-statistics for all pairwise comparisons to Ust'Ishim and find that Zlatý kůň shows a more distant relationship than all other hunter-gatherers, although this difference is not significant at a threshold of $|Z| >2$ for Sunghir4 (Supplementary Table 29). In pairwise comparisons Ust'Ishim shares more derived alleles with Kostenki14 and all Sunghir genomes than Zlatý kůň does (Supplementary Table 29).

Sunghir individuals and Kostenki share significantly more derived alleles than either Zlatý kůň or Ust'Ishim does (Supplementary Tables 30 and 31).

Divergence from the Ust'Ishim lineage

The D-statistics indicated that Kostenki14 and the Sunghir individuals share more drift than Ust'Ishim or Zlatý kůň. Direct comparisons of Zlatý kůň and Ust'Ishim also suggest that Zlatý kůň is further diverged from Kostenki14 and Sunghir individuals than Ust'Ishim is. We use the F(A|B) statistics that calculates the fraction of shared derived alleles for a genome A where a high-coverage genome B carries a heterozygous genotype. We calculate this statistic for transversion heterozygotes from the genotype calls of the high coverage genomes of the

Vindija and Altai Neandertal, an Mbuti and Ust'Ishim after applying recommended filters. As before, a random allele was drawn from reads aligning at these positions with mapping quality 30 and a base with base quality of at least 30 from the data of Zlatý kůň, Ust'Ishim, Kostenki14 and Sunghir1-4. Triallelic sites were excluded and coverage from all hunter-gatherers was required.

Supplementary Table 32 shows that Zlatý kůň falls within the variation of other hunter-gatherers for the sharing with archaic genomes. Zlatý kůň shares fewer derived alleles with Ust'Ishim than other hunter-gatherers (36.7% compared to >36.9 for others) and more derived alleles with Mbuti (27.3% vs. <27.1% in others). However, these relatively small differences fall in most comparisons within 2 standard errors calculated from a weighted block jackknife over the 22 autosomes.

Table S28.

D-statistics comparing Early and Mid Upper Palaeolithic hunter gather genomes to Zlatý kůň.

Comparison	X=Mbuti		X=ApeAncestor	
	**D**	**Z**	**D**	**Z**
D(Ust'Ishim,Kostenki14,ZlatyKun,X)	1.4%	1.6	-0.1%	-0.1
D(Ust'Ishim,Sunghir1,ZlatyKun,X)	0.5%	0.6	-1.1%	-1.2
D(Ust'Ishim,Sunghir2,ZlatyKun,X)	1.2%	1.5	0.0%	0.1
D(Ust'Ishim,Sunghir3,ZlatyKun,X)	1.4%	1.6	0.7%	0.8
D(Ust'Ishim,Sunghir4,ZlatyKun,X)	2.8%	3.4	1.6%	1.9
D(Kostenki14,Sunghir1,ZlatyKun,X)	-1.1%	-1.3	-1.2%	-1.4
D(Kostenki14,Sunghir2,ZlatyKun,X)	0.2%	0.2	0.3%	0.4
D(Kostenki14,Sunghir3,ZlatyKun,X)	-0.1%	-0.1	0.7%	0.8
D(Kostenki14,Sunghir4,ZlatyKun,X)	1.8%	2.2	2.0%	2.5
D(Sunghir1,Sunghir2,ZlatyKun,X)	0.9%	1.2	1.4%	1.6
D(Sunghir1,Sunghir3,ZlatyKun,X)	1.0%	1.3	2.0%	2.4
D(Sunghir1,Sunghir4,ZlatyKun,X)	2.9%	3.5	3.3%	4.0
D(Sunghir2,Sunghir3,ZlatyKun,X)	0.3%	0.3	0.8%	1.0
D(Sunghir2,Sunghir4,ZlatyKun,X)	1.9%	2.4	1.9%	2.5
D(Sunghir3,Sunghir4,ZlatyKun,X)	1.8%	2.1	1.2%	1.5

Table S29.

D-statistics comparing Early and Mid Upper Palaeolithic hunter gather genomes to Ust'Ishim, and pairwise comparison of Zlatý kůň and Ust'Ishim to other hunter-gatherers (last 5 rows).

Comparison	X=Mbuti		X=ApeAncestor	
	D	Z	D	Z
D(ZlatyKun,Kostenki14,Ust'Ishim,X)	-2.2%	-2.5	-3.1%	-3.5
D(ZlatyKun,Sunghir1,Ust'Ishim,X)	-2.1%	-2.3	-3.3%	-3.6
D(ZlatyKun,Sunghir2,Ust'Ishim,X)	-2.1%	-2.6	-2.8%	-3.4
D(ZlatyKun,Sunghir3,Ust'Ishim,X)	-2.4%	-2.8	-2.7%	-3.1
D(ZlatyKun,Sunghir4,Ust'Ishim,X)	-0.7%	-0.8	-1.5%	-1.6
D(Kostenki14,Sunghir1,Ust'Ishim,X)	-0.2%	-0.2	-0.3%	-0.3
D(Kostenki14,Sunghir2,Ust'Ishim,X)	0.1%	0.1	0.2%	0.3
D(Kostenki14,Sunghir3,Ust'Ishim,X)	-0.2%	-0.2	0.5%	0.6
D(Kostenki14,Sunghir4,Ust'Ishim,X)	1.7%	2.2	1.9%	2.5
D(Sunghir1,Sunghir2,Ust'Ishim,X)	-0.2%	-0.2	0.2%	0.2
D(Sunghir1,Sunghir3,Ust'Ishim,X)	-0.2%	-0.2	0.7%	0.8
D(Sunghir1,Sunghir4,Ust'Ishim,X)	1.5%	1.8	1.8%	2.1
D(Sunghir2,Sunghir3,Ust'Ishim,X)	-0.4%	-0.5	0.2%	0.2
D(Sunghir2,Sunghir4,Ust'Ishim,X)	1.6%	2.0	1.6%	2.0
D(Sunghir3,Sunghir4,Ust'Ishim,X)	2.0%	2.5	1.4%	1.8
D(ZlatyKun,Ust'Ishim,Kostenki14,X)	-3.6%	-4.4	-3.0%	-3.7
D(ZlatyKun,Ust'Ishim,Sunghir1,X)	-2.6%	-3.1	-2.3%	-2.7
D(ZlatyKun,Ust'Ishim,Sunghir2,X)	-3.3%	-4.1	-2.9%	-3.5
D(ZlatyKun,Ust'Ishim,Sunghir4,X)	-3.8%	-4.6	-3.3%	-3.9
D(ZlatyKun,Ust'Ishim,Sunghir3,X)	-3.5%	-4.3	-3.1%	-3.8

Table S30.

D-statistics comparing Zlatý kůň, Sunghir1-4 and Kostenki14.

Comparison	X=Mbuti		X=ApeAncestor	
	D	Z	D	Z
D(ZlatyKun,Sunghir1,Kostenki14,X)	-15.3%	-17.4	-16.2%	-18.4
D(ZlatyKun,Sunghir2,Kostenki14,X)	-14.5%	-17.2	-15.0%	-18.3
D(ZlatyKun,Sunghir3,Kostenki14,X)	-14.5%	-17.4	-14.5%	-17.2
D(ZlatyKun,Sunghir4,Kostenki14,X)	-14.1%	-17.0	-14.6%	-17.3
D(ZlatyKun,Kostenki14,Sunghir1,X)	-14.2%	-15.9	-15.1%	-17.1
D(ZlatyKun,Sunghir2,Sunghir1,X)	-23.3%	-25.3	-23.6%	-26.0
D(ZlatyKun,Sunghir3,Sunghir1,X)	-23.0%	-26.1	-23.0%	-26.0
D(ZlatyKun,Sunghir4,Sunghir1,X)	-20.3%	-21.5	-20.9%	-22.0
D(ZlatyKun,Kostenki14,Sunghir2,X)	-14.7%	-16.8	-15.4%	-17.6
D(ZlatyKun,Sunghir1,Sunghir2,X)	-24.2%	-26.5	-24.9%	-26.9
D(ZlatyKun,Sunghir3,Sunghir2,X)	-27.1%	-28.7	-26.9%	-28.3
D(ZlatyKun,Sunghir4,Sunghir2,X)	-20.7%	-23.9	-21.2%	-24.7
D(ZlatyKun,Kostenki14,Sunghir3,X)	-14.5%	-17.1	-15.1%	-17.5
D(ZlatyKun,Sunghir1,Sunghir3,X)	-24.0%	-26.6	-24.9%	-27.9
D(ZlatyKun,Sunghir2,Sunghir3,X)	-27.3%	-29.6	-27.7%	-30.4
D(ZlatyKun,Sunghir4,Sunghir3,X)	-22.9%	-23.9	-23.4%	-24.9
D(ZlatyKun,Kostenki14,Sunghir4,X)	-15.9%	-19.3	-16.5%	-20.0
D(ZlatyKun,Sunghir1,Sunghir4,X)	-23.0%	-25.0	-24.0%	-26.6
D(ZlatyKun,Sunghir2,Sunghir4,X)	-22.5%	-26.5	-23.0%	-26.6
D(ZlatyKun,Sunghir3,Sunghir4,X)	-24.6%	-26.0	-24.5%	-26.5

Table S31.
D-statistics comparing Ust'Ishim, Sunghir and Kostenki14.

Comparison	X=Mbuti		X=ApeAncestor	
	D	Z	D	Z
D(Ust'Ishim,Sunghir1,Kostenki14,X)	-12.0%	-13.5	-13.2%	-15.4
D(Ust'Ishim,Sunghir2,Kostenki14,X)	-11.3%	-12.9	-12.3%	-14.4
D(Ust'Ishim,Sunghir3,Kostenki14,X)	-11.2%	-13.3	-11.7%	-13.9
D(Ust'Ishim,Sunghir4,Kostenki14,X)	-10.9%	-12.4	-11.9%	-13.6
D(Ust'Ishim,Kostenki14,Sunghir1,X)	-11.8%	-14.1	-13.0%	-15.7
D(Ust'Ishim,Sunghir2,Sunghir1,X)	-21.4%	-24.1	-22.0%	-25.0
D(Ust'Ishim,Sunghir3,Sunghir1,X)	-21.0%	-23.7	-21.2%	-23.3
D(Ust'Ishim,Sunghir4,Sunghir1,X)	-18.5%	-20.6	-19.2%	-20.6
D(Ust'Ishim,Kostenki14,Sunghir2,X)	-11.4%	-13.5	-12.5%	-15.1
D(Ust'Ishim,Sunghir1,Sunghir2,X)	-21.2%	-23.1	-22.2%	-24.2
D(Ust'Ishim,Sunghir3,Sunghir2,X)	-24.5%	-26.1	-24.7%	-26.3
D(Ust'Ishim,Sunghir4,Sunghir2,X)	-18.0%	-20.3	-18.7%	-21.6
D(Ust'Ishim,Kostenki14,Sunghir3,X)	-11.1%	-14.0	-12.2%	-15.4
D(Ust'Ishim,Sunghir1,Sunghir3,X)	-20.8%	-22.3	-21.8%	-23.5
D(Ust'Ishim,Sunghir2,Sunghir3,X)	-24.1%	-26.0	-24.9%	-27.1
D(Ust'Ishim,Sunghir4,Sunghir3,X)	-19.8%	-20.9	-20.6%	-22.0
D(Ust'Ishim,Kostenki14,Sunghir4,X)	-12.6%	-14.6	-13.7%	-16.2
D(Ust'Ishim,Sunghir1,Sunghir4,X)	-20.0%	-21.3	-21.0%	-22.6
D(Ust'Ishim,Sunghir2,Sunghir4,X)	-19.5%	-22.2	-20.3%	-23.1
D(Ust'Ishim,Sunghir3,Sunghir4,X)	-21.7%	-23.4	-21.9%	-23.7

Table S32.

F(A|B) statistics for sharing of derived alleles in the data of individuals shown in rows where high-coverage genomes (columns) carry heterozygote genotype calls. Column stderr gives a block-jackknife over all 22 autosomes weighted by the number of heterozygous sites.

	Vindija		Altai		Denisova		Ust'Ishim		Mbuti	
	f(A|B)	stderr	f(A|B)	stderr	f(A|B)	stderr	f(A|B)	stderr	f(A|B)	stderr
ZlatyKun	10.59%	0.40%	12.47%	0.31%	7.69%	0.37%	36.67%	0.19%	27.33%	0.16%
Ust_Ishim	10.36%	0.38%	12.22%	0.35%	7.67%	0.34%	49.45%	0.10%	27.11%	0.15%
Kostenki14	10.12%	0.40%	12.28%	0.37%	7.71%	0.37%	37.04%	0.17%	27.10%	0.14%
Sunghir1	10.25%	0.36%	12.18%	0.36%	7.67%	0.34%	37.18%	0.17%	27.10%	0.15%
Sunghir2	10.35%	0.42%	12.13%	0.34%	7.63%	0.31%	37.15%	0.19%	27.06%	0.15%
Sunghir3	10.31%	0.36%	12.24%	0.38%	7.63%	0.35%	36.98%	0.20%	27.04%	0.17%
Sunghir4	10.18%	0.38%	12.28%	0.33%	7.66%	0.35%	36.92%	0.18%	27.09%	0.16%

Supplementary Information S10

Computer tomography scan and virtual reconstruction

Micro-computed tomography

As a basis for the virtual reconstruction of the skull as well as for digitally preserving the morphology of the Zlatý kůň cranial remains, the original fossils were micro-CT scanned using the Bruker™ SkyScan 2211 X-ray Nanotomograph housed at the Max Planck Institute for the Science of Human History (MPI SHH) in Jena, Germany. All cranial fossils were scanned simultaneously with an isotropic voxel size of 56.5 μm (120 kV, 180 μA, 0.5 mm copper filter, 360° rotation, 0.18° rotation steps, 400 ms exposure time).

Virtual reconstruction

Virtual reconstruction of the Zlatý kůň skull was done using Avizo 9.4.0. (Visualization Sciences Group). Surface models of the partial neurocranium still glued to the right condyle of the mandible, the partial right maxilla and the partial left zygomatic bone were extracted from the micro-CT images using the 'Isosurface module' in Avizo. No virtual modifications have been done to the initial reconstruction of neurocranium which originally has been manually reassembled from several isolated cranial bones using adhesive material. A detailed overview of all preserved cranial bones is given in Rmoutilova et al. (2018)[17]. Surface models of the cranial parts (neurocranium and mandible still as one surface) were saved in PLY format and imported into GEOMAGIC STUDIO™ 12-20 (Geomagic, Inc.) for necessary surface corrections (e.g., surface closing, etc.) and for separating the mandible from the neurocranium. After subsequent re-importing the four surfaces into Avizo, first the mandible was aligned in anatomical correct position relative to the neurocranium. On the basis of this mandibular alignment the right maxillary was subsequently aligned bringing the heavily worn upper and lower dentition into occlusion. Finally, the partial left zygomatic bone was added to the reconstruction. However, anatomically correct alignment of this surface is less distinct since it does not show any connection with the remaining skull. To reach the best estimation of its correct position and orientation in a first step we flipped the surface into a right zygomatic bone which allowed its alignment relative to the right neurocranium. In a second step we mirror-imaged the right zygomatic resulting in the frontal and lateral reconstructions shown in Supplementary Figure 14 and 15, respectively.

Supplementary Figure 14.

MicroCT-based reconstruction of the Zlatý kůň skull, frontal view.

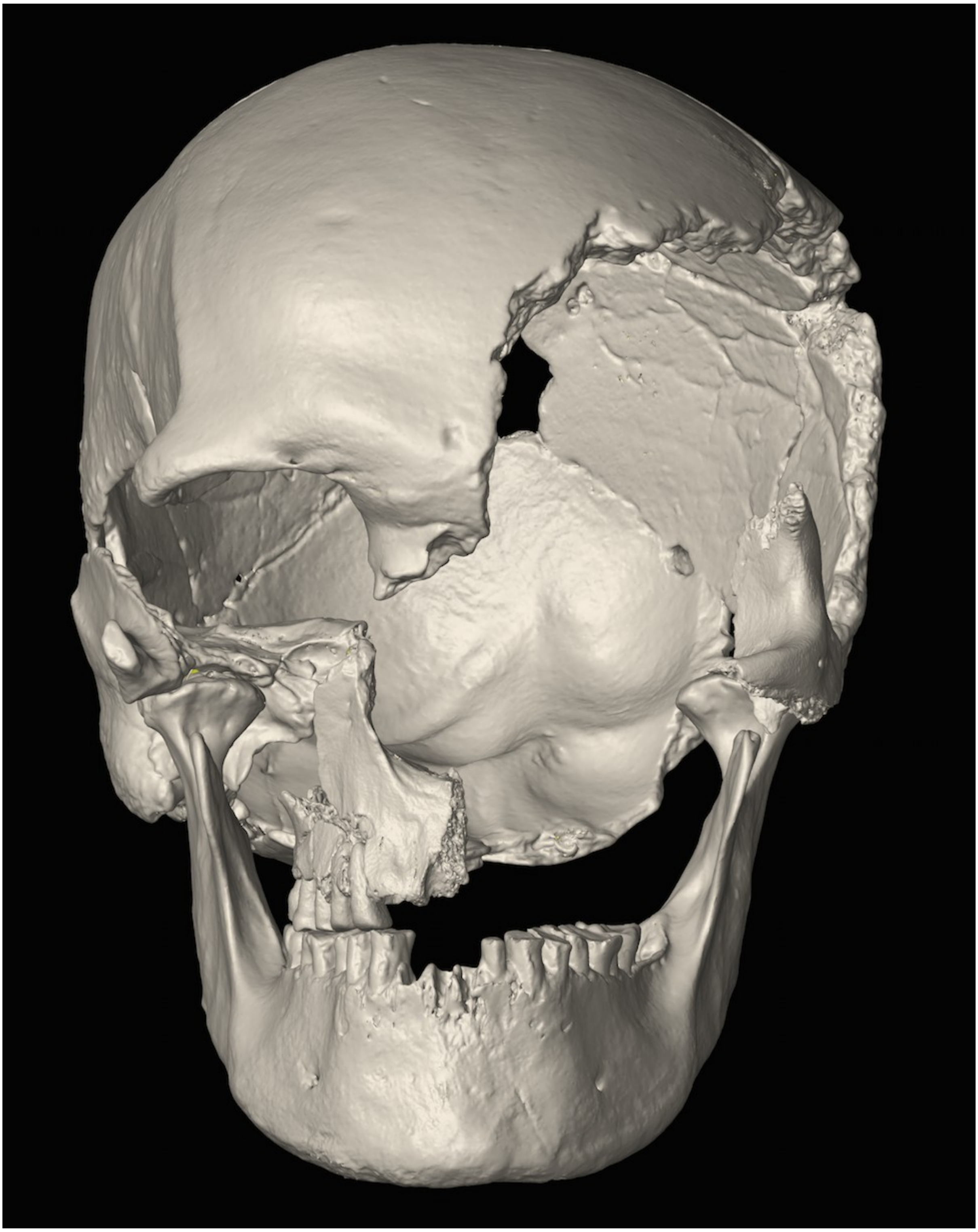

Supplementary Figure 15.

MicroCT-based reconstruction of the Zlatý kůň skull, lateral view.

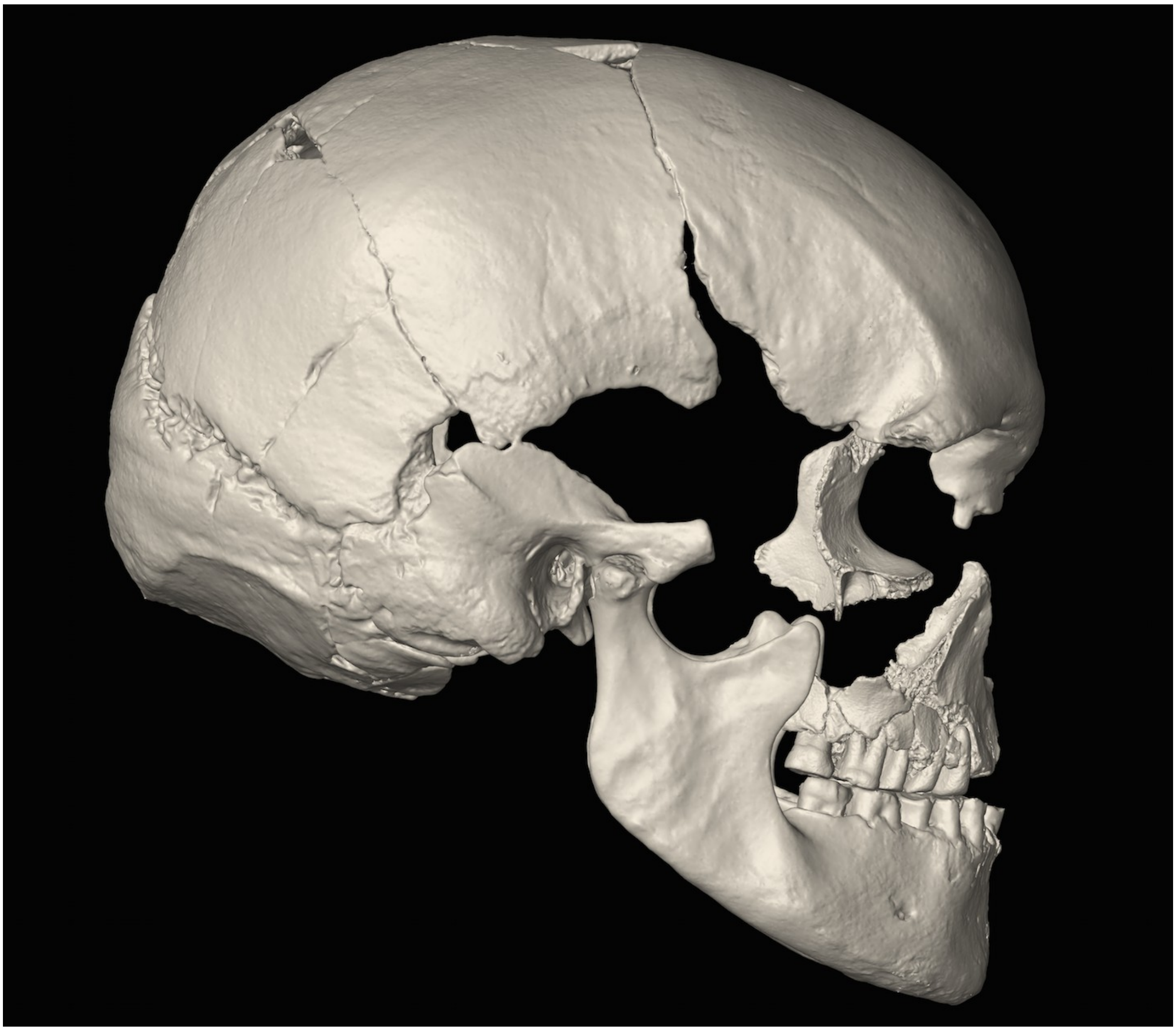

References

1. Mareš, K. The memoirs of the quarryman Mr. Mareš, the discoverer of Koněprusy Caves. (in Czech) Vzpomínky skalníka Mareše, objevitele Koněpruských jeskyní. *Knihovna České společnosti speleologické* **29**, 44–50 (1997).
2. Kukla, J. The report of the result of Zlatý kůň – Cave explorations in 1951 made by the Carstive Section of Nature-Science Club in Prague. (in Czech with German Summary) Zpráva o výsledcích výzkumů jeskyní na Zlatém Koni u Koněprus v roce 1951, prováděných Krasovou sekcí Přírodovědeckého klubu v Praze. *Československý Kras* **5**, 49–68 (1952).
3. Svoboda, J., Kuželka, V. & Vlček, E. The Koněprusy Caves: Depositional context of a human skeleton and the first radiocarbon dating. (in Czech with English summary) Koněpruské jeskyně. Nálezová situace lidského skeletu a první radiokarbonové datování. in *Ve službách archeologie* (eds. Hašek, V., Nekuda, R. & Unger, J.) vol. 4 278–284 (Muzejní a vlastivědná společnost, Brno, 2003).
4. Vlček, E. The pleistocene man from the Zlatý Kůň Cave near Koněprusy. (in Czech with German summary) Pleistocenní člověk z jeskyně na Zlatém koni u Koněprus. *Anthropo-zoikum* **6**, 283–311 (1957).
5. Vlček, E. The Pleistocene Man ("Zlatý Kůň"). (in Czech with German summary) Nález pleistocenního člověka mv jeskyních Zlatého koně. *Československý Kras* **5**, 180–191 (1952).
6. Prošek, F. The research in the Golden Horse Cave near Koněprusy. (in Czech) Výzkum v jeskyni na Zlatém koni u Koněprus. *Archeologické rozhledy* **4**, 206–209 (1952).
7. Vlček, E. The remains of a Pleistocene man from the Zlatý Kůň Cave. (in Czech) Zbytky pleistocenního člověka z jeskyně na Zlatém koni. *Archeologické rozhledy* **4**, 209–213 (1952).
8. Vlček, E. Other findings of the Pleistocene man's remains on Zlatý Kůň near Koněprusy. (in Czech with French summary) Další nálezy pozůstatků pleistocenního člověka na Zlatém Koni u Koněprus. *Archeologické rozhledy* **9**, 305–310 (1957).

9. Vlček, E. Patterns of Human Evolution. in *Hunters between East and West: The Paleolithic of Moravia* (eds. Svoboda, J., Ložek, V. & Vlček, E.) 37–74 (Springer US, 1996). doi:10.1007/978-1-4899-0292-4_3.
10. Jelínek, J. & Orvanová, E. Czech and Slovak Republics. in *Hominid remains -- an update* (eds. Orban, R. & Semal, P.) vol. 9 95–118 (1999).
11. Svoboda, J. The depositional context of the Early Upper Paleolithic human fossils from the Koněprusy (Zlatý kůň) and Mladeč Caves, Czech Republic. *Journal of Human Evolution* **38**, 523–536 (2000).
12. Prošek, F. *et al.* The excavation of the "Zlatý kůň" cave in Bohemia. The report for the 1st research period of 1951 (Part 2). (in Czech with German Summary) Výzkum jeskyně Zlatého Koně u Koněprus. Zpráva za I. výzkumné oddobí r. 1951 (2. část). *Československý kras* **5**, 161–179 (1952).
13. Svoboda, J. & Macholán, M. *Předkové. Evoluce člověka*. (Academia, 2017).
14. Vlček, E. The findings of Pleistocene man in Bohemia. (in Czech) Nálezy pleistocenního člověka v Čechách. *Časopis lékařů českých* **90**, 1457–1462 (1951).
15. Kuželka, V. The postcranial skeletal remains of Pleistocene man from Zlatý kůň near Koněprusy (Bohemia). *Anthropologie (1962-)* **35**, 247–249 (1997).
16. Churchill, S. E. & Smith, F. H. Makers of the early Aurignacian of Europe. *American Journal of Physical Anthropology* **Suppl 31**, 61–115 (2000).
17. Rmoutilová, R. *et al.* Virtual reconstruction of the Upper Palaeolithic skull from Zlatý Kůň, Czech Republic: Sex assessment and morphological affinity. *PLoS ONE* **13**, e0201431 (2018).
18. Svoboda, J. A., Plicht, J. van der & Kuželka, V. Upper Palaeolithic and Mesolithic human fossils from Moravia and Bohemia (Czech Republic): some new 14C dates. *Antiquity* **76**, 957–962 (2002).
19. Zázvorka, V. Preliminary report of the find of fossil bones at the Zlatý Kůň near Koněprusy (Central Bohemia). (in Czech with English summary) Předběžná zpráva o nálezech fosilních kostí na Zlatém koni u Koněprus (Střední Čechy). *Československý Kras* **4**, 60–66 (1951).

20. Diedrich, C. G. & Zak, K. Prey deposits and den sites of the Upper Pleistocene hyena *Crocuta crocuta spelaea* (Goldfuss, 1823) in horizontal and vertical caves of the Bohemian Karst (Czech Republic). *Bull. Geosci.* **81**, 237–276 (2006).
21. Vlček, E. Czechoslovakia. in *Catalogue of fossil hominids, part II: Europe* (eds. Oakley, K., Campbell, B. G. & Molleson, T.) 47–64 (British Museum (Natural History), 1971).
22. Mussi, M. *Earliest Italy: An Overview of the Italian Paleolithic and Mesolithic*. (Springer US, 2002). doi:10.1007/b110672.
23. Lisiecki, L. E. & Raymo, M. E. A Pliocene-Pleistocene stack of 57 globally distributed benthic δ18O records. *Paleoceanography* **20**, (2005).
24. Pettitt, P. & White, M. *The British Palaeolithic : Human Societies at the Edge of the Pleistocene World*. (Routledge, 2012). doi:10.4324/9780203141441.
25. Stárka, V. Life under the Earth. From Karst Diaries of Wabi Stárka. (in Czech) Život v podzemí. Z krasových deníků Wabiho Starky. in *Archaeology and caves. Dedicated to the memory of František Prošek, archaeologist (1922-1958). Archeologie a jeskyně: sborník věnovaný památce archeologa Františka Proška (1922-1958)* (ed. Cílek, V.) 7–41 (1997).
26. Prošek, F., Ložek, V., Hokr, Z. & Vlček, E. The report on the research of Pleistocene deposits at Golden Horse near Koněprusy. (in Czech) Zpráva o výzkumu pleistocenních uloženin na Zlatém Koni u Koněprus. *Věstník Ústředního ústavu geologického* **27**, 254–257 (1952).
27. Churchill, S., Formicola, V., Holliday, T., Holt, B. & Schumann, B. The Upper Palaeolithic population of Europe in an evolutionary perspective. in *Analecta Praehistorica Leidensia 31 / Hunters of the Golden Age : the mid upper palaeolithic of Eurasia : 30,000-20,000 BP* 31–58 (Faculty of Archaeology, University of Leiden, 1999).
28. Lysenko, V. The contribution to stratigraphy of sediments in Koněprusy Caves. (in Czech with German Summary) Příspěvek ke stratigrafii sedimentů v Koněpruských jeskyních. *Český kras* **1**, 18–27 (1976).

29. Svoboda, J. A., van der Plicht, J., Vlček, E. & Kuželka, V. New radiocarbon datings of human fossils from caves and rockshelters in Bohemia (Czech Republic). *Anthropologie (Brno)* **42**, 161–166 (2004).
30. Suchý, V. *et al.* The geochronological and geochemical research of spelean sinters from the Bohemian Karst: New results. (in Czech with English summary) Geochronologické a geochemické výzkumy jeskynních sintrů Českého krasu: Nové výsledky. *ZGV* **34**, 90–93 (2001).
31. Deviese, T., Comeskey, D., McCullagh, J., Ramsey, C. B. & Higham, T. New protocol for compound-specific radiocarbon analysis of archaeological bones. *Rapid Communications in Mass Spectrometry* **32**, 373–379 (2018).
32. McCullagh, J. S. O., Marom, A. & Hedges, R. E. M. Radiocarbon Dating of Individual Amino Acids from Archaeological Bone Collagen. *Radiocarbon* **52**, 620–634 (2010).
33. Jayalakshmi, V., Selvavathi, V., Sekar, M. S. & Sairam, B. Characterization of paraffin waxes by DSC and high temperature GC. *Petroleum Science and Technology* **17**, 843–856 (1999).
34. Nalli, S., Horn, O. J., Grochowalski, A. R., Cooper, D. G. & Nicell, J. A. Origin of 2-ethylhexanol as a VOC. *Environmental Pollution* **140**, 181–185 (2006).
35. Brock, F., Higham, T., Ditchfield, P. & Ramsey, C. B. Current Pretreatment Methods for AMS Radiocarbon Dating at the Oxford Radiocarbon Accelerator Unit (ORAU). *Radiocarbon* **52**, 103–112 (2010).
36. Higham, T. F. G., Jacobi, R. M. & Ramsey, C. B. AMS Radiocarbon Dating of Ancient Bone Using Ultrafiltration. *Radiocarbon* **48**, 179–195 (2006).
37. Schellmann, N. C. Animal glues: a review of their key properties relevant to conservation. *Studies in Conservation* **52**, 55–66 (2007).
38. Johnson, J. S. Consolidation of Archaeological Bone: A Conservation Perspective. *Journal of Field Archaeology* **21**, 221–233 (1994).
39. Bronk Ramsey, C. Bayesian Analysis of Radiocarbon Dates. *Radiocarbon* **51**, 337–360 (2009).

40. Reimer, P. J. *et al.* The IntCal20 Northern Hemisphere Radiocarbon Age Calibration Curve (0–55 cal kBP). *Radiocarbon* **62**, 725–757 (2020).
41. Vågene, Å. J. *et al.* Salmonella enterica genomes from victims of a major sixteenth-century epidemic in Mexico. *Nature Ecology & Evolution* **2**, 520–528 (2018).
42. Peltzer, A. *et al.* EAGER: efficient ancient genome reconstruction. *Genome Biology* **17**, 60 (2016).
43. Jónsson, H., Ginolhac, A., Schubert, M., Johnson, P. L. F. & Orlando, L. mapDamage2.0: fast approximate Bayesian estimates of ancient DNA damage parameters. *Bioinformatics* **29**, 1682–1684 (2013).
44. Sawyer, S., Krause, J., Guschanski, K., Savolainen, V. & Pääbo, S. Temporal patterns of nucleotide misincorporations and DNA fragmentation in ancient DNA. *PLoS ONE* **7**, e34131 (2012).
45. Verdugo, M. P. *et al.* Ancient cattle genomics, origins, and rapid turnover in the Fertile Crescent. *Science* **365**, 173–176 (2019).
46. Tamura, K., Stecher, G., Peterson, D., Filipski, A. & Kumar, S. MEGA6: Molecular Evolutionary Genetics Analysis version 6.0. *Mol. Biol. Evol.* **30**, 2725–2729 (2013).
47. Li, H. & Durbin, R. Fast and accurate short read alignment with Burrows-Wheeler transform. *Bioinformatics* **25**, 1754–1760 (2009).
48. Renaud, G., Slon, V., Duggan, A. T. & Kelso, J. Schmutzi: estimation of contamination and endogenous mitochondrial consensus calling for ancient DNA. *Genome Biology* **16**, 224 (2015).
49. Furtwängler, A. *et al.* Ratio of mitochondrial to nuclear DNA affects contamination estimates in ancient DNA analysis. *Sci Rep* **8**, 1–8 (2018).
50. Vianello, D. *et al.* HAPLOFIND: A New Method for High-Throughput mtDNA Haplogroup Assignment. *Human Mutation* **34**, 1189–1194 (2013).
51. Drummond, A. & Rambaut, A. BEAST: Bayesian evolutionary analysis by sampling trees. *BMC Evolutionary Biology* **7**, 214 (2007).
52. Edgar, R. C. MUSCLE: a multiple sequence alignment method with reduced time and space complexity. *BMC Bioinformatics* **5**, 113 (2004).

53. Keane, T. M., Creevey, C. J., Pentony, M. M., Naughton, T. J. & Mclnerney, J. O. Assessment of methods for amino acid matrix selection and their use on empirical data shows that ad hoc assumptions for choice of matrix are not justified. *BMC Evol. Biol.* **6**, 29 (2006).
54. Drummond, A. J., Suchard, M. A., Xie, D. & Rambaut, A. Bayesian Phylogenetics with BEAUti and the BEAST 1.7. *Mol Biol Evol* **29**, 1969–1973 (2012).
55. Posth, C. *et al.* Pleistocene Mitochondrial Genomes Suggest a Single Major Dispersal of Non-Africans and a Late Glacial Population Turnover in Europe. *Curr. Biol.* **26**, 827–833 (2016).
56. Fu, Q. *et al.* An early modern human from Romania with a recent Neanderthal ancestor. *Nature* **524**, 216–219 (2015).
57. Enattah, N. S. *et al.* Identification of a variant associated with adult-type hypolactasia. *Nat. Genet.* **30**, 233–237 (2002).
58. Sturm, R. A. *et al.* A Single SNP in an Evolutionary Conserved Region within Intron 86 of the HERC2 Gene Determines Human Blue-Brown Eye Color. *Am J Hum Genet* **82**, 424–431 (2008).
59. Kamberov, Y. G. *et al.* Modeling Recent Human Evolution in Mice by Expression of a Selected EDAR Variant. *Cell* **152**, 691–702 (2013).
60. Soejima, M. & Koda, Y. Population differences of two coding SNPs in pigmentation-related genes SLC24A5 and SLC45A2. *Int. J. Legal Med.* **121**, 36–39 (2007).
61. Prüfer, K. *et al.* The complete genome sequence of a Neandertal from the Altai Mountains. *Nature* **505**, 43–49 (2014).
62. Nakatsuka, N. *et al.* ContamLD: estimation of ancient nuclear DNA contamination using breakdown of linkage disequilibrium. *Genome Biology* **21**, 199 (2020).
63. Peyrégne, S. & Peter, B. M. AuthentiCT: a model of ancient DNA damage to estimate the proportion of present-day DNA contamination. *Genome Biology* **21**, 246 (2020).
64. Fu, Q. *et al.* The genome sequence of a 45,000-year-old modern human from western Siberia. *Nature* **514**, 445–449 (2014).
65. Fu, Q. *et al.* The genetic history of Ice Age Europe. *Nature* **534**, 200–205 (2016).

66. Yang, M. A. *et al.* 40,000-Year-Old Individual from Asia Provides Insight into Early Population Structure in Eurasia. *Current Biology* **27**, 3202-3208.e9 (2017).
67. Lipson, M. & Reich, D. A Working Model of the Deep Relationships of Diverse Modern Human Genetic Lineages Outside of Africa. *Molecular Biology and Evolution* **34**, 889–902 (2017).
68. Petr, M., Pääbo, S., Kelso, J. & Vernot, B. Limits of long-term selection against Neandertal introgression. *Proc. Natl. Acad. Sci. U.S.A.* **116**, 1639–1644 (2019).
69. Prüfer, K. *et al.* A high-coverage Neandertal genome from Vindija Cave in Croatia. *Science* **358**, 655–658 (2017).
70. Patterson, N. *et al.* Ancient Admixture in Human History. *Genetics* **192**, 1065–1093 (2012).
71. Reich, D., Thangaraj, K., Patterson, N., Price, A. L. & Singh, L. Reconstructing Indian Population History. *Nature* **461**, 489–494 (2009).
72. Mallick, S. *et al.* The Simons Genome Diversity Project: 300 genomes from 142 diverse populations. *Nature* **538**, 201–206 (2016).
73. Harris, R. S. Improved Pairwise Alignment of Genomic DNA. (Pennsylvania State University, 2007).
74. Sikora, M. *et al.* Ancient genomes show social and reproductive behavior of early Upper Paleolithic foragers. *Science* **358**, 659–662 (2017).
75. Seguin-Orlando, A. *et al.* Genomic structure in Europeans dating back at least 36,200 years. *Science* **346**, 1113–1118 (2014).
76. Lazaridis, I. *et al.* Ancient human genomes suggest three ancestral populations for present-day Europeans. *Nature* **513**, 409–413 (2014).
77. Hajdinjak, M. *et al.* Reconstructing the genetic history of late Neanderthals. *Nature* **555**, 652–656 (2018).
78. 1000 Genomes Project Consortium. A global reference for human genetic variation. *Nature* **526**, 68–74 (2015).
79. Locke, D. P. *et al.* Comparative and demographic analysis of orangutan genomes. *Nature* **469**, 529–533 (2011).

80. Scally, A. *et al.* Insights into hominid evolution from the gorilla genome sequence. *Nature* **483**, 169–175 (2012).
81. Prüfer, K. *et al.* The bonobo genome compared with the chimpanzee and human genomes. *Nature* **486**, 527–531 (2012).
82. Chimpanzee Sequencing and Analysis Consortium. Initial sequence of the chimpanzee genome and comparison with the human genome. *Nature* **437**, 69–87 (2005).
83. Gibbs, R. A. *et al.* Evolutionary and biomedical insights from the rhesus macaque genome. *Science* **316**, 222–34 (2007).
84. Hinch, A. G. *et al.* The landscape of recombination in African Americans. *Nature* **476**, 170–175 (2011).
85. Kong, A. *et al.* Fine-scale recombination rate differences between sexes, populations and individuals. *Nature* **467**, 1099–1103 (2010).
86. Elfessi, A. & Reineke, D. M. A Bayesian Look at Classical Estimation: The Exponential Distribution. *Journal of Statistics Education* **9**, null (2001).
87. Wikipedia. Exponential distribution. *Wikipedia* (2020).
88. Vernot, B. *et al.* Excavating Neandertal and Denisovan DNA from the genomes of Melanesian individuals. *Science* **352**, 235–239 (2016).
89. Peter, B. M. 100,000 years of gene flow between Neandertals and Denisovans in the Altai mountains. *bioRxiv* 2020.03.13.990523 (2020) doi:10.1101/2020.03.13.990523.
90. Moorjani, P. *et al.* A genetic method for dating ancient genomes provides a direct estimate of human generation interval in the last 45,000 years. *Proc. Natl. Acad. Sci. U.S.A.* **113**, 5652–5657 (2016).
