# Supplementary material for: A genome sequence from a modern human skull over 45,000 years old from Zlatý kůň in Czechia
Source: Nat Ecol Evol. 2021 Apr 7;5(6):820–5. doi: 10.1038/s41559-021-01443-x (PMC8175239; doi:10.1038/s41559-021-01443-x)
Supplement: Supplementary file 2 — Reporting Summary [file 41559_2021_1443_MOESM2_ESM.pdf]

nature research

Corresponding author(s): Kay Prüfer, Cosimo Posth, Johannes Krause

Last updated by author(s): 16.02.2021

Reporting Summary

Nature Research wishes to improve the reproducibility of the work that we publish. This form provides structure for consistency and transparency in reporting. For further information on Nature Research policies, see our Editorial Policies and the Editorial Policy Checklist.

Statistics

For all statistical analyses, confirm that the following items are present in the figure legend, table legend, main text, or Methods section.

n/a	Confirmed	
☐	☒	The exact sample size (*n*) for each experimental group/condition, given as a discrete number and unit of measurement
☐	☒	A statement on whether measurements were taken from distinct samples or whether the same sample was measured repeatedly
☐	☒	The statistical test(s) used AND whether they are one- or two-sided<br>*Only common tests should be described solely by name; describe more complex techniques in the Methods section.*
☒	☐	A description of all covariates tested
☐	☒	A description of any assumptions or corrections, such as tests of normality and adjustment for multiple comparisons
☐	☒	A full description of the statistical parameters including central tendency (e.g. means) or other basic estimates (e.g. regression coefficient) AND variation (e.g. standard deviation) or associated estimates of uncertainty (e.g. confidence intervals)
☐	☒	For null hypothesis testing, the test statistic (e.g. *F*, *t*, *r*) with confidence intervals, effect sizes, degrees of freedom and *P* value noted<br>*Give P values as exact values whenever suitable.*
☐	☒	For Bayesian analysis, information on the choice of priors and Markov chain Monte Carlo settings
☒	☐	For hierarchical and complex designs, identification of the appropriate level for tests and full reporting of outcomes
☒	☐	Estimates of effect sizes (e.g. Cohen's *d*, Pearson's *r*), indicating how they were calculated

Our web collection on statistics for biologists contains articles on many of the points above.

Software and code

Policy information about availability of computer code

Data collection

bcl2fastq v2.20
dnaclust v3.0.0
fastqc v0.11.4
EAGER v1.92.59
AdapterRemoval v2.3.1
dedup v0.12.2
mapDamage v2.0.9
BWA v0.7.12
samtools v1.3
pileupCaller v8.6.5

Data analysis	schmutzi<br>AuthentiCT<br>Haplofind<br>muscle<br>MEGA6<br>BEAST v1.8.2<br>ModelGenerator v.85<br>contamLD<br>admixtool 5.1 (qp3Pop, qpDstat, qpGraph, qpF4Ratio)<br>Neanderthal-dating<br>admixfrog 0.6.2-dev3<br>R version 3.3.0 and version 3.4.4

For manuscripts utilizing custom algorithms or software that are central to the research but not yet described in published literature, software must be made available to editors and reviewers. We strongly encourage code deposition in a community repository (e.g. GitHub). See the Nature Research guidelines for submitting code & software for further information.

Data

Policy information about availability of data

All manuscripts must include a data availability statement. This statement should provide the following information, where applicable:

- Accession codes, unique identifiers, or web links for publicly available datasets
- A list of figures that have associated raw data
- A description of any restrictions on data availability

All sequence data generated and analysed during the current study are available in the European Nucleotide Archive under the study accession number PRJEB39040.

Field-specific reporting

Please select the one below that is the best fit for your research. If you are not sure, read the appropriate sections before making your selection.

☒ Life sciences ☐ Behavioural & social sciences ☐ Ecological, evolutionary & environmental sciences

For a reference copy of the document with all sections, see nature.com/documents/nr-reporting-summary-flat.pdf

Life sciences study design

All studies must disclose on these points even when the disclosure is negative.

Sample size	No statistical methods were used to determine ancient DNA sample size a priori.
Data exclusions	No data was excluded.
Replication	Replication is achieved by comparing the results obtained with the shotgun and the nuclear capture datasets.
Randomization	No randomization was performed since the newly generated data derive from a single ancient individual.
Blinding	No blinding was performed. The data was not grouped for analysis.

Reporting for specific materials, systems and methods

We require information from authors about some types of materials, experimental systems and methods used in many studies. Here, indicate whether each material, system or method listed is relevant to your study. If you are not sure if a list item applies to your research, read the appropriate section before selecting a response.

Materials & experimental systems

n/a	Involved in the study	
☒	☐	Antibodies
☒	☐	Eukaryotic cell lines
☐	☒	Palaeontology and archaeology
☒	☐	Animals and other organisms
☒	☐	Human research participants
☒	☐	Clinical data
☒	☐	Dual use research of concern

Methods

n/a	Involved in the study	
☒	☐	ChIP-seq
☒	☐	Flow cytometry
☒	☐	MRI-based neuroimaging

Palaeontology and Archaeology

Specimen provenance	The specimen was provided by the National Museum in Prague, Czechia.
Specimen deposition	The specimen is deposited at the Department of Anthropology of the National Museum in Prague, Czechia.
Dating methods	Three new radiocarbon dates were obtained from the right zygomatic bone: 1) Collagen ultrafiltration dating at the Klaus Tschira Archaeometry Center in Mannheim, Germany; 2) Amino acid hydroxyproline dating at the Oxford Radiocarbon Accelerator Unit in Oxford, UK; 3) Sample pretreatment (solvent wash, decalcification in acid, base wash, re-acidification and gelatinization) and ultrafiltration dating at the Oxford Radiocarbon Accelerator Unit in Oxford, UK.

☒ Tick this box to confirm that the raw and calibrated dates are available in the paper or in Supplementary Information.

Ethics oversight	The Department of Anthropology of the National Museum in Prague, Czechia approved and provided guidance on the study protocol.

Note that full information on the approval of the study protocol must also be provided in the manuscript.
